# High-throughput extraction and quantification method for targeted metabolomics in murine tissues

**DOI:** 10.1007/s11306-017-1312-x

**Published:** 2017-12-30

**Authors:** Sven Zukunft, Cornelia Prehn, Cornelia Röhring, Gabriele Möller, Martin Hrabě de Angelis, Jerzy Adamski, Janina Tokarz

**Affiliations:** 1 Helmholtz Zentrum München, German Research Center for Environmental Health, Institute of Experimental Genetics, Genome Analysis Center, Ingolstaedter Landstrasse 1, 85764 Neuherberg, Germany; 2 German Center for Diabetes Research (DZD), 85764 Neuherberg, Germany; 3 Biocrates Life Sciences AG, Eduard-Bodem-Gasse 8, 6020 Innsbruck, Austria; 4 Lehrstuhl für Experimentelle Genetik, Technische Universität München, 85350 Freising-Weihenstephan, Germany

**Keywords:** Metabolomics, Tissue extraction, Lipids, Amino acids, Biogenic amines, Acylcarnitines

## Abstract

**Introduction:**

Global metabolomics analyses using body fluids provide valuable results for the understanding and prediction of diseases. However, the mechanism of a disease is often tissue-based and it is advantageous to analyze metabolomic changes directly in the tissue. Metabolomics from tissue samples faces many challenges like tissue collection, homogenization, and metabolite extraction.

**Objectives:**

We aimed to establish a metabolite extraction protocol optimized for tissue metabolite quantification by the targeted metabolomics Absolute*IDQ*™ p180 Kit (Biocrates). The extraction method should be non-selective, applicable to different kinds and amounts of tissues, monophasic, reproducible, and amenable to high throughput.

**Methods:**

We quantified metabolites in samples of eleven murine tissues after extraction with three solvents (methanol, phosphate buffer, ethanol/phosphate buffer mixture) in two tissue to solvent ratios and analyzed the extraction yield, ionization efficiency, and reproducibility.

**Results:**

We found methanol and ethanol/phosphate buffer to be superior to phosphate buffer in regard to extraction yield, reproducibility, and ionization efficiency for all metabolites measured. Phosphate buffer, however, outperformed both organic solvents for amino acids and biogenic amines but yielded unsatisfactory results for lipids. The observed matrix effects of tissue extracts were smaller or in a similar range compared to those of human plasma.

**Conclusion:**

We provide for each murine tissue type an optimized high-throughput metabolite extraction protocol, which yields the best results for extraction, reproducibility, and quantification of metabolites in the p180 kit. Although the performance of the extraction protocol was monitored by the p180 kit, the protocol can be applicable to other targeted metabolomics assays.

**Electronic supplementary material:**

The online version of this article (10.1007/s11306-017-1312-x) contains supplementary material, which is available to authorized users.

## Introduction

The dysregulation of metabolic processes is integral to many diseases like cancer, diabetes, or cardiovascular diseases (Kaddurah-Daouk et al. 2008). The application of metabolomics in studies related to human health, in which mostly body fluids were analyzed, becomes more and more frequent and has provided already valuable results and biomarkers for disease outcomes (Adamski 2016; Fan et al. 2016; Roberts et al. 2014). In the majority of metabolomics studies plasma, serum, or urine are used since these body fluids are easily accessible and can be collected either minimal invasively or non-invasively. The metabolomic analyses of body fluids provide a global and systemic view of all biochemical processes in the body, because the interaction of many simultaneously occurring processes over tissues and cell types are reflected (Römisch-Margl et al. 2012). However, when analyzing only the body fluids, metabolic alterations being present in specific tissues may be overseen.

Despite the success of global metabolomics analyses in body fluids, the origin of a disease—and thus, the origin of metabolic changes—often lies in the cellular processes of the tissue (Naz et al. 2014). To characterize metabolic changes underlying a disease phenotype, it is advantageous to analyze metabolite concentrations in tissue samples of animal models or human biopsy samples (Naz et al. 2014). Although tissue collection is always invasive, the pairwise comparison of metabolite profiles taken from diseased and non-diseased tissue regions holds great potential for the understanding of disease mechanisms (Naz et al. 2014; Budczies et al. 2013). However, metabolomics using tissue samples has to overcome many challenges like tissue inhomogeneity, tissue collection, quenching of the metabolism, homogenization of the tissue, and metabolite extraction (Naz et al. 2014). The choice of the sample pretreatment and extraction procedure as well as the choice of the metabolomics measurement method (i.e., targeted or non-targeted metabolomics) can influence both the metabolic features analyzed and the biological interpretation of the obtained data (Naz et al. 2014). With regard to metabolite extraction, different solvents as well as varying extraction protocols (e.g., monophasic versus biphasic extraction) delivered deviating results (Wu et al. 2008; Chen et al. 2013; Le Belle et al. 2002). Since no extraction method will extract all metabolites equally well (Naz et al. 2014; Römisch-Margl et al. 2012) besides fulfilling all requirements regarding to throughput and handling efficiency, any suitable extraction method will be the best compromise for the specific question addressed. Therefore, many different sample preparation protocols and metabolomics measurements from tissue samples were developed previously [reviewed in Naz et al. (2014)].

For the analyses of human plasma by targeted metabolomics, two commercially available kits were developed by Biocrates Life Sciences, namely the Absolute*IDQ*™ p150 and p180 Kits. Both kits quantify a set of acylcarnitines, glycerophospholipids, sphingomyelins, amino acids, and the sum of hexoses by FIA-MS/MS. However, the p180 kit, an extension of the p150 kit, quantifies additionally biogenic amines as well as some further amino acids by including a LC-MS/MS analysis into the method. Both kits have been proven for human plasma to be in conformance with the EMEA guideline “Guideline on bioanalytical method validation (July 21st 2011)” (Committee for Medicinal Products for Human Use (CHMP): Guideline on bioanalytical method validation 2011), which implies proof of reproducibility within a given error range. The p150 kit was shown to be applicable to metabolite quantification in tissue samples in a study by (Römisch-Margl et al. 2012). A comparable proof of principal study has not been performed for the p180 kit up to now.

The aim of this study was thus the development of a metabolite extraction protocol from murine tissues optimized for the metabolite quantification with the Absolute*IDQ*™ p180 Kit. The extraction method should be non-selective for the analytes measured in the p180 kit, applicable to different amounts of input material, monophasic, reproducible, fast, easy, and amenable to high-throughput. We investigated the influence of three different solvents as well as the influence of two different tissue to extraction solvent ratios on the extraction yield, ionization efficiency, and reproducibility of the metabolite quantification. We provide for each tissue type an optimized high-throughput metabolite extraction protocol.

## Materials and methods

### Chemicals

Ammonium acetate (p.a.), formic acid (mass spectrometry grade), phosphate buffer pH 7.5, and phenylisothiocyanate (for protein sequencing) were purchased from Sigma-Aldrich (Hamburg, Germany), methanol (HPLC grade) was purchased from AppliChem (Darmstadt, Germany), acetonitrile (HPLC gradient grade), and pyridine (p.a.) from Roth (Karlsruhe, Germany), and ethanol (HPLC gradient grade) from Merck (Darmstadt, Germany).

### Human reference plasma

Human reference plasma serving as quality control for the metabolite quantification was bought from Seralab (Sera Laboratories International Ltd, Hull, United Kingdom) and consisted of pooled EDTA-plasma from six different volunteers (three male, three female, mean age 31.5 years). Reference samples were split into aliquots and stored at − 80 °C until use. Each reference sample was thawed only once and applied to the kit directly.

### Tissue collection

Male and female mice (C57BL/6 J), aged 10 weeks, were narcotized by isoflurane and all available blood was drawn by retro-orbital sinus puncture as described (Gailus-Durner et al. 2009). After the animals were sacrificed by cervical dislocation, the abdominal skin was moistened with 80% ethanol, opened, and pulled aside. Liver, kidney, skeletal muscle (*M. quadriceps femoris*), visceral fat, brain (cerebrum), pituitary gland, lung, bone (*femur*), adrenal glands, and testis were dissected from male animals, while ovaries were dissected from female animals. All tissues were rinsed with PBS, blotted with lint free tissue paper, immediately snap-frozen in liquid nitrogen, and stored at − 80 °C until further analyses.

### Tissue homogenization and metabolite extraction

Each tissue type was homogenized in three different solvents: (1) 100% methanol (MeOH), (2) 10 mM phosphate buffer pH 7.5 (PB), or (3) an 85/15 (v/v) ethanol/10 mM phosphate buffer pH 7.5 mixture (EtOH/PB). Further, for each tissue type, two defined tissue to solvent ratios were tested [1 mg tissue to X µL solvent, denoted by 1:X (w/v)]. For most tissues, 40–100 mg frozen tissue were mixed in a ratio of 1 mg tissue with either 3 or 6 µL solvent per mg tissue [ratio 1:3 or 1:6 (w/v), respectively]. For bone, 6 or 9 µL solvent per mg tissue [ratio 1:6 or 1:9 (w/v), respectively] were used, because smaller solvent volumes often resulted in damaged tubes during homogenization. Tissues with low input material (e.g., adrenal and pituitary glands, of which only 1–2 mg were available) were homogenized with 12 or 18 µL extraction solvent per mg tissue [ratio 1:12 or 1:18 (w/v), respectively], since at least 10 µL homogenate were needed for the subsequent metabolite quantification. Table 1 provides an overview of all used tissue to solvent ratios.

**Table 1:** Tissue to solvent ratios used for the extraction of metabolites

Tissue type	Tissue to solvent ratio (w/v)
Liver	1:3 and 1:6
Kidney	1:3 and 1:6
Skeletal muscle (*M. quadriceps femoris*)	1:3 and 1:6
Fat (visceral)	1:3 and 1:6
Brain (cerebrum)	1:3 and 1:6
Pituitary gland	1:12 and 1:18
Lung	1:3 and 1:6
Bone	1:6 and 1:9
Adrenal gland	1:12 and 1:18
Testis	1:3 and 1:6
Ovary	1:3 and 1:6

Tissue to solvent ratios are denoted as 1:X, indicating 1 mg of tissue was homogenized with X µL solvent

For homogenization, the frozen tissue pieces were weighed and placed into pre-cooled (dry ice) homogenization tubes containing ceramic beads with a diameter of 1.4 mm (Precellys Homogenization Kit, CK14, PeqLab Biotechnology, Erlangen, Germany). Ice-cold extraction solvent was added to each tube and the tissues were then homogenized in a Precellys24 homogenizer equipped with an integrated cooling unit (PeqLab Biotechnology, Erlangen, Germany) for three times over 20 s at 5500 rpm with 30 s pause intervals to ensure constant temperatures during homogenization. Samples containing organic extraction solvents (MeOH, EtOH/PB) were homogenized at − 4 °C, and samples containing aqueous extraction solvent (PB) at 4 °C. After homogenization, the samples were centrifuged at 4 °C and 2300×*g* for 5 min, and the supernatants (“tissue extracts”) were used for metabolite quantification.

For reproducibility experiments, several portions of the same tissues were homogenized. In case of in-homogenous organs (e.g., kidney, liver, brain), regions of comparable consistency were taken as replicates (e.g., cortex region for kidney). In case the available amount of organ material was very small (pituitary and adrenal glands), organs of littermate mice were used for replicates.

### Quantification of metabolites

For targeted metabolomics analyses of tissue extracts, the Absolute*IDQ*™ p180 Kit (Biocrates Life Sciences, Innsbruck, Austria) was used. The p180 kit is an extension of the p150 kit using an additional liquid chromatographic (LC) separation prior to tandem mass spectrometry measurements (MS/MS). The kit is validated for the use of human plasma in accordance to the EMEA guideline for bioanalytical method validation (Committee for Medicinal Products for Human Use (CHMP): Guideline on bioanalytical method validation 2011) and allows for the simultaneous quantification of 188 metabolites from different compound classes [21 amino acids (AA), 21 biogenic amines (BA), 40 acylcarnitines (AC), 38 acyl/acyl phosphatidylcholines (PCaa), 38 acyl/alkyl phosphatidylcholines (PCae), 14 lyso-phosphatidylcholines (lysoPC), 15 sphingomyelins (SM), and the sum of hexoses (H1)]. The lipids, acylcarnitines, and the hexoses were determined by FIA-MS/MS, while the amino acids and biogenic amines were measured by LC-MS/MS. The metabolites were identified according to MSI Level 1 or 2 (Salek et al. 2013). The complete list of metabolite names, HMDB IDs, and MSI levels of identification is given in the Online Resource, Table S-1.

The sample preparation and measurements were performed according to the manufacturers’ manual of the p180 kit (UM-P180). In detail, internal standards for the LC method were applied to the filter inserts of the 96-well kit plate, which already contained the internal standards (ISTD) for the FIA method. 10 µL of sample (tissue extract, reference plasma, quality controls, zero samples, or calibrators) were added onto the filter inserts and dried for 30 min under a nitrogen stream. Amino acids and biogenic amines were derivatized for 20 min with an excess of 5% phenylisothiocyanate in ethanol/water/pyridine (ratio 1/1/1, v/v/v), and subsequently dried for 45 min under a nitrogen stream. Metabolites and internal standards were then extracted with 300 µL methanol containing 5 mM ammonium acetate by shaking for 30 min, and eluted by centrifugation for 5 min at room temperature and 500×*g*. One-half of the eluate was diluted with water (50/50, v/v) for the LC-MS/MS analysis, and the second half of the eluate was diluted with the kits’ running solvent (1/5, v/v) for FIA-MS/MS analysis.

All liquid handling steps, with the exception of the addition of the tissue extracts to the kit plate, were performed by a Hamilton Micro Lab STAR™ robot (Hamilton Bonaduz, Bonaduz, Switzerland). Evaporation steps were performed using a nitrogen evaporator (Ultravap, Porvair Sciences, Leatherhead, Great Britain), and mass spectrometry analyses were done on an API4000 LC-MS/MS system (ABSciex, Darmstadt, Germany) coupled to an Agilent 1200 Series HPLC (Agilent, Böblingen, Germany), and a HTC PAL autosampler (CTC Analytics, Zwingen, Switzerland) controlled by the Analyst 1.5.1 software (ABSciex, Darmstadt, Germany). Liquid chromatography was performed on a XDBC18 column (3 × 100 mm, 3.5 µm) with a C18 guard column (Agilent, Böblingen, Germany) using acetonitrile and water with 0.2% formic acid as running solvent. For the FIA-MS analyses, the running solvent provided by the kit was used. Separation gradients, mass detection, and quantification were performed as recommended by the manufacturers’ instructions (UM-P180).

On each plate, three plasma samples spiked with different concentrations of reference analytes (QC1-3) and five reference plasma samples (human plasma) were run to serve as quality control and for the evaluation of plate effects, respectively. For each of the three extraction solvents, 10 µL of solvent were transferred three times onto the kit plate and analyzed with internal standards serving as zero samples.

### Data analysis

Data evaluation for the quantification of metabolites and quality assessment was performed with the Met*IDQ*™ software, which is part of the Absolute*IDQ*™ p180 Kit. Amino acids and biogenic amines were quantified absolutely by the help of internal standards and calibration curves consisting of seven calibrator concentrations, while acylcarnitines, glycerophospholipids, and hexoses were evaluated semi-quantitatively by using 13 internal standards for lipids and acylcarnitines and one for the hexoses. The p180 kit has been fully validated by Biocrates for human plasma samples, providing the limit of detection for quantitative and semi-quantitative metabolites (LOD), the lower and upper limit of quantification (LLOQ and ULOQ, respectively), as well as linearity, precision, accuracy, reproducibility, and stability. Despite the p180 kit being validated for the analysis of human plasma only, we used the plasma related parameters for evaluation of our data from tissue extracts.

For the purpose of method optimization, the non-normalized metabolite concentrations measured in the different tissue extracts (reported by Met*IDQ*™ in µM) were compared and evaluated. For the presentation of tissue metabolite concentrations (in pmol/mg tissue) at optimal extraction conditions (Online Resource, Table S-2), the metabolite concentrations measured in tissue extracts were corrected for the dilution factors. Further data analysis was conducted using the software R 3.1.2 (R Core Team 2012). A measured metabolite value was only considered for analysis when the metabolite was found in concentrations above the LOD in all replicates of the respective tissue extract. Heatmaps were constructed using the R package gplots (Warnes et al. 2014) and bar plots were generated using GraphPad Prism 5.

## Results and discussion

The aim of this study was to adapt the Absolute*IDQ*™ p180 Kit from Biocrates, which was primarily developed for metabolite quantification in plasma, to the metabolite analysis in different tissue types. Eleven murine tissues of different types and consistencies, namely liver, kidney, skeletal muscle, visceral fat, brain, pituitary gland, lung, bone, adrenal gland, testis, and ovary were used for this purpose. We especially aimed to provide for each tissue type an optimized high-throughput compatible metabolite extraction protocol, which yields the best results with regard to extractability of metabolites as well as reproducibility in subsequent metabolite quantification with the p180 kit. We therefore tested extraction efficiency and matrix influence on metabolite quantification by applying three different solvents of different polarity and two different tissue to solvent ratios to all of the tissues. The data used for method evaluation are based on metabolite concentrations in the tissue extracts.

### Effect of different extraction solvents on the metabolite extraction efficiency

The p180 kit can detect 188 metabolites from six different chemical classes and a metabolite extraction procedure suitable for use with the kit should efficiently withdraw the metabolites of these classes from the sample matrices. Since the extraction efficiency depends on the chemical nature of the metabolites, we tested metabolite extraction from the tissues with three extraction solvents of different polarity [methanol (MeOH) as a non-polar, phosphate buffer (PB) as a polar, and a mixture of ethanol and phosphate buffer (EtOH/PB) as a mixed-polarity solvent]. The solvents were chosen based on previous findings (Römisch-Margl et al. 2012; Urban et al. 2010).

**Fig. 1:**
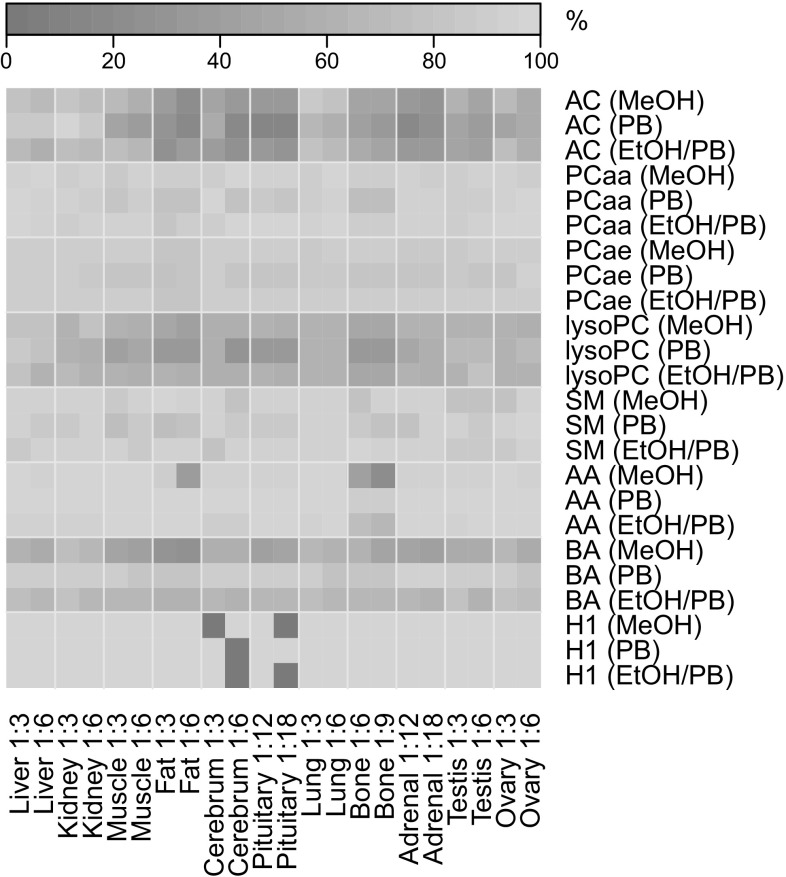
Percentage of quantifiable metabolites per metabolite class in tissue extracts at distinct extraction conditions. Different mouse tissues were homogenized with distinct extraction solvents in different tissue to solvent ratios (5–6 replicates per tissue and extraction condition). Only metabolites showing concentrations above the LOD in all replicates were evaluated. The absolute numbers of metabolites measureable with the p180 kit, abbreviations for metabolite classes, and compositions of extraction solvents are given in Sect. 2. Tissue to solvent ratios are denoted as 1:X, indicating 1 mg of tissue was homogenized with X µL solvent

The efficiency of metabolite extraction from different tissues was assessed on the basis of the absolute numbers of metabolites detectable in tissue extracts with levels above the LOD. As expected, the selection of the extraction solvent had an impact on the number of detected metabolites (Fig. 1). For all lipid species (PC, lysoPC, and SM), we found the extraction efficiency of EtOH/PB to be comparable with that of MeOH, and both solvents to extract more metabolites than PB (Fig. 1). For the hydrophilic metabolite classes (AA, BA, and H1), we observed only slight but no strong extraction solvent dependencies on the metabolite yields (Fig. 1). However, for amino acids and the sum of hexoses, EtOH/PB and PB were slightly superior to MeOH. In case of biogenic amines, we found PB to yield more metabolites with levels above LOD than EtOH/PB and MeOH (in this order). Acylcarnitines were extracted with the highest yield using EtOH/PB and MeOH (Fig. 1); however, the absolute numbers of acylcarnitines detected above the LOD were relatively low for the majority of the tissues analyzed. Mostly medium-chain and some short-chain acylcarnitines acylcarnitines remained below the LOD (data not shown).

The observed extraction efficiencies for lipids and hydrophilic metabolites can be explained by the chemical properties of the metabolites and the solvents and are comparable to previous studies (Urban et al. 2010; Römisch-Margl et al. 2012). The observed lack of certain acylcarnitine species in certain tissues might be due to tissue specific acylcarnitine metabolism. Liver and kidney are the sites of carnitine biosynthesis, while other tissues acquire carnitine from the blood stream (Schooneman et al. 2013). Furthermore, short-chain and specific long-chain acylcarnitines were previously found to be present in large amounts in mouse muscle tissue, while medium-chain acylcarnitines were mainly very low abundant (Chen et al. 2013). This is reflected in our data, as we were able to detect almost all of the acylcarnitines accessible by the p180 kit in mouse liver and kidney but observed only a limited amount of the carnitines in all other tissues including the muscle. Therefore, we assume that the absence of some medium-chain acylcarnitine metabolites in some tissues was due to a very low abundance of acylcarnitines in these tissues. In case of pituitary and adrenal glands, the low input material and the high solvent to tissue ratio certainly have influenced the number of acylcarnitines detected above LOD as well.

### Effect of different tissue to solvent ratios on metabolite extraction efficiency

**Fig. 2:**
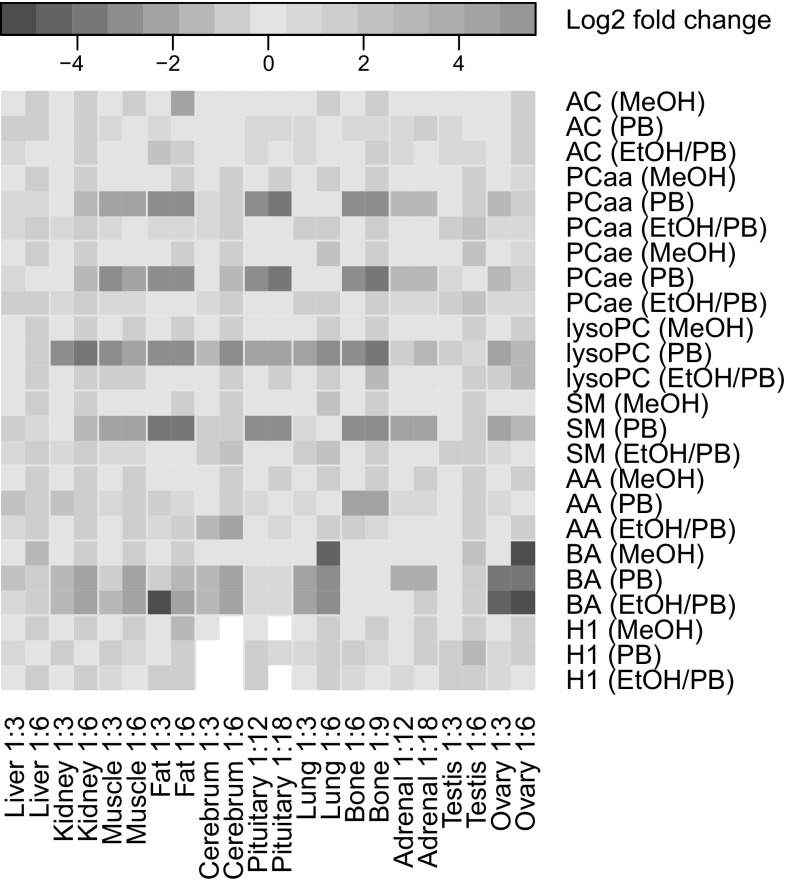
The influence of the extraction solvent and the tissue to solvent ratio on metabolite concentrations in different mouse tissue extracts. Results are shown for 5–6 replicates per tissue and the indicated extraction condition. Log2 fold changes were calculated based on metabolite concentrations in tissue extracts for each tissue type and each metabolite class in relation to the concentrations obtained for the MeOH extracts at the lower tissue to solvent ratio. The fold change of the lower tissue to solvent ratio using MeOH compared to itself (fold change 0) for every tissue and metabolite class is presented in the figure as the yellow square in the upper left corner of each rectangle. White squares indicate that evaluation was not possible due to metabolite concentrations below the LOD. Abbreviations for metabolite classes and composition of extraction solvents are explained in Sect. 2. Tissue to solvent ratios are denoted as 1:X, indicating 1 mg of tissue was homogenized with X µL solvent

The absolute concentration of a metabolite as well as the amount of matrix in a sample affect the metabolites’ ionization performance (Liang et al. 2003; Nilsson and Skansen 2012; Trufelli et al. 2011; Stahnke et al. 2012). Furthermore, a matrix composition different from plasma might influence the derivatization or extraction steps on the p180 kit plate, leading to changes in the observed absolute metabolite concentration. To address these issues, we tested two different tissue to solvent ratios (Table 1) and evaluated the extraction efficiency by determining the absolute numbers and concentrations of metabolites detected above the LOD in the tissue extracts. The absolute numbers of metabolites above LOD were generally higher for all metabolite classes when the tissues were less diluted (i.e., lower tissue to solvent ratios) (Fig. 1). For example, adipose tissue extracted with MeOH yielded more AA above the LOD in the tissue to solvent ratio of 1:3 compared to 1:6 (Fig. 1). Also the metabolite concentrations showed the best overall results (i.e., higher values) for most tissues when the tissues were homogenized using the lower tissue to solvent ratio (Fig. 2 and Online Resource, Fig. S-1). The concentrations of acylcarnitines and lysoPCs were only marginally affected by the amount and kind of the added solvents in most tissues; however, in case of PB the concentrations were slightly higher in the samples of lower tissue to solvent ratios. For the hydrophilic metabolites (AA, BA, and H1), the obtained concentrations were higher in the lower dilution irrespective which solvent was used (Fig. 2). For PCaa, PCae, and SM extracted with MeOH, we observed higher metabolite concentrations with the higher instead of the lower tissue to solvent ratio. Similar but less pronounced effects for those metabolite classes were found using EtOH/PB as extraction solvent (Fig. 2).

The observed higher metabolite concentrations for the hydrophilic metabolites (AA, BA, and H1) in the tissue extracts with lower tissue to solvent ratio can be explained by the lower dilution, whereas the better extractability of the metabolites of the lipid classes at higher dilution (using MeOH or EtOH/PB as extraction solvents) was probably caused by a change in the solvent to water ratio in favor of the organic solvent proportion. Indeed, for our studies we used freshly frozen tissues, which are known to contain a considerable amount of water (Naz et al. 2014) and the addition of a larger amount of e.g. MeOH for extraction changed the ratio of water and organic solvent to a more organic mixture probably facilitating lipid extraction.

### Reproducibility of metabolite quantification from tissue extracts

#### Reproducibility of the extraction procedure

To evaluate the reproducibility of the metabolite extraction, we performed at least five independent replicates for each tissue and each extraction condition. For tissues available in high amounts, the replicates were taken from the same tissue dissection, in case of tissues with low input material (i.e., adrenal and pituitary gland), we used tissue samples from littermate mice. As measures for reproducibility, the coefficients of variation (CVs) of the metabolite concentrations in the replicates of the tissue extracts were calculated.

**Fig. 3:**
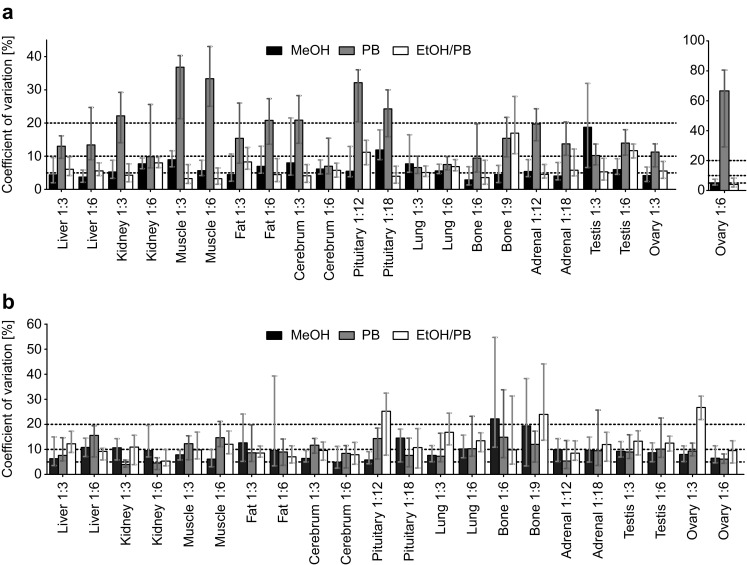
Influence of the extraction solvent and the tissue to solvent ratio on the coefficient of variation (CV). The CVs reflect the reproducibility of homogenization, extraction, metabolite quantification, and biological variability of 5–6 replicates per tissue and extraction condition. The figure presents the median, 20% quantile, and 80% quantile values for all metabolites measured by FIA-MS/MS (lipids, acylcarnitines, hexoses) (**a**), or LC-MS/MS (amino acids, biogenic amines) (**b**). Composition of extraction solvents are given in Sect. 2. Tissue to solvent ratios are denoted as 1:X, indicating 1 mg of tissue was homogenized with X µL solvent

First we analyzed the overall performance of CVs taking all metabolites detected above LOD in all tissues and all tissue to solvent ratios into account (Fig. 3). For the organic solvent containing extraction solvents EtOH/PB and MeOH we found the CVs of 75% of the metabolites to be below 15%; and 65% of the metabolites even displayed CVs below 10%, demonstrating a fairly high reproducibility. The overall CVs obtained with PB were clearly higher and only 60 and 40% of all metabolites showed CVs below 15 and 10%, respectively; thus, the polar solvent performed poorer.

In a second step we looked into more detail and evaluated the reproducibility of the different measurement procedures, FIA-MS/MS and LC-MS/MS, which detect different metabolite classes, i.e. the first method detects lipids, acylcarnitines, and the sum of hexoses, and the second method amino acids and biogenic amines. For FIA-MS/MS measurements (lipids, acylcarnitines, and H1), the use of EtOH/PB yielded the best CVs with 80% of all metabolites having CVs below 15%. When using MeOH or PB only 75 or 50% of the metabolites showed CVs below the 15% threshold (Fig. 3a). For analytes measured by LC-MS/MS (AA and BA), the reproducibility was strongly dependent on the metabolite class (Fig. 3b). Amino acids could be measured with high reproducibility in all solvents, since at least 75% of all AA showed CVs below 15% under all conditions. On the other hand, biogenic amines were determined with best reproducibility using PB. Under these conditions more than 70% of the metabolites displayed CVs below 15%, while with MeOH or EtOH/PB only 30 or 45% of the BA metabolites, respectively, exhibited CVs below 15%. Different tissue to solvent ratios had generally only little impact on the reproducibility; however, exceptions were noted. The extraction of lipids, acylcarnitines, and hexoses from testis using MeOH in the tissue to solvent ratio of 1:6 resulted in clearly lower CV values compared to the tissue to solvent ratio of 1:3 (Fig. 3a). Similarly, the extraction of AA and BA with EtOH/PB from pituitary gland and ovary resulted in lower CV values, when using the higher than the lower tissue to solvent ratio. In contrast, the extraction of AA and BA from fat using MeOH yielded higher CVs using the tissue to solvent ratio of 1:6 compared to 1:3 (Fig. 3b).

The quantification of metabolites in mouse tissues using our metabolite extraction setup and the p180 kit was found to be fairly reproducible, since the majority of the metabolites did not exceed the internationally accepted criterion of a CV of 15% for bioanalytical methods (Committee for Medicinal Products for Human Use (CHMP): Guideline on bioanalytical method validation 2011). Even for tissues with low input material, where littermate mice tissue was used for replicates and additional biological variation was expected, the CVs were not strikingly different. The observed lower reproducibility of lipids in PB is most probably based on the weak extraction efficiency of PB for lipids and is in concordance with previous observations (Urban et al. 2010).

#### Reproducibility of the tissue homogenization step

Physical disruption of the tissue sample is a prerequisite for successful metabolite extraction (Naz et al. 2014). The homogenization using the Precellys24 outperforms classical methods like grinding the tissue sample in liquid nitrogen in many aspects as to sample carryover, feasibility, and throughput (Naz et al. 2014). We assessed the reproducibility of only the tissue homogenization step by analyzing three tissue types either from the same animal (liver and fat) or from littermate animals (bone). Of each of the tissue types six pieces were independently homogenized at optimal extraction conditions, thus, using EtOH/PB as extraction solvent with the tissue to solvent ratios of 1:3 for liver and 1:6 for bone and fat. The median CVs for the detected metabolites were 13% for liver, 16% for fat, and 10% for bone. The overall median CV including all metabolites from all three tissues was found to be below 13% and thereby in the range of the variance of the p180 kit.

The results for our tissue samples homogenized with the Precellys24 were very reproducible, which is in accordance to previous findings (Römisch-Margl et al. 2012; Naz et al. 2014; Wu et al. 2008; Urban et al. 2010). The homogenization performed equally well for all the tissues despite their different properties with respect to heterogeneity, water content, metabolite concentration, and rigidity. Although the quantification of metabolites from different parts of the heterogeneous liver or from bones of littermate mice can potentially increase the biological variation of the measurement (Naz et al. 2014), our data show that our combined homogenization and quantification methods are reproducible and robust.

#### Reproducibility of the sample injection

To determine the technical variance of the metabolite quantification method, the reproducibility of the sample injection was analyzed using tissue extracts from three different tissues. One extract each from liver, fat, and bone homogenized in EtOH/PB at the tissue to solvent ratios of 1:3, 1:6, and 1:6, respectively, processed on the p180 kit plate as usual was applied six times from the same sample well to FIA-MS/MS and six times to LC-MS/MS for metabolite detection and quantification. We obtained CVs below 15% for almost all and CVs below 5% for more than half of all detected metabolites (data not shown), demonstrating very high injection reproducibility. Thus, the step of sample injection is scarcely prone to introduce variation.

### Tissue and solvent dependent ionization efficiency

Alteration in the ionization efficiency of an analyte arises from the concurrent ionization of different compounds in the sample matrix, also known as matrix effect, as well as from the amount of the ionized analyte (Antignac et al. 2005; Trufelli et al. 2011). Matrix effects can cause non-linear responses, lower quantification accuracy, reduced reproducibility, and an altered limit of quantification (Antignac et al. 2005). Matrix effects on ionization efficiency can become visible when the internal standard (ISTD) intensities expressed as peak area in tissue extract samples are compared to those in zero samples (zero samples contain only ISTD and the respective extraction solvent). The ISTD intensities of the zero samples are set to 100% ionization efficiency.

**Fig. 4:**
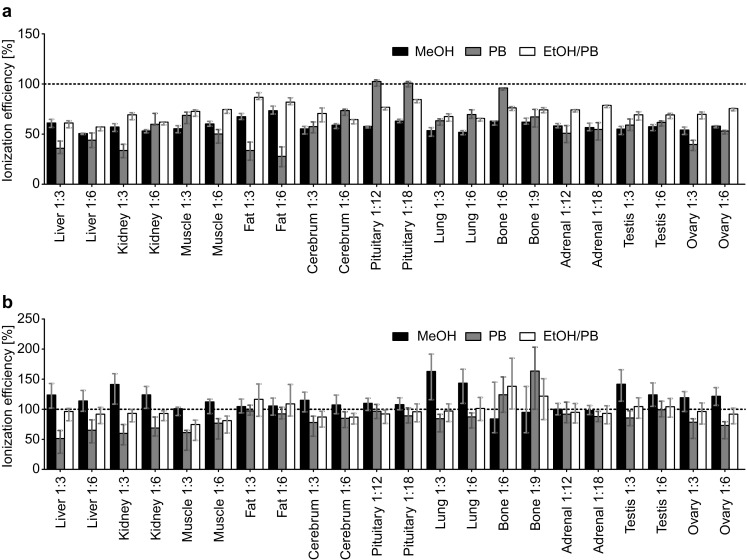
Influence of the extraction solvent and the tissue to solvent ratio on the ionization efficiencies. Results are shown for 5–6 replicates per tissue and the indicated extraction condition. The ionization efficiency was calculated by dividing the signal intensity of the internal standards (ISTDs) in tissue extracts by the intensities of the ISTDs in the respective extraction solvent. The figure presents the median, 20% quantile, and 80% quantile values for all metabolites measured by FIA-MS/MS (lipids, acylcarnitines, hexoses) (**a**), or LC-MS/MS (amino acids, biogenic amines) (**b**). Composition of extraction solvents are explained in Sect. 2. Tissue to solvent ratios are denoted as 1:X, indicating 1 mg of tissue was homogenized with X µL solvent

We evaluated the ionization efficiency for each of the eleven tissues at two different tissue to solvent ratios and the three different extraction conditions. We observed for metabolites measured by FIA-MS/MS (lipids, acylcarnitines, and H1) a general suppression of ionization efficiency (Fig. 4a). Averaged over all extraction solvents and all tissue to solvent ratios, the ionization efficiency of the metabolites was reduced to 60–80%. The lowest ionization efficiency was found for fat extracted with PB (~ 30%) and the highest for pituitary gland and bone extracted with PB (~ 100%) (Fig. 4a). The data obtained for metabolites measured by LC-MS/MS (AA and BA) showed varying ionization efficiencies ranging from 70 to 150% when compared to the zero samples (Fig. 4b). In case of certain tissues like liver, kidney, and ovary, the use of organic extraction solvents (EtOH/PB or MeOH) yielded better ionization efficiencies than the use of the purely polar PB. The ionization efficiency compared within one tissue and one extraction solvent was generally not influenced by the tissue to solvent ratio (Fig. 4).

The ISTD for the BA taurine showed a tremendous ionization efficiency enhancement (> 300% compared to zero samples) in cerebrum, bone, liver, lung, muscle, kidney, and ovary in all three extraction solvents. An enhancement in this dimension is uncommon; however, we explain the observation with the very high concentrations of taurine in murine tissues (> ULOQ; data not shown) which can interfere with the ISTD. The mass transition of taurine’s ISTD is only two Dalton larger than the mass transition for the non-labeled analyte and overlaps with naturally occurring 13C-isotopes of taurine. Thus, the C13-isotopes of taurine can strongly enhance the ISTD signal at very high concentrations of taurine in the sample. To confirm this assumption, we compared respective signal intensities of ISTD-free tissue extracts [liver, kidney, muscle, lung, and testis prepared in MeOH or PB at the tissue to solvent ratio 1:6 (w/v)] with those of the zero sample. We found that the intensities for the mass transition of the ISTD of taurine in most tissue extracts prepared with MeOH or PB were either as high as the taurine ISTD in zero samples (factor 1 for liver) or even higher (factor 1.5, 2, and 3.5 for kidney, lung, and muscle). In testis, the ISTD signal for taurine was less than that in the zero samples (factor 0.5). These results clearly indicate that 13C-isotopes of taurine interfere with the mass transition of the ISTD for taurine in many mouse tissue extracts. No other comparably interfering compounds were identified for all the other ISTDs of the p180 kit. An additional matrix effect influencing the taurine measurement cannot be excluded; however, due to the huge impact of the C13-isotopes, such further effect is not accessible.

The observed deviations for the ionization efficiency were higher for metabolites measured by FIA-MS/MS than for those measured by LC-MS/MS, because the chromatography step in LC-MS/MS has a high capacity to separate analytes and interfering substances (Chambers et al. 2007; Gosetti et al. 2010). Lower solvent to tissue ratios may increase the sensitivity (as metabolite concentrations are higher), but matrix effects due to higher concentration of interfering compounds can be enhanced as well (Stahnke et al. 2012). However, we noted almost no impact of the tissue to solvent ratio on the metabolite ionization efficiencies for all tested tissues and all extraction solvents.

Quantification results for taurine in tissue extracts should be evaluated with great caution. The p180 kit is validated for taurine levels in human plasma, which are in the range of 55 µM (Cynober 2002). The taurine levels in murine tissues exceed this range by at least one order of magnitude (Massie et al. 1989; Ueki et al. 2012). Thus, the measured values for taurine in murine tissue extracts are well above the quantification range provided by the p180 kit and are therefore not reliable. Furthermore, our observation that the 13C-isotopes of taurine interfere with the measurement of the ISTD, hampers the quantification of taurine even more. Although other ISTDs measurements were not affected by large levels of analyte in the murine tissue extracts, it might be a different scenario with tissue of other species. Pilot experiments prior to real studies to discover disturbing effects are therefore highly recommended.

### Ionization efficiency of metabolites extracted from tissue compared to human plasma samples

The p180 kit was originally validated by Biocrates for the use with human plasma. To analyze whether the matrix effects of mouse tissue extracts are comparable to those of human plasma, we calculated the ratio of the ISTD intensity in all tissue extracts to the ISTD intensities of nine human reference plasma samples replicates (Online Resource, Fig. S-2). Values > 1 indicate less and values < 1 indicate more ion suppression in tissue samples compared to human plasma. For lipids, acylcarnitines, and the sum of hexoses measured by FIA-MS/MS, we observed in general less ion suppression in tissue extracts than in plasma (relative intensities in tissues 1.4–4.8), independently of the extraction solvent used. The only exceptions were acylcarnitines extracted with PB from liver and PCaa and PCae extracted with PB from fat, where we found slightly more ion suppression compared to that in human plasma (0.5–0.9). The matrix effects for metabolites analyzed by LC-MS/MS, namely AA and BA, were more diverse. Tissue extracts prepared in MeOH showed similar or marginally more ion suppression than human plasma samples (0.7–1.2). Two metabolites showed a strong deviation from the general trend, namely sarcosine and taurine. Sarcosine was found to be highly suppressed in all tissue extracts (0.3–0.7), while taurine as expected from observations described above showed less ion suppression in all tissues compared to human plasma (3.0–9.0). Using PB as extraction solvent, we found less ion suppression in most tissues (1.0–2.1), but also a slight increase in ion suppression for liver, kidney, and muscle (0.7–0.9). Once again, taurine showed a large enhancement (2.7–9.3) in all tissues compared to human plasma. The matrix effects for EtOH/PB were comparable to the results obtained for MeOH, showing both similar as well as slightly increased ion suppression compared to human plasma (0.6–1.3). In this case, we observed slightly increased suppression for lysine, ornithine, and acetyl-ornithine (0.1–0.6) in all analyzed tissue extracts. As already noted for the ionization efficiency, the tissue to solvent ratio compared within one tissue and one extraction solvent had no striking influence on the relative ionization efficiency of tissue extracts compared to human plasma (Online Resource, Fig. S-2).

In general, the observed matrix effects of tissue extracts were found to be smaller or in a similar range compared to those of human plasma. These results are in accordance with a previous study (Römisch-Margl et al. 2012) and demonstrate that the metabolomics analysis of tissue extracts with the p180 kit is feasible. However, a few metabolites were truly influenced by the tissue matrix and the results for metabolites with increased ion suppression compared to human plasma, i.e., sarcosine, lysine, ornithine, and acetyl-ornithine, should be interpreted with caution. The data for these metabolites may yield non-linear responses or lower quantification accuracy (Antignac et al. 2005). As already discussed above, high levels of taurine led to the interference of one of its 13C-isotopes with the ISTD for taurine in tissue extracts, and therefore, taurine concentrations obtained for tissues with high levels of this analyte should not be considered for further data analyses.

### Further considerations

Metabolomics using tissue extracts includes many challenges for the experimental protocol like tissue homogeneity, tissue collection, homogenization of the tissue, and metabolite extraction (Naz et al. 2014). Although we have addressed many of these issues in our study, further considerations need to be taken into account. One of these issues is the sample collection. For our study, we did not perfuse the collected organs, so traces of blood might have remained in the used tissue samples. Thus, the obtained tissue metabolite concentrations probably reflect both tissue and blood origin. For this reason, our obtained tissue metabolite concentrations (Online Resource, Table S-2) should be compared with care to results from other studies. A recent metabolomics study using perfused and non-perfused murine liver samples showed that some of the investigated metabolites were significantly altered upon perfusion (Ly-Verdú et al. 2013). This indicates that even with respect to a single study, it is mandatory to collect the tissues always in the same manner to avoid variability in metabolomics measurements.

Tissue heterogeneity is known to exist especially for brain, liver, and kidney, and region-specific results might be possible (Naz et al. 2014). We analyzed six pieces of the same liver to address the reproducibility of tissue homogenization and found no region-specific results. Despite this observation, we recommend to always use the same region of a certain tissue to avoid any region-specific outcome.

Normally, during the validation of new methods, the accuracy of these method needs to be analyzed (Committee for Medicinal Products for Human Use (CHMP): Guideline on bioanalytical method validation 2011). However, the accuracy of metabolite extraction from tissues can hardly be determined, because the commonly used approach, namely spiking the sample with known concentrations of an analyte, is not feasible. A spiked metabolite will not incorporate into the cells of the tissue, and thus, the “real” metabolite extraction cannot be simulated. Nonetheless, if the same extraction conditions are applied to all samples of a study, the resulting metabolite concentrations are comparable to each other within one tissue type.

Since we focused on the development of a robust and high-throughput amenable extraction procedure using the Biocrates Absolute*IDQ*™ p180 Kit as read-out, we were not able to validate the extraction methods regarding other metabolites not included into the kit, such as metabolites from the central carbon metabolism or phosphorylated metabolites. This limitation needs to be taken into account upon adaptation of the here presented tissue extraction protocol to other metabolomics approaches like non-targeted measurements. In this case, the quality and robustness of the extraction protocol needs to be re-assessed for the analyzed metabolites.

## Concluding remarks

We have analyzed the applicability of the Biocrates Absolute*IDQ*™ p180 Kit to tissue samples with respect to extraction yield, ionization efficiency, and reproducibility of the metabolite quantification using three different solvents in two tissue to solvent ratios and 11 different murine tissues covering highly different physical properties (i.e., amount of input material, composition, water, fat or connective tissue content, metabolite concentration, rigidity). We found the extraction solvent composition to have a clear impact on the detected metabolite numbers and concentrations, as well as on reproducibility. This impact was strongly metabolite class dependent. Additionally, the tissue to solvent ratio affected the quantification results. Tissue homogenization with the Precellys24 was very reproducible. Overall reproducibility for all metabolites and all tissues was found to be highest when the extraction solvent contained organic solvent. Matrix effects on the ionization efficiency were generally smaller or in a similar range compared to those seen with human plasma.

EtOH/PB as extraction solvent provided the highest number of extracted metabolites together with a high reproducibility for most tissues. MeOH or PB were selective in terms of extractability and reproducibility, and may thus overcome EtOH/PB for analysis of certain metabolite classes. The best performing extraction solvents and tissue to solvent ratios for use with the p180 kit are summarized in Table 2.

**Table 2:** Recommended extraction solvent and tissue to solvent ratios for best metabolite quantification and lowest variation using the Absolute*IDQ*™ p180 Kit

Tissue type	Optimal extraction solvent	Optimal tissue to solvent ratio (w/v)
Liver	EtOH/PB	1:3
Kidney	MeOH	1:3
Skeletal muscle (*M. quadriceps femoris*)	EtOH/PB	1:3
Fat (visceral)	EtOH/PB	1:6
Brain (cerebrum)	EtOH/PB	1:6
Pituitary gland	MeOH	1:12
Lung	EtOH/PB	1:3
Bone	EtOH/PB	1:6
Adrenal gland	EtOH/PB	1:12
Testis	MeOH	1:6
Ovary	EtOH/PB	1:3

Tissue to solvent ratios are denoted as 1:X, indicating 1 mg of tissue was homogenized with X µL solvent. Composition of extraction solvents are given in Sect. 2

We assume that the experimental procedure presented in our study, especially the metabolite extraction, can be adapted to other study designs with tissues from species others than the mouse as well as further tissue types. The information listed in Table 2 is a recommendation that shall help to optimize sample preparation in upcoming studies. However, since metabolomics quantification is influenced by many factors we strongly suggest to always performing a pilot experiment to address general validation parameters like reproducibility prior to the main study.

## Electronic supplementary material

Below is the link to the electronic supplementary material.

Supplementary material 1 (PDF 204 KB)
