# Supplementary material for: High-throughput extraction and quantification method for targeted metabolomics in murine tissues
Source: Metabolomics. 2017 Dec 30;14(1):18. doi: 10.1007/s11306-017-1312-x (PMC5748028; doi:10.1007/s11306-017-1312-x)
Supplement: Supplementary file 1 — Supplementary material 1 (PDF 204 KB) [file 11306_2017_1312_MOESM1_ESM.pdf]

Metabolomics

High-throughput extraction and quantification method for targeted metabolomics in murine tissues

*Sven Zukunft [1,2], Cornelia Prehn [1], Cornelia Röhring [3], Gabriele Möller [1], Martin Hrabě de Angelis [1,2,4], Jerzy Adamski [1,2,4], and Janina Tokarz [1]**

[1] Helmholtz Zentrum München, German Research Center for Environmental Health, Institute of Experimental Genetics, Genome Analysis Center, Ingolstaedter Landstrasse 1, 85764 Neuherberg, Germany
[2] German Center for Diabetes Research (DZD), 85764 Neuherberg, Germany
[3] Biocrates Life Sciences AG, Eduard-Bodem-Gasse 8, A-6020 Innsbruck, Austria
[4] Lehrstuhl für Experimentelle Genetik, Technische Universität München, 85350 Freising-Weihenstephan, Germany

* Corresponding author: Janina Tokarz, Helmholtz Zentrum München, German Research Center for Environmental Health, Institute of Experimental Genetics, Genome Analysis Center, Ingolstaedter Landstrasse 1, 85764 Neuherberg, Germany
Voice: +49-89-3187-3233, Fax: +49-89-3187-3225, E-mail: janina.tokarz@helmholtz-muenchen.de

Table of Contents

Fig. S-1 Influence of the extraction solvent and the tissue to solvent ratio on metabolite concentrations in different mouse tissues. Page S-2

Fig. S-2 Comparison of matrix effects of murine tissue extracts to matrix effects of human plasma. Page S-7

Table S-1 List of analyzed metabolites with their abbreviations, names, metabolite classes, HMDB IDs, and MSI level of metabolite identification. Page S-8

Table S-2 Tissue metabolite concentrations measured at optimal extraction conditions. Page S-31

References Page S-37

Metabolomics

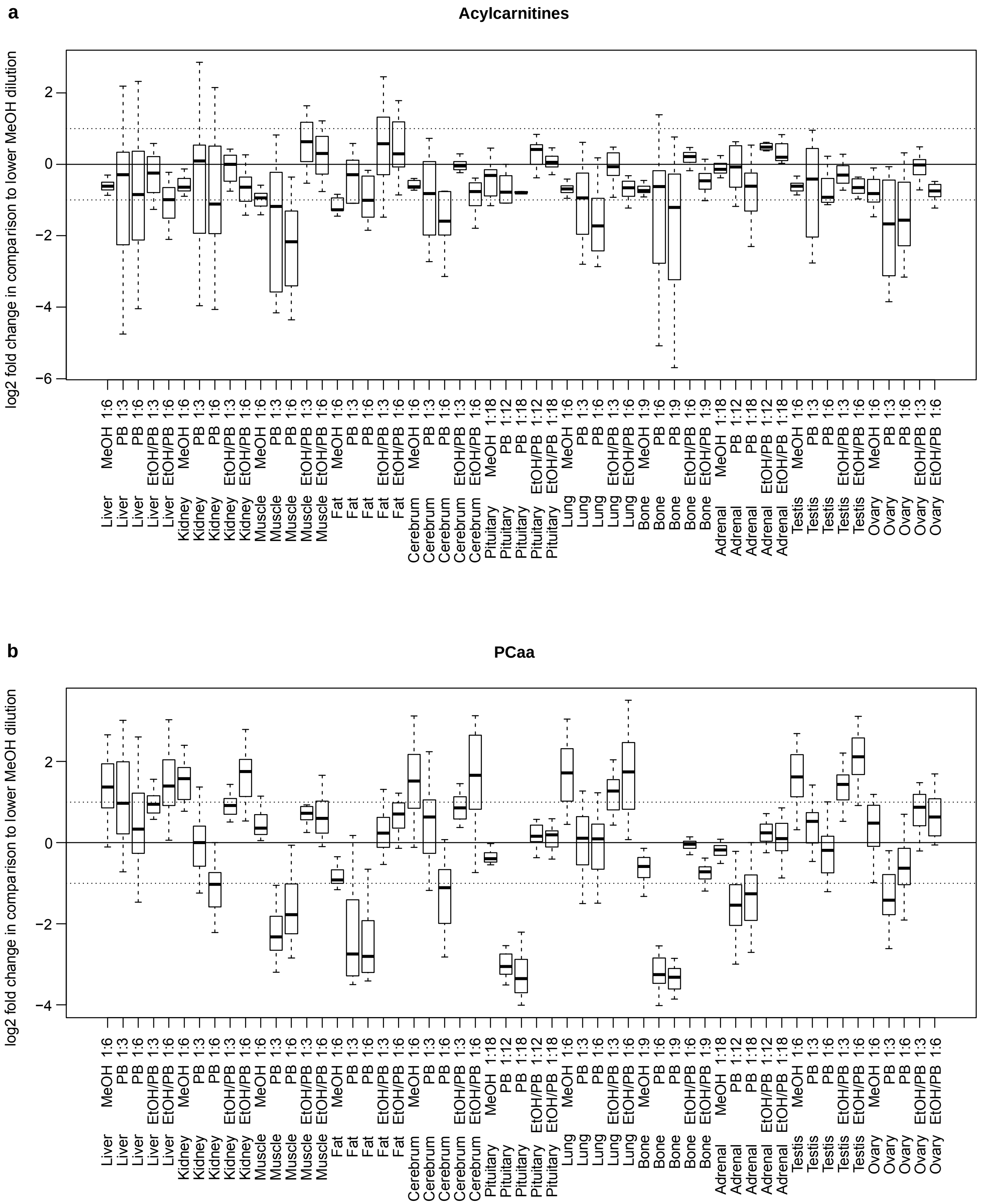

Fig. S-1

c

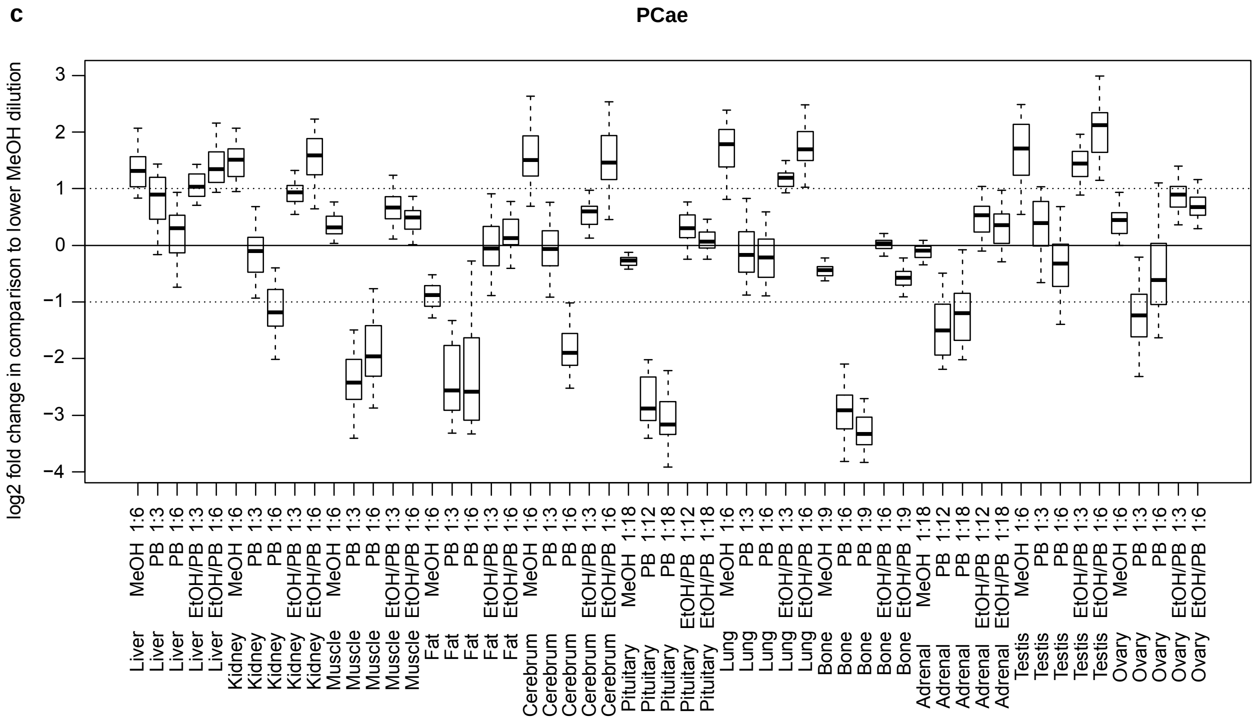

d

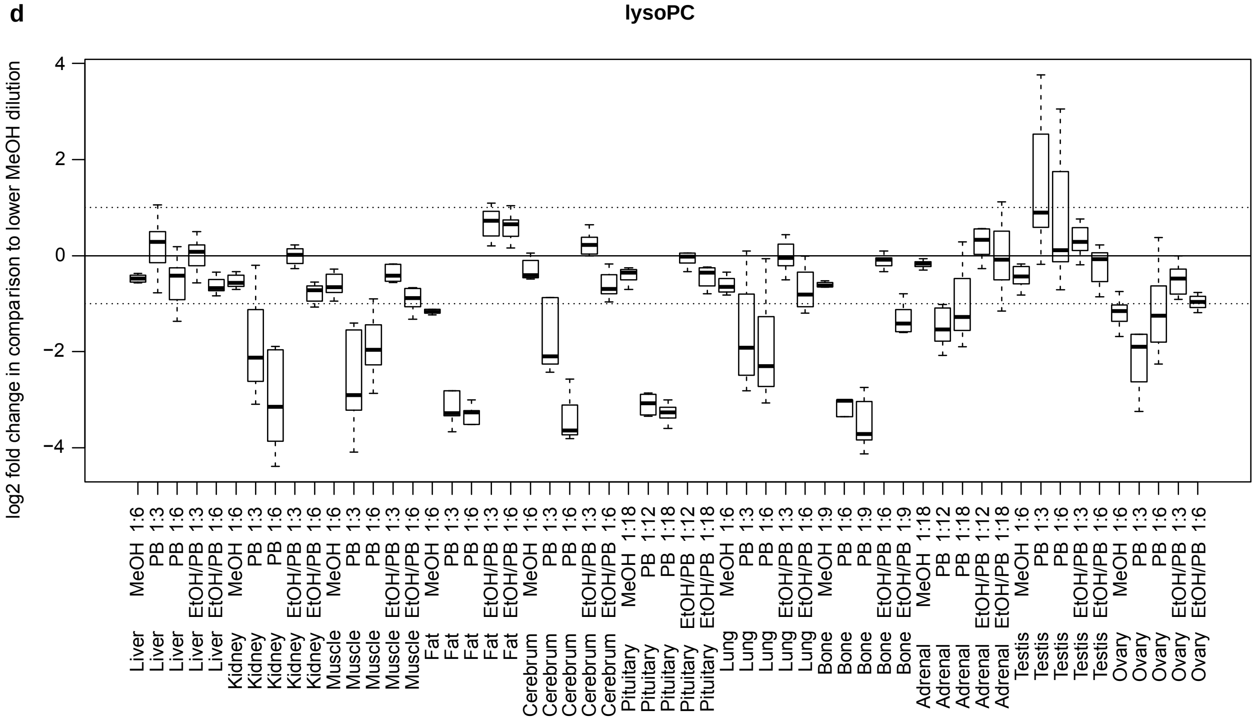

Fig. S-1 (continued)

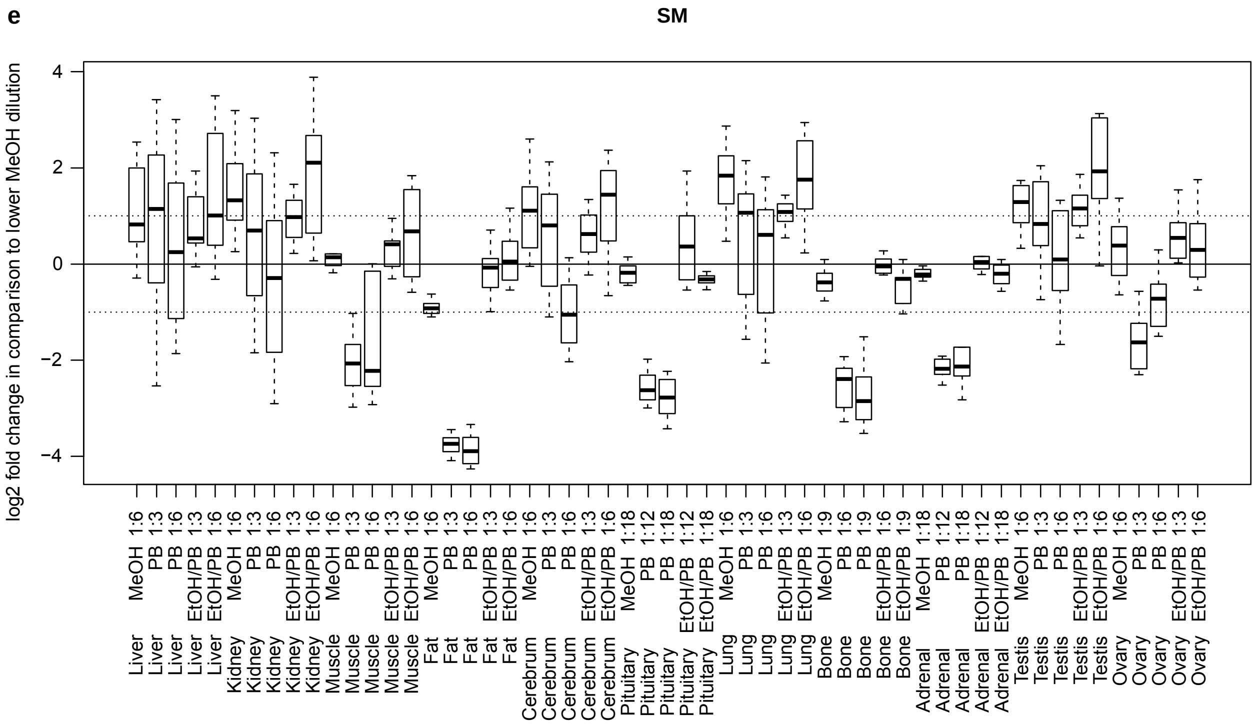

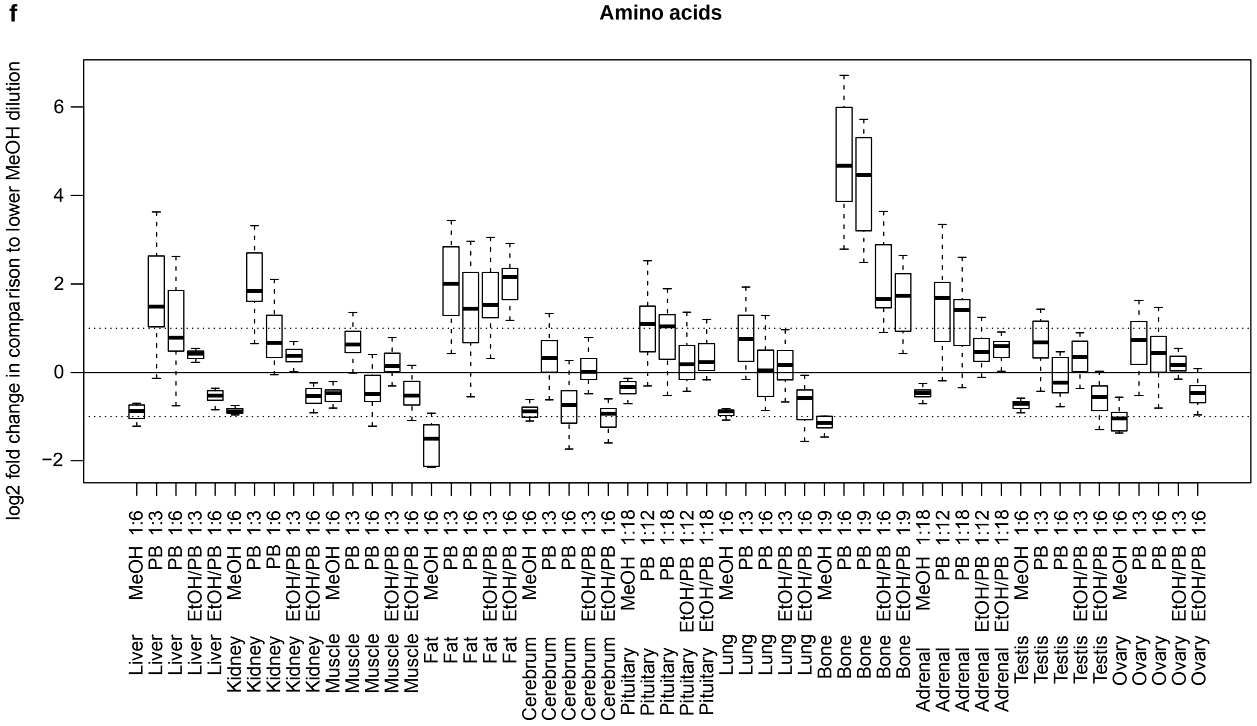

Fig. S-1 (continued)

g

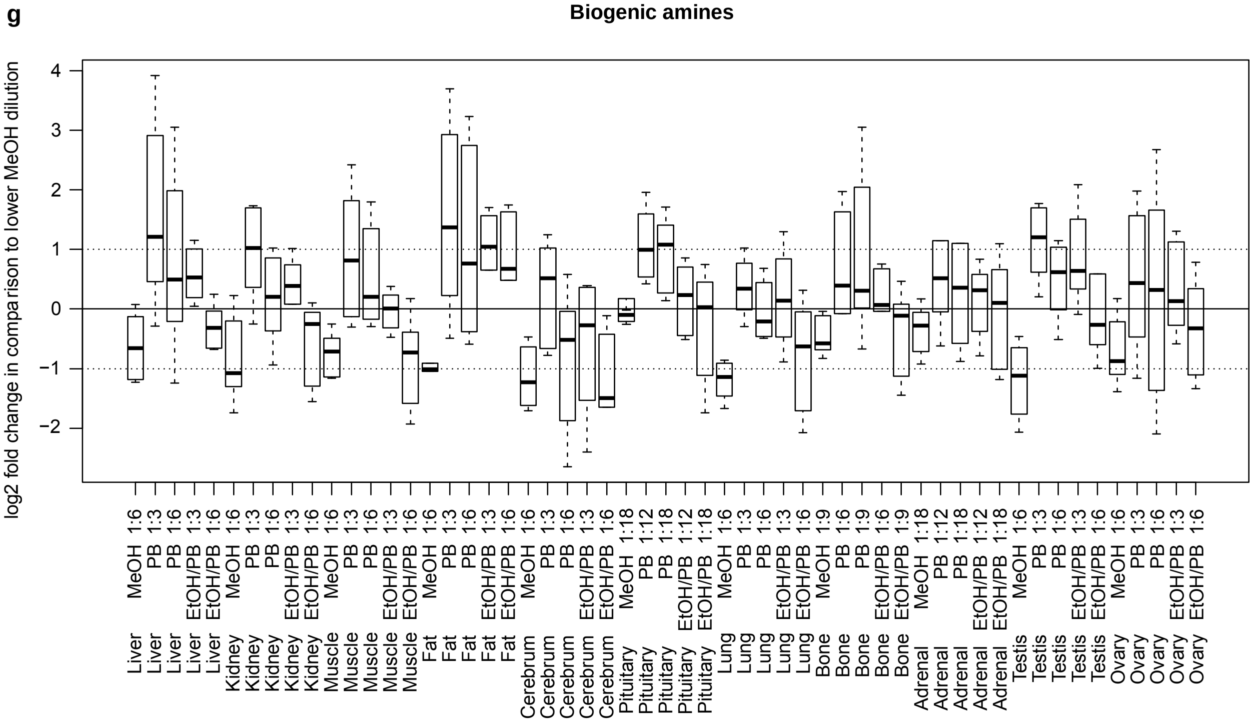

h

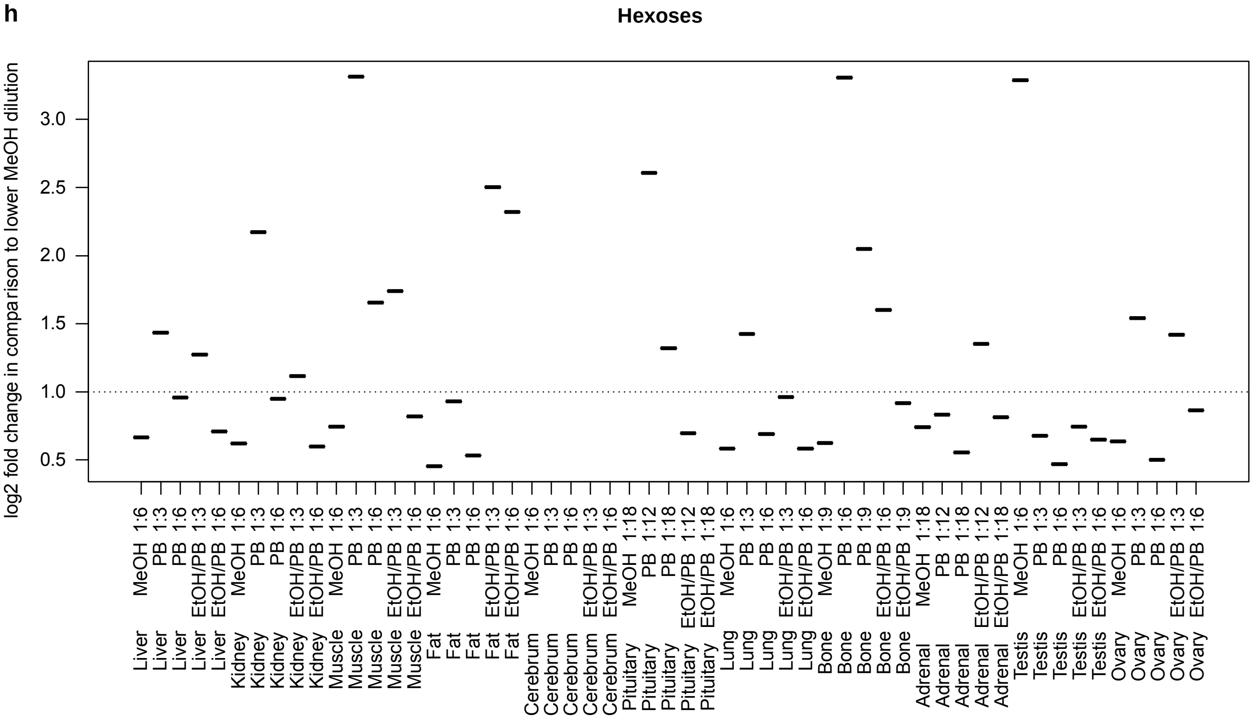

Fig. S-1 (continued)

Fig. S-1 Influence of the extraction solvent and the tissue to solvent ratio on metabolite concentrations in different mouse tissues.

This figure presents the detailed data for Fig. 2 in the manuscript. Log2 fold changes were calculated for each tissue type and each metabolite class in relation to the concentrations obtained for MeOH in the respective lower tissue to solvent ratio. The fold change of the lower tissue to solvent ratio using MeOH compared to itself (fold change 0) for every tissue and metabolite class is not presented in the figure (which is in contrast to Fig. 2 in the manuscript). The figure presents the median, the 25% quartile, and the 75% quartile values for 5-6 replicates per tissue for all metabolite classes, namely for acylcarnitines (a), acyl/acyl phosphatidylcholines (PCaa, b), acyl/alkyl phosphatidylcholines (PCae, c), lyso-phosphatidylcholines (lysoPC, d), sphingomyelins (SM, e), amino acids (f), biogenic amines (g), and the sum of hexoses (h). Missing boxes indicate that the evaluation was not possible due to metabolite concentrations below the LOD. Abbreviations for extraction solvents are as follows: MeOH, 100% methanol; PB, 10 mM phosphate buffer pH 7.5; EtOH/PB, 85/15 (v/v) mixture of ethanol and 10 mM phosphate buffer pH 7.5. Tissue to solvent ratios are denoted as 1:X, indicating 1 mg of tissue was homogenized with X µL solvent.

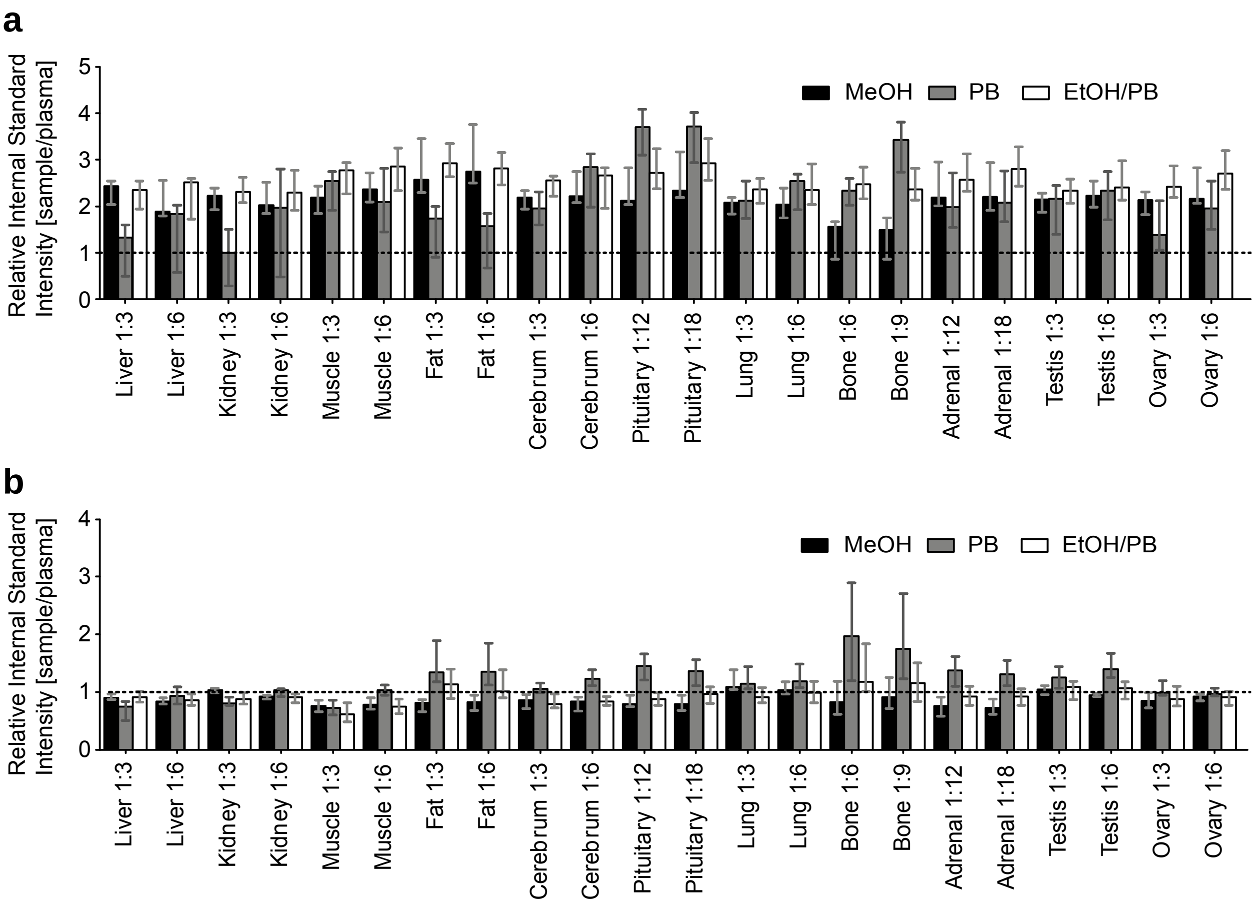

Fig. S-2 Comparison of matrix effects of murine tissue extracts to matrix effects of human plasma.

To determine whether the matrix effects of murine tissue extracts are comparable to the matrix effects of human plasma, we calculated the ratio of the Internal Standard intensity of all tissue extracts (5-6 replicates per tissue and extraction condition) to the Internal Standard intensity of nine human reference plasma samples. Values larger than 1 indicate less ion suppression and values smaller than 1 indicate more ion suppression in tissue samples compared to human plasma. The figure presents the median, the 20% quantile, and the 80% quantile values for all metabolites measured by FIA-MS/MS (lipids, hexoses) (a), or LC-MS/MS (amino acids, biogenic amines) (b). Abbreviations for extraction solvents are as follows: MeOH, 100% methanol; PB, 10 mM phosphate buffer pH 7.5; EtOH/PB, 85/15 (v/v) mixture of ethanol and 10 mM phosphate buffer pH 7.5. Tissue to solvent ratios are denoted as 1:X, indicating 1 mg of tissue was homogenized with X µL solvent.

Table S-1 List of analyzed metabolites with their abbreviations, names, metabolite classes, HMDB IDs, and MSI level of metabolite identification.

The table lists metabolites covered by the analyzed MRMs, their abbreviations, names, metabolite classes, MSI level of metabolite identification, and, if available, HMDB IDs. Based on the MSI standards (Salek et al. 2013), the levels were defined as follows: Level 1 are metabolites, which were measured with LC-MS/MS (two orthogonal properties: retention time and MRM) and which had the same isotopically labeled metabolite as internal standard. Level 2 are metabolites, which were either measured with FIA-MS/MS and/or did not have an identical isotopically labeled internal standard. Chirality was not taken into account for classification.

Abbreviation	Name	Metabolite class	HMDB ID	MSI level
C0	Carnitine	(Acyl)carnitines		Level 2
C0	D-Carnitine	(Acyl)carnitines	HMDB00062	Level 2
C0	L-Carnitine	(Acyl)carnitines		Level 2
C2	Acetylcarnitine	Acylcarnitines	HMDB00201	Level 2
C2	Acetyl-D-carnitine	Acylcarnitines		Level 2
C2	Acetyl-L-carnitine	Acylcarnitines	HMDB00201	Level 2
C3	Propionylcarnitine	Acylcarnitines	HMDB00824	Level 2
C3	Propionyl-L-carnitine	Acylcarnitines	HMDB00824	Level 2
C3-DC (C4-OH)	Malonylcarnitine	Acylcarnitines	HMDB02095	Level 2
C3-DC (C4-OH)	Malonyl-D-carnitine	Acylcarnitines	HMDB02095	Level 2
C3-DC (C4-OH)	Malonyl-L-carnitine	Acylcarnitines	HMDB02095	Level 2
C3-DC (C4-OH)	Hydroxybutyrylcarnitine	Acylcarnitines	HMDB13127	Level 2
C3-OH	Hydroxypropionylcarnitine	Acylcarnitines		Level 2
C3:1	Propenylcarnitine	Acylcarnitines		Level 2
C4	Butyrylcarnitine	Acylcarnitines	HMDB02013	Level 2
C4	Butyryl-L-carnitine	Acylcarnitines	HMDB02013	Level 2
C4	Isobutyrylcarnitine	Acylcarnitines	HMDB00736	Level 2
C4	Isobutyryl-L-carnitine	Acylcarnitines	HMDB00736	Level 2
C4:1	Butenyl-L-carnitine	Acylcarnitines	HMDB13126	Level 2
C5	2-Methylbutyrylcarnitine	Acylcarnitines	HMDB00378	Level 2
C5	Isovalerylcarnitine	Acylcarnitines	HMDB00688	Level 2
C5	Isovalery-L-carnitine	Acylcarnitines	HMDB00688	Level 2
C5	Pivaloylcarnitine	Acylcarnitines		Level 2
C5	Valerylcarnitine	Acylcarnitines	HMDB13128	Level 2
C5	Valeryl-L-carnitine	Acylcarnitines	HMDB13128	Level 2
C5-DC (C6-OH)	Glutarylcarnitine (Hydroxyhexanoylcarnitine)	Acylcarnitines		Level 2
C5-M-DC	Methylglutaryl-L-carnitine	Acylcarnitines	HMDB00552	Level 2
C5-OH (C3-DC-M)	Hydroxyvalerylcarnitine (Methylmalonylcarnitine)	Acylcarnitines		Level 2
C5:1	Tiglylcarnitine	Acylcarnitines	HMDB02366	Level 2
C5:1-DC	Glutaconylcarnitine	Acylcarnitines		Level 2
C6 (C4:1-DC)	Hexanoylcarnitine (Fumarylcarnitine)	Acylcarnitines	HMDB00705	Level 2
C6 (C4:1-DC)	L-Hexanoylcarnitine	Acylcarnitines	HMDB00756	Level 2

Metabolomics

Abbreviation	Name	Metabolite class	HMDB ID	MSI level
C6:1	Hexenoylcarnitine	Acylcarnitines		Level 2
C7-DC	Pimelylcarnitine	Acylcarnitines		Level 2
C8	Octanoylcarnitine	Acylcarnitines	HMDB00791	Level 2
C8	Octanoyl-D-carnitine	Acylcarnitines		Level 2
C8	Octanoyl-L-carnitine	Acylcarnitines	HMDB00791	Level 2
C9	2,6 Dimethylheptanoylcarnitine	Acylcarnitines	HMDB06320	Level 2
C10	Decanoylcarnitine	Acylcarnitines	HMDB00651	Level 2
C10	Decanoyl-L-carnitine	Acylcarnitines	HMDB00651	Level 2
C10:1	Decenoylcarnitine	Acylcarnitines		Level 2
C10:2	Decadienylcarnitine	Acylcarnitines		Level 2
C12	Dodecanoylcarnitine	Acylcarnitines	HMDB02250	Level 2
C12	Dodecanoyl-L-carnitine	Acylcarnitines	HMDB02250	Level 2
C12-DC	Dodecanedioylcarnitine	Acylcarnitines	HMDB13327	Level 2
C12:1	Dodecenoylcarnitine	Acylcarnitines		Level 2
C14	Myristylcarnitine	Acylcarnitines		Level 2
C14	Myristyl-L-carnitine	Acylcarnitines		Level 2
C14:1	cis-5-Tetradecenoylcarnitine	Acylcarnitines	HMDB02014	Level 2
C14:1	Tetradecenoylcarnitine	Acylcarnitines		Level 2
C14:1-OH	Hydroxytetradecenoylcarnitine	Acylcarnitines		Level 2
C14:2	Tetradecadienylcarnitine	Acylcarnitines		Level 2
C14:2-OH	Hydroxytetradecadienylcarnitine	Acylcarnitines		Level 2
C16	Palmitoylcarnitine	Acylcarnitines	HMDB00222	Level 2
C16	D-Palmitoylcarnitine	Acylcarnitines	HMDB00222	Level 2
C16	L-Palmitoylcarnitine	Acylcarnitines	HMDB00222	Level 2
C16-OH	Hydroxyhexadecanoylcarnitine	Acylcarnitines		Level 2
C16:1	trans-Hexadec-2-enoyl carnitine	Acylcarnitines	HMDB06317	Level 2
C16:1-OH	Hydroxyhexadecenoylcarnitine	Acylcarnitines		Level 2
C16:2	Hexadecadienylcarnitine	Acylcarnitines		Level 2
C16:2-OH	Hydroxyhexadecadienylcarnitine	Acylcarnitines		Level 2
C18	Stearoylcarnitine	Acylcarnitines	HMDB00848	Level 2
C18:1	Oleoylcarnitine	Acylcarnitines	HMDB06464	Level 2
C18:1	Elaidic carnitine	Acylcarnitines	HMDB06464	Level 2
C18:1-OH	Hydroxyoctadecenoylcarnitine	Acylcarnitines		Level 2
C18:2	Linoelaidylcarnitine	Acylcarnitines	HMDB06461	Level 2
Ala	Alanine	amino acids		Level 1
Ala	D-Alanine	amino acids	HMDB01310	Level 1
Ala	L-Alanine	amino acids	HMDB00161	Level 1
Ala	beta-Alanine	amino acids	HMDB00056	Level 1
Arg	Arginine	amino acids		Level 1
Arg	D-Arginine	amino acids	HMDB03416	Level 1
Arg	L-Arginine	amino acids	HMDB00517	Level 1
Asn	Asparagine	amino acids		Level 1

Metabolomics

Abbreviation	Name	Metabolite class	HMDB ID	MSI level
Asn	D-Asparagine	amino acids	HMDB33780	Level 1
Asn	L-Asparagine	amino acids	HMDB00168	Level 1
Asp	Aspartic acid	amino acids		Level 1
Asp	D-Aspartic acid	amino acids	HMDB06483	Level 1
Asp	L-Aspartic acid	amino acids	HMDB00191	Level 1
Cit	Citrulline	amino acids		Level 1
Cit	D-Citrulline	amino acids		Level 1
Cit	L-Citrulline	amino acids	HMDB00904	Level 1
Gln	Glutamine	amino acids		Level 1
Gln	D-Glutamine	amino acids	HMDB03423	Level 1
Gln	L-Glutamine	amino acids	HMDB00641	Level 1
Glu	Glutamic acid	amino acids		Level 1
Glu	D-Glutamic acid	amino acids	HMDB03339	Level 1
Glu	L-Glutamic acid	amino acids	HMDB00148	Level 1
Gly	Glycine	amino acids	HMDB00123	Level 1
His	Histidine	amino acids		Level 1
His	D-Histidine	amino acids		Level 1
His	L-Histidine	amino acids	HMDB00177	Level 1
Ile	Isoleucine	amino acids		Level 1
Ile	D-Isoleucine	amino acids		Level 1
Ile	L-Isoleucine	amino acids	HMDB00172	Level 1
Ile	Allo-D-Isoleucine	amino acids		Level 1
Ile	Allo-L-Isoleucine	amino acids	HMDB00557	Level 1
Leu	Leucine	amino acids		Level 2
Leu	D-Leucine	amino acids		Level 2
Leu	L-Leucine	amino acids	HMDB00687	Level 2
Leu	beta-Leucine	amino acids		Level 2
Leu	D-beta-Leucine	amino acids	HMDB03640	Level 2
Leu	L-beta-Leucine	amino acids	HMDB03640	Level 2
Lys	Lysine	amino acids		Level 2
Lys	D-Lysine	amino acids	HMDB03405	Level 2
Lys	L-Lysine	amino acids	HMDB00182	Level 2
Met	Methionine	amino acids		Level 1
Met	D-Methionine	amino acids		Level 1
Met	L-Methionine	amino acids	HMDB00696	Level 1
Orn	Ornithine	amino acids		Level 1
Orn	D-Ornithine	amino acids	HMDB03374	Level 1
Orn	L-Ornithine	amino acids	HMDB00214	Level 1
Phe	Phenylalanine	amino acids		Level 1
Phe	D-Phenylalanine	amino acids		Level 1
Phe	L-Phenylalanine	amino acids	HMDB00159	Level 1
Pro	Proline	amino acids		Level 1

Metabolomics

Abbreviation	Name	Metabolite class	HMDB ID	MSI level
Pro	D-Proline	amino acids	HMDB03411	Level 1
Pro	L-Proline	amino acids	HMDB00162	Level 1
Ser	Serine	amino acids		Level 1
Ser	D-Serine	amino acids	HMDB03406	Level 1
Ser	L-Serine	amino acids	HMDB00187	Level 1
Thr	Threonine	amino acids		Level 1
Thr	D-Allothreonine	amino acids		Level 1
Thr	L-Allothreonine	amino acids	HMDB04041	Level 1
Thr	L-Threonine	amino acids	HMDB00167	Level 1
Trp	Tryptophan	amino acids		Level 1
Trp	D-Tryptophan	amino acids	HMDB13609	Level 1
Trp	L-Tryptophan	amino acids	HMDB00929	Level 1
Tyr	Tyrosine	amino acids		Level 1
Tyr	D-Tyrosine	amino acids		Level 1
Tyr	L-Tyrosine	amino acids	HMDB00158	Level 1
Val	Valine	amino acids		Level 1
Val	D-Valine	amino acids		Level 1
Val	L-Valine	amino acids	HMDB00883	Level 1
ADMA	Asymmetric dimethylarginine	biogenic amines	HMDB01539	Level 1
Ac-Orn	Acetylornithine	biogenic amines	HMDB03357	Level 2
Carnosine	Carnosine	biogenic amines		Level 2
Carnosine	L-Carnosine	biogenic amines	HMDB00033	Level 2
Creatinine	Creatinine	biogenic amines	HMDB00562	Level 1
DOPA	Dihydroxyphenylalanine	biogenic amines	HMDB00609	Level 1
DOPA	Dihydroxyphenyl-D-alanine	biogenic amines		Level 1
DOPA	L-Dihydroxyphenylalanine	biogenic amines	HMDB00181	Level 1
Dopamine	Dopamine	biogenic amines	HMDB00073	Level 1
Histamine	Histamine	biogenic amines	HMDB00870	Level 2
Kynurenine	Kynurenine	biogenic amines		Level 2
Kynurenine	D-Kynurenine	biogenic amines		Level 2
Kynurenine	L-Kynurenine	biogenic amines	HMDB00684	Level 2
Met-SO	Methionine-Sulfoxide	biogenic amines		Level 2
Met-SO	L-Methionine sulfoxide	biogenic amines	HMDB02005	Level 2
Met-SO	L-Methionine-(R)-Sulfoxide	biogenic amines		Level 2
Met-SO	L-Methionine-(S)-Sulfoxide	biogenic amines		Level 2
Nitro-Tyr	2-Nitrotyrosine	biogenic amines		Level 2
Nitro-Tyr	3-Nitrotyrosine	biogenic amines	HMDB01904	Level 2
Nitro-Tyr	Nitro-D-tyrosine	biogenic amines		Level 2
Nitro-Tyr	Nitro-L-tyrosine	biogenic amines		Level 2
OH-Pro	4-Hydroxyproline	biogenic amines	HMDB00725	Level 2
OH-Pro	3-Hydroxy-L-proline	biogenic amines	HMDB02113	Level 2
OH-Pro	4-Hydroxy-L-proline	biogenic amines	HMDB00725	Level 2

Metabolomics

Abbreviation	Name	Metabolite class	HMDB ID	MSI level
OH-Pro	cis-4-Hydroxy-L-proline	biogenic amines	HMDB06055	Level 2
PEA	(1S)-Phenylethylamine	biogenic amines		Level 2
PEA	1-Phenylethylamine	biogenic amines	HMDB02017	Level 2
PEA	2-Phenylethylamine	biogenic amines	HMDB12275	Level 2
Putrescine	Putrescine	biogenic amines	HMDB01414	Level 1
SDMA	Symmetric dimethylarginine	biogenic amines	HMDB03334	Level 2
SDMA	L-Symmetric dimethylarginine	biogenic amines		Level 2
Serotonin	Serotonin	biogenic amines	HMDB00259	Level 1
Spermidine	Spermidine	biogenic amines	HMDB01257	Level 1
Spermine	Spermine	biogenic amines	HMDB01256	Level 1
Taurine	Taurine	biogenic amines	HMDB00251	Level 1
alpha-AAA	2-Aminoadipic acid	biogenic amines		Level 2
alpha-AAA	D-2-Aminoadipic acid	biogenic amines		Level 2
alpha-AAA	L-2-Aminoadipic acid	biogenic amines	HMDB00510	Level 2
lysoPC a C14:0	C14:0 a Lyso-phosphatidylcholine	Lyso-phosphatidylcholines		Level 2
lysoPC a C14:0	LysoPC(14:0)	Lyso-phosphatidylcholines	HMDB10379	Level 2
lysoPC a C14:0	C14:0 2a L-Lyso-phosphatidylcholine	Lyso-phosphatidylcholines		Level 2
lysoPC a C16:0	C16:0 a Lyso-phosphatidylcholine	Lyso-phosphatidylcholines		Level 2
lysoPC a C16:0	C16:0 a D-Lyso-phosphatidylcholine	Lyso-phosphatidylcholines		Level 2
lysoPC a C16:0	LysoPC(16:0)	Lyso-phosphatidylcholines	HMDB10382	Level 2
lysoPC a C16:0	C16:0 2a Lyso-phosphatidylcholine	Lyso-phosphatidylcholines		Level 2
lysoPC a C16:0	C16:0 2a L-Lyso-phosphatidylcholine	Lyso-phosphatidylcholines		Level 2
lysoPC a C16:1	C16:1;9Z-enoyl a Lyso-phosphatidylcholine	Lyso-phosphatidylcholines		Level 2
lysoPC a C16:1	C16:1,9E-enoyl a L-Lyso-phosphatidylcholine	Lyso-phosphatidylcholines		Level 2
lysoPC a C16:1	LysoPC(16:1(9Z))	Lyso-phosphatidylcholines	HMDB10383	Level 2
lysoPC a C17:0	C17:0 a Lyso-phosphatidylcholine	Lyso-phosphatidylcholines		Level 2
lysoPC a C17:0	LysoPC(17:0)	Lyso-phosphatidylcholines	HMDB12108	Level 2
lysoPC a C18:0	C18:0 a Lyso-phosphatidylcholine	Lyso-phosphatidylcholines		Level 2
lysoPC a C18:0	C18:0 a D-Lyso-phosphatidylcholine	Lyso-phosphatidylcholines		Level 2
lysoPC a C18:0	LysoPC(18:0)	Lyso-phosphatidylcholines	HMDB10384	Level 2
lysoPC a C18:0	C18:0 2a Lyso-phosphatidylcholine	Lyso-phosphatidylcholines		Level 2
lysoPC a C18:0	C18:0 2a D-Lyso-phosphatidylcholine	Lyso-phosphatidylcholines		Level 2
lysoPC a C18:0	LysoPC(0:0/18:0)	Lyso-phosphatidylcholines	HMDB11128	Level 2
lysoPC a C18:1	C18:1;9E-enoyl a Lyso-phosphatidylcholine	Lyso-phosphatidylcholines		Level 2

Abbreviation	Name	Metabolite class	HMDB ID	MSI level
lysoPC a C18:1	C18:1;9E-enoyl a L-Lyso-phosphatidylcholine	Lyso-phosphatidylcholines		Level 2
lysoPC a C18:1	C18:1;9E-enoyl 2a Lyso-phosphatidylcholine	Lyso-phosphatidylcholines		Level 2
lysoPC a C18:1	C18:1;9E-enoyl 2a L-Lyso-phosphatidylcholine	Lyso-phosphatidylcholines		Level 2
lysoPC a C18:1	C18:1;6Z-enoyl a L-Lyso-phosphatidylcholine	Lyso-phosphatidylcholines		Level 2
lysoPC a C18:1	C18:1;6Z-enoyl 2a L-Lyso-phosphatidylcholine	Lyso-phosphatidylcholines		Level 2
lysoPC a C18:1	C18:1;9Z-enoyl a Lyso-phosphatidylcholine	Lyso-phosphatidylcholines		Level 2
lysoPC a C18:1	LysoPC(18:1(9Z))	Lyso-phosphatidylcholines	HMDB02815	Level 2
lysoPC a C18:1	C18:1;9Z-enoyl 2a Lyso-phosphatidylcholine	Lyso-phosphatidylcholines		Level 2
lysoPC a C18:1	C18:1;9Z-enoyl 2a L-Lyso-phosphatidylcholine	Lyso-phosphatidylcholines		Level 2
lysoPC a C18:1	LysoPC(18:1(11Z))	Lyso-phosphatidylcholines	HMDB10385	Level 2
lysoPC a C18:2	C18:1;9Z,12Z-dienoyl a Lyso-phosphatidylcholine	Lyso-phosphatidylcholines		Level 2
lysoPC a C18:2	C18:1;2E,4E-dienoyl a L-Lyso-phosphatidylcholine	Lyso-phosphatidylcholines		Level 2
lysoPC a C18:2	LysoPC(18:2(9Z,12Z))	Lyso-phosphatidylcholines	HMDB10386	Level 2
lysoPC a C20:3	lysoPC a C20:3	Lyso-phosphatidylcholines		Level 2
lysoPC a C20:3	LysoPC(20:3(5Z,8Z,11Z))	Lyso-phosphatidylcholines	HMDB10393	Level 2
lysoPC a C20:3	LysoPC(20:3(8Z,11Z,14Z))	Lyso-phosphatidylcholines	HMDB10394	Level 2
lysoPC a C20:4	(5Z,8Z,11Z,14Z) a C20:4 Lyso-phosphatidylcholine	Lyso-phosphatidylcholines		Level 2
lysoPC a C20:4	LysoPC(20:4(5Z,8Z,11Z,14Z))	Lyso-phosphatidylcholines	HMDB10395	Level 2
lysoPC a C20:4	LysoPC(20:4(8Z,11Z,14Z,17Z))	Lyso-phosphatidylcholines	HMDB10396	Level 2
lysoPC a C24:0	C24:0 a Lyso-phosphatidylcholine	Lyso-phosphatidylcholines		Level 2
lysoPC a C24:0	LysoPC(24:0)	Lyso-phosphatidylcholines	HMDB10405	Level 2
lysoPC a C26:0	Lyso-phosphatidylcholine a C26:0	Lyso-phosphatidylcholines		Level 2
lysoPC a C26:1	Lyso-phosphatidylcholine a C26:1	Lyso-phosphatidylcholines		Level 2
lysoPC a C28:0	Lyso-phosphatidylcholine a C28:0	Lyso-phosphatidylcholines		Level 2
lysoPC a C28:1	Lyso-phosphatidylcholine a C28:1	Lyso-phosphatidylcholines		Level 2
PC aa C26:0	C(10:0/16:0) aa Phosphatidylcholine	Phosphatidylcholines		Level 2
PC aa C26:0	C(11:0/15:0) aa Phosphatidylcholine	Phosphatidylcholines		Level 2
PC aa C26:0	C(12:0/14:0) aa L-Phosphatidylcholine	Phosphatidylcholines		Level 2
PC aa C26:0	C(13:0/13:0) aa Phosphatidylcholine	Phosphatidylcholines		Level 2
PC aa C26:0	C(13:0/13:0) aa L- Phosphatidylcholine	Phosphatidylcholines		Level 2
PC aa C26:0	C(14:0/12:0) aa Phosphatidylcholine	Phosphatidylcholines		Level 2
PC aa C26:0	C(14:0/12:0) aa L-Phosphatidylcholine	Phosphatidylcholines		Level 2

Metabolomics

Abbreviation	Name	Metabolite class	HMDB ID	MSI level
PC aa C26:0	C(18:0/8:0) aa L-Phosphatidylcholine	Phosphatidylcholines		Level 2
PC aa C26:0	C(5:0/11:0) aa Phosphatidylcholine	Phosphatidylcholines		Level 2
PC aa C26:0	C(8:0/18:0) aa Phosphatidylcholine	Phosphatidylcholines		Level 2
PC aa C26:0	C(9:0/17:0) aa Phosphatidylcholine	Phosphatidylcholines		Level 2
PC aa C26:0	C(9:0/17:0) aa L-Phosphatidylcholine	Phosphatidylcholines		Level 2
PC aa C28:1	C(10:0/18:1;9Z-enoyl) aa L-Phosphatidylcholine	Phosphatidylcholines		Level 2
PC aa C30:0	C(12:0/18:0) aa Phosphatidylcholine	Phosphatidylcholines		Level 2
PC aa C30:0	C(12:0/18:0) aa L-Phosphatidylcholine	Phosphatidylcholines		Level 2
PC aa C30:0	C(10:0/20:0) aa L-Phosphatidylcholine	Phosphatidylcholines		Level 2
PC aa C30:0	C(11:0/19:0) aa L-Phosphatidylcholine	Phosphatidylcholines		Level 2
PC aa C30:0	C(13:0/17:0) aa L-Phosphatidylcholine	Phosphatidylcholines		Level 2
PC aa C30:0	C(14:0/16:0) aa Phosphatidylcholine	Phosphatidylcholines		Level 2
PC aa C30:0	PC(16:0/14:0)	Phosphatidylcholines	HMDB07965	Level 2
PC aa C30:0	C(15:0/15:0) aa Phosphatidylcholine	Phosphatidylcholines		Level 2
PC aa C30:0	PC(15:0/15:0)	Phosphatidylcholines	HMDB07934	Level 2
PC aa C30:0	C(16:0/14:0) aa Phosphatidylcholine	Phosphatidylcholines		Level 2
PC aa C30:0	C(16:0/14:0) aa D-Phosphatidylcholine	Phosphatidylcholines		Level 2
PC aa C30:0	PC(14:0/16:0)	Phosphatidylcholines	HMDB07869	Level 2
PC aa C30:0	C(17:0/13:0) aa L-Phosphatidylcholine	Phosphatidylcholines		Level 2
PC aa C30:0	C(18:0/12:0) aa Phosphatidylcholine	Phosphatidylcholines		Level 2
PC aa C30:0	C(18:0/12:0) aa L-Phosphatidylcholine	Phosphatidylcholines		Level 2
PC aa C30:0	C(19:0/11:0) aa L-Phosphatidylcholine	Phosphatidylcholines		Level 2
PC aa C30:0	C(20:0/10:0) aa L-Phosphatidylcholine	Phosphatidylcholines		Level 2
PC aa C30:0	C(21:0/9:0) aa L-Phosphatidylcholine	Phosphatidylcholines		Level 2
PC aa C30:2	Phosphatidylcholine with diacyl residue sum C30:2	Phosphatidylcholines		Level 2
PC aa C32:0	C(18:0/14:0) aa Phosphatidylcholine	Phosphatidylcholines		Level 2
PC aa C32:0	PC(14:0/18:0)	Phosphatidylcholines	HMDB07871	Level 2
PC aa C32:0	C(10:0/22:0) aa L-Phosphatidylcholine	Phosphatidylcholines		Level 2
PC aa C32:0	C(11:0/21:0) aa L-Phosphatidylcholine	Phosphatidylcholines		Level 2
PC aa C32:0	C(12:0/20:0) aa Phosphatidylcholine	Phosphatidylcholines		Level 2
PC aa C32:0	C(12:0/20:0) aa L-Phosphatidylcholine	Phosphatidylcholines		Level 2
PC aa C32:0	C(13:0/19:0) aa L-Phosphatidylcholine	Phosphatidylcholines		Level 2
PC aa C32:0	C(14:0/18:0) aa Phosphatidylcholine	Phosphatidylcholines		Level 2
PC aa C32:0	PC(18:0/14:0)	Phosphatidylcholines	HMDB08031	Level 2
PC aa C32:0	C(15:0/17:0) aa Phosphatidylcholine	Phosphatidylcholines		Level 2
PC aa C32:0	C(15:0/17:0) aa L-Phosphatidylcholine	Phosphatidylcholines		Level 2
PC aa C32:0	C(16:0/16:0) aa Phosphatidylcholine	Phosphatidylcholines		Level 2
PC aa C32:0	PC(16:0/16:0)	Phosphatidylcholines	HMDB00564	Level 2
PC aa C32:0	C(17:0/15:0) aa Phosphatidylcholine	Phosphatidylcholines		Level 2
PC aa C32:0	C(17:0/15:0) aa L-Phosphatidylcholine	Phosphatidylcholines		Level 2
PC aa C32:0	C(19:0/13:0) aa Phosphatidylcholine	Phosphatidylcholines		Level 2

Metabolomics

Abbreviation	Name	Metabolite class	HMDB ID	MSI level
PC aa C32:0	C(19:0/13:0) aa L-Phosphatidylcholine	Phosphatidylcholines		Level 2
PC aa C32:0	C(20:0/12:0) aa Phosphatidylcholine	Phosphatidylcholines		Level 2
PC aa C32:0	C(20:0/12:0) aa L-Phosphatidylcholine	Phosphatidylcholines		Level 2
PC aa C32:0	C(21:0/11:0) aa L-Phosphatidylcholine	Phosphatidylcholines		Level 2
PC aa C32:0	C(22:0/10:0) aa Phosphatidylcholine	Phosphatidylcholines		Level 2
PC aa C32:0	C(22:0/10:0) aa L-Phosphatidylcholine	Phosphatidylcholines		Level 2
PC aa C32:0	C(23:0/9:0) aa L-Phosphatidylcholine	Phosphatidylcholines		Level 2
PC aa C32:1	C(18:1;9E-enoyl/14:0) aa Phosphatidylcholine	Phosphatidylcholines		Level 2
PC aa C32:1	PC(14:0/18:1(11Z))	Phosphatidylcholines	HMDB07872	Level 2
PC aa C32:1	C(14:0/18:1;9Z-enoyl)) aa Phosphatidylcholine	Phosphatidylcholines		Level 2
PC aa C32:1	PC(18:1(9Z)/14:0)	Phosphatidylcholines	HMDB08097	Level 2
PC aa C32:1	C(16:1;9Z-enoyl)/16:0) aa Phosphatidylcholine	Phosphatidylcholines		Level 2
PC aa C32:1	PC(16:0/16:1(9Z))	Phosphatidylcholines	HMDB07969	Level 2
PC aa C32:1	C(18:1;9Z-enoyl)/14:0) aa Phosphatidylcholine	Phosphatidylcholines		Level 2
PC aa C32:1	PC(14:0/18:1(9Z))	Phosphatidylcholines	HMDB07873	Level 2
PC aa C32:2	C(16:1;9E-enoyl/16:1;9E-enoyl) aa Phosphatidylcholine	Phosphatidylcholines		Level 2
PC aa C32:2	C(16:1;9E-enoyl/16:1;9E-enoyl) aa L-Phosphatidylcholine	Phosphatidylcholines		Level 2
PC aa C32:2	C(16:1;9Z-enoyl/16:1;9Z-enoyl) aa Phosphatidylcholine	Phosphatidylcholines		Level 2
PC aa C32:2	PC(16:1(9Z)/16:1(9Z))	Phosphatidylcholines	HMDB08002	Level 2
PC aa C32:2	C(18:2;2E,4E-dienoyl/14:0) aa Phosphatidylcholine	Phosphatidylcholines		Level 2
PC aa C32:2	C(18:2;11Z,14Z-dienoyl/14:0) aa L-Phosphatidylcholine	Phosphatidylcholines		Level 2
PC aa C32:2	PC(14:0/18:2(9Z,12Z))	Phosphatidylcholines	HMDB07874	Level 2
PC aa C32:3	C(18:3;9Z,12Z,15Z-trienoyl/14:0) aa Phosphatidylcholine	Phosphatidylcholines		Level 2
PC aa C32:3	PC(14:0/18:3(9Z,12Z,15Z))	Phosphatidylcholines	HMDB07876	Level 2
PC aa C34:1	PC(18:1(9Z)/16:0)	Phosphatidylcholines	HMDB08100	Level 2
PC aa C34:1	C(16:0/18:1;9Z-enoyl) aa L-Phosphatidylcholine	Phosphatidylcholines		Level 2
PC aa C34:1	C(16:0/18:1;9Z-enoyl) aa Phosphatidylcholine	Phosphatidylcholines		Level 2
PC aa C34:1	PC(18:0/16:1(9Z))	Phosphatidylcholines	HMDB08035	Level 2
PC aa C34:1	C(18:0/16:1;7Z-enoyl) aa Phosphatidylcholine	Phosphatidylcholines		Level 2
PC aa C34:1	PC(16:1(9Z)/18:0)	Phosphatidylcholines	HMDB08003	Level 2
PC aa C34:1	C(18:1;11E-enoyl/16:0) aa L-Phosphatidylcholine	Phosphatidylcholines		Level 2
PC aa C34:1	C(18:1;11Z-enoyl/16:0) aa Phosphatidylcholine	Phosphatidylcholines		Level 2
PC aa C34:1	PC(16:0/18:1(11Z))	Phosphatidylcholines	HMDB07971	Level 2
PC aa C34:1	C(18:1;6Z-enoyl/16:0) aa Phosphatidylcholine	Phosphatidylcholines		Level 2
PC aa C34:1	C(18:1;6E-enoyl/16:0) aa L-Phosphatidylcholine	Phosphatidylcholines		Level 2
PC aa C34:1	C(18:1;6Z-enoyl/16:0)) aa L-Phosphatidylcholine	Phosphatidylcholines		Level 2
PC aa C34:1	PC(16:0/18:1(9Z))	Phosphatidylcholines	HMDB07972	Level 2
PC aa C34:1	C(18:1;9E-enoyl/16:0)) aa D-	Phosphatidylcholines		Level 2

Abbreviation	Name	Metabolite class	HMDB ID	MSI level
	Phosphatidylcholine			
PC aa C34:1	C(18:1;9E-enoyl/16:0)) aa L-Phosphatidylcholine	Phosphatidylcholines		Level 2
PC aa C34:1	C(18:1;9Z-enoyl/16:0)) aa Phosphatidylcholine	Phosphatidylcholines		Level 2
PC aa C34:1	C(18:1;9Z-enoyl/16:0) aa L-Phosphatidylcholine	Phosphatidylcholines		Level 2
PC aa C34:2	C(16:0/18:2;2E,4E-dienoyl) aa L-Phosphatidylcholine	Phosphatidylcholines		Level 2
PC aa C34:2	C(16:0/18:2;6Z,9Z-dienoyl) aa L-Phosphatidylcholine	Phosphatidylcholines		Level 2
PC aa C34:2	C(16:0/18:2;9Z,12Z-dienoyl) aa Phosphatidylcholine	Phosphatidylcholines		Level 2
PC aa C34:2	PC(18:2(9Z,12Z)/16:0)	Phosphatidylcholines	HMDB08133	Level 2
PC aa C34:2	C(16:1;2E,4E-dienoyl/18:0) aa L-Phosphatidylcholine	Phosphatidylcholines		Level 2
PC aa C34:2	C(16:1;3E-enoyl/18:1;9Z-enoyl) aa Phosphatidylcholine	Phosphatidylcholines		Level 2
PC aa C34:2	PC(18:1(9Z)/16:1(9Z))	Phosphatidylcholines	HMDB08101	Level 2
PC aa C34:2	C(17:1;10E-enoyl/17:1;10E-enoyl) aa Phosphatidylcholine	Phosphatidylcholines		Level 2
PC aa C34:2	C(17:1;10E-enoyl/17:1;10Z-enoyl) aa L-Phosphatidylcholine	Phosphatidylcholines		Level 2
PC aa C34:2	C(17:1;10E-enoyl/17:1;9Z-enoyl) aa L-Phosphatidylcholine	Phosphatidylcholines		Level 2
PC aa C34:2	PC(16:1(9Z)/18:1(11Z))	Phosphatidylcholines	HMDB08004	Level 2
PC aa C34:2	C(18:1;9Z-enoyl/16:1;2Z-enoyl) aa L-Phosphatidylcholine	Phosphatidylcholines		Level 2
PC aa C34:2	C(18:1;9Z-enoyl/16:1;7Z-enoyl) aa Phosphatidylcholine	Phosphatidylcholines		Level 2
PC aa C34:2	C(18:1;9Z-enoyl/16:1;9Z-enoyl) aa Phosphatidylcholine	Phosphatidylcholines		Level 2
PC aa C34:2	PC(16:1(9Z)/18:1(9Z))	Phosphatidylcholines	HMDB08005	Level 2
PC aa C34:2	C(18:2;10E,12Z-dienoyl/16:0) aa L-Phosphatidylcholine	Phosphatidylcholines		Level 2
PC aa C34:2	C(18:2;11Z,13Z-dienoyl/16:0) aa L-Phosphatidylcholine	Phosphatidylcholines		Level 2
PC aa C34:2	C(18:2;2E,4E-dienoyl/16:0) aa L-Phosphatidylcholine	Phosphatidylcholines		Level 2
PC aa C34:2	C(18:2;2Z,4Z-dienoyl/16:0) aa L-Phosphatidylcholine	Phosphatidylcholines		Level 2
PC aa C34:2	C(18:2;6Z,9Z-dienoyl/16:0) aa L-Phosphatidylcholine	Phosphatidylcholines		Level 2
PC aa C34:2	C(18:2;9E,11E-dienoyl/16:0) aa L-Phosphatidylcholine	Phosphatidylcholines		Level 2
PC aa C34:2	C(18:2;9E,11Z-dienoyl/16:0) aa L-Phosphatidylcholine	Phosphatidylcholines		Level 2
PC aa C34:2	PC(16:0/18:2(9Z,12Z))	Phosphatidylcholines	HMDB07973	Level 2
PC aa C34:2	C(18:2;9E,12E-dienoyl/16:0) aa L-Phosphatidylcholine	Phosphatidylcholines		Level 2
PC aa C34:2	C(18:2;9Z,12Z-dienoyl/16:0) aa Phosphatidylcholine	Phosphatidylcholines		Level 2
PC aa C34:2	C(18:2;9Z,12Z-dienoyl/16:0) aa D-Phosphatidylcholine	Phosphatidylcholines		Level 2
PC aa C34:2	C(18:2;9Z,12Z-dienoyl/16:0) aa L-Phosphatidylcholine	Phosphatidylcholines		Level 2
PC aa C34:3	C(18:2;9Z,12Z-dienoyl/16:1;7Z-enoyl) aa Phosphatidylcholine	Phosphatidylcholines		Level 2

Metabolomics

Abbreviation	Name	Metabolite class	HMDB ID	MSI level
PC aa C34:3	C(18:2;9Z,12Z-dienoyl/16:1;9Z-enoyl) aa Phosphatidylcholine	Phosphatidylcholines		Level 2
PC aa C34:3	PC(16:1(9Z)/18:2(9Z,12Z))	Phosphatidylcholines	HMDB08006	Level 2
PC aa C34:3	C(18:3;5E,9Z,12Z-trienoyl/16:0) aa Phosphatidylcholine	Phosphatidylcholines		Level 2
PC aa C34:3	C(18:3;6Z,9Z,12Z-trienoyl/16:0) aa Phosphatidylcholine	Phosphatidylcholines		Level 2
PC aa C34:3	PC(16:0/18:3(6Z,9Z,12Z))	Phosphatidylcholines	HMDB07974	Level 2
PC aa C34:3	C(18:3;9E,12E,15E-trienoyl/16:0) aa Phosphatidylcholine	Phosphatidylcholines		Level 2
PC aa C34:3	PC(16:0/18:3(9Z,12Z,15Z))	Phosphatidylcholines	HMDB07975	Level 2
PC aa C34:3	C(18:3;9Z,12Z,15Z-trienoyl/16:0) aa Phosphatidylcholine	Phosphatidylcholines		Level 2
PC aa C34:3	C(19:3;9Z,12Z,15Z-trienoyl/15:0) aa Phosphatidylcholine	Phosphatidylcholines		Level 2
PC aa C34:4	C(16:0/18:4;9Z,11E,13E,15Z-tetraenoyl) aa L-Phosphatidylcholine	Phosphatidylcholines		Level 2
PC aa C34:4	C(16:2;5Z,8Z-dienoyl/18:2;9Z,12Z-dienoyl) aa Phosphatidylcholine	Phosphatidylcholines		Level 2
PC aa C34:4	C(17:2;9Z,11Z-dienoyl/17:2;9Z,11Z-dienoyll) aa L-Phosphatidylcholine	Phosphatidylcholines		Level 2
PC aa C34:4	C(18:2;2E,4E-dienoyl/16:2;2E,4E-dienoyl) aa L-Phosphatidylcholine	Phosphatidylcholines		Level 2
PC aa C34:4	PC(16:0/18:4(6Z,9Z,12Z,15Z))	Phosphatidylcholines	HMDB07976	Level 2
PC aa C34:4	C(18:4;9E,11E,13E,15E-tetraenoyl/16:0) aa L-Phosphatidylcholine	Phosphatidylcholines		Level 2
PC aa C34:4	C(18:4;9E,11E,13E,15E-tetraenoyl/16:0) aa Phosphatidylcholine	Phosphatidylcholines		Level 2
PC aa C34:4	C(18:4;9Z,11E,13E,15Z-tetraenoyl/16:0) aa L-Phosphatidylcholine	Phosphatidylcholines		Level 2
PC aa C34:4	C(18:4;9Z,11E,13E,15Z-tetraenoyl/16:0) aa Phosphatidylcholine	Phosphatidylcholines		Level 2
PC aa C34:4	C(18:4;9Z,11Z,13Z,15Z-tetraenoyl/16:0) aa L-Phosphatidylcholine	Phosphatidylcholines		Level 2
PC aa C34:4	PC(14:0/20:4(5Z,8Z,11Z,14Z))	Phosphatidylcholines	HMDB07883	Level 2
PC aa C34:4	C(20:4;5Z,8Z,11Z,14Z-tetraenoyl/14:0) aa Phosphatidylcholine	Phosphatidylcholines		Level 2
PC aa C36:0	C(11:0/25:0) aa Phosphatidylcholine	Phosphatidylcholines		Level 2
PC aa C36:0	C(12:0/24:0) aa Phosphatidylcholine	Phosphatidylcholines		Level 2
PC aa C36:0	C(13:0/23:0) aa Phosphatidylcholine	Phosphatidylcholines		Level 2
PC aa C36:0	PC(22:0/14:0)	Phosphatidylcholines	HMDB08525	Level 2
PC aa C36:0	C(15:0/21:0) aa Phosphatidylcholine	Phosphatidylcholines		Level 2
PC aa C36:0	PC(20:0/16:0)	Phosphatidylcholines	HMDB08265	Level 2
PC aa C36:0	C(17:0/19:0) aa Phosphatidylcholine	Phosphatidylcholines		Level 2
PC aa C36:0	PC(18:0/18:0)	Phosphatidylcholines	HMDB08036	Level 2
PC aa C36:0	C(18:0/18:0) aa L-Phosphatidylcholine	Phosphatidylcholines		Level 2
PC aa C36:0	C(18:0/18:0) aa Phosphatidylcholine	Phosphatidylcholines		Level 2
PC aa C36:0	C(19:0/17:0) aa Phosphatidylcholine	Phosphatidylcholines		Level 2
PC aa C36:0	PC(16:0/20:0)	Phosphatidylcholines	HMDB07977	Level 2
PC aa C36:0	C(20:0/16:0) aa Phosphatidylcholine	Phosphatidylcholines		Level 2
PC aa C36:0	C(21:0/15:0) aa L-Phosphatidylcholine	Phosphatidylcholines		Level 2
PC aa C36:0	PC(14:0/22:0)	Phosphatidylcholines	HMDB07886	Level 2

Metabolomics

Abbreviation	Name	Metabolite class	HMDB ID	MSI level
PC aa C36:0	C(23:0/13:0) aa L-Phosphatidylcholine	Phosphatidylcholines		Level 2
PC aa C36:0	C(24:0/12:0) aa L-Phosphatidylcholine	Phosphatidylcholines		Level 2
PC aa C36:0	C(24:0/12:0) aa Phosphatidylcholine	Phosphatidylcholines		Level 2
PC aa C36:0	C(25:0/11:0) aa L-Phosphatidylcholine	Phosphatidylcholines		Level 2
PC aa C36:0	C(26:0/10:0) aa Phosphatidylcholine	Phosphatidylcholines		Level 2
PC aa C36:1	C(16:0/20:1;11Z-enoyl) aa Phosphatidylcholine	Phosphatidylcholines		Level 2
PC aa C36:1	C(18:0/18:1;9E-enoyl) aa Phosphatidylcholine	Phosphatidylcholines		Level 2
PC aa C36:1	PC(18:1(9Z)/18:0)	Phosphatidylcholines	HMDB08102	Level 2
PC aa C36:1	C(18:0/18:1;9Z-enoyl) aa Phosphatidylcholine	Phosphatidylcholines		Level 2
PC aa C36:1	PC(18:0/18:1(11Z))	Phosphatidylcholines	HMDB08037	Level 2
PC aa C36:1	PC(18:1(11Z)/18:0)	Phosphatidylcholines	HMDB08069	Level 2
PC aa C36:1	C(18:1;11Z-enoyl/18:0) aa Phosphatidylcholine	Phosphatidylcholines		Level 2
PC aa C36:1	C(18:1;12Z-enoyl/18:0) aa L-Phosphatidylcholine	Phosphatidylcholines		Level 2
PC aa C36:1	C(18:1;12Z-enoyl/18:0) aa Phosphatidylcholine	Phosphatidylcholines		Level 2
PC aa C36:1	C(18:1;13Z-enoyl/18:0) aa L-Phosphatidylcholine	Phosphatidylcholines		Level 2
PC aa C36:1	C(18:1;16Z-enoyl/18:0) aa L-Phosphatidylcholine	Phosphatidylcholines		Level 2
PC aa C36:1	C(18:1;16Z-enoyl/18:0) aa Phosphatidylcholine	Phosphatidylcholines		Level 2
PC aa C36:1	C(18:1;6Z-enoyl/18:0) aa L-Phosphatidylcholine	Phosphatidylcholines		Level 2
PC aa C36:1	C(18:1;6Z-enoyl/18:0) aa Phosphatidylcholine	Phosphatidylcholines		Level 2
PC aa C36:1	C(18:1;7Z-enoyl/18:0) aa L-Phosphatidylcholine	Phosphatidylcholines		Level 2
PC aa C36:1	C(18:1;9E-enoyl/18:0) aa L-Phosphatidylcholine	Phosphatidylcholines		Level 2
PC aa C36:1	C(18:1;9E-enoyl/18:0) aa Phosphatidylcholine	Phosphatidylcholines		Level 2
PC aa C36:1	C(18:1;9Z-enoyl/18:0) aa D-Phosphatidylcholine	Phosphatidylcholines		Level 2
PC aa C36:1	PC(18:0/18:1(9Z))	Phosphatidylcholines	HMDB08038	Level 2
PC aa C36:1	C(18:1;9Z-enoyl/18:0) aa Phosphatidylcholine	Phosphatidylcholines		Level 2
PC aa C36:1	C(20:1;11Z-enoyl/16:0) aa Phosphatidylcholine	Phosphatidylcholines		Level 2
PC aa C36:2	PC(18:2(9Z,12Z)/18:0)	Phosphatidylcholines	HMDB08135	Level 2
PC aa C36:2	C(18:0/18:2;9Z,12Z-dienoyl) aa Phosphatidylcholine	Phosphatidylcholines		Level 2
PC aa C36:2	C(18:1;10E-enoyl/18:1;10E-enoyl) aa Phosphatidylcholine	Phosphatidylcholines		Level 2
PC aa C36:2	C(18:1;10Z-enoyl/18:1;10Z-enoyl) aa L-Phosphatidylcholine	Phosphatidylcholines		Level 2
PC aa C36:2	C(18:1;11E-enoyl/18:1;11E-enoyl) aa L-Phosphatidylcholine	Phosphatidylcholines		Level 2
PC aa C36:2	C(18:1;11E-enoyl/18:1;11E-enoyl) aa Phosphatidylcholine	Phosphatidylcholines		Level 2
PC aa C36:2	PC(18:1(11Z)/18:1(11Z))	Phosphatidylcholines	HMDB08070	Level 2
PC aa C36:2	C(18:1;11Z-enoyl/18:1;11Z-enoyl) aa Phosphatidylcholine	Phosphatidylcholines		Level 2
PC aa C36:2	C(18:1;12E-enoyl/18:1;12E-enoyl) aa Phosphatidylcholine	Phosphatidylcholines		Level 2
PC aa C36:2	C(18:1;12Z-enoyl/18:1;12Z-enoyl) aa L-Phosphatidylcholine	Phosphatidylcholines		Level 2

Metabolomics

Abbreviation	Name	Metabolite class	HMDB ID	MSI level
PC aa C36:2	C(18:1;13E-enoyl/18:1;13E-enoyl) aa Phosphatidylcholine	Phosphatidylcholines		Level 2
PC aa C36:2	C(18:1;13Z-enoyl/18:1;13E-enoyl) aa L-Phosphatidylcholine	Phosphatidylcholines		Level 2
PC aa C36:2	C(18:1;14E-enoyl/18:1;14E-enoyl) aa Phosphatidylcholine	Phosphatidylcholines		Level 2
PC aa C36:2	C(18:1;14Z-enoyl/18:1;14Z-enoyl) aa L-Phosphatidylcholine	Phosphatidylcholines		Level 2
PC aa C36:2	C(18:1;15E-enoyl/18:1;15E-enoyl) aa Phosphatidylcholine	Phosphatidylcholines		Level 2
PC aa C36:2	C(18:1;15Z-enoyl/18:1;15Z-enoyl) aa L-Phosphatidylcholine	Phosphatidylcholines		Level 2
PC aa C36:2	C(18:1;16E-enoyl/18:1;16E-enoyl) aa Phosphatidylcholine	Phosphatidylcholines		Level 2
PC aa C36:2	C(18:1;16Z-enoyl/18:1;16Z-enoyl) aa L-Phosphatidylcholine	Phosphatidylcholines		Level 2
PC aa C36:2	C(18:1;17-enoyl/18:1;17-enoyl) aa L-Phosphatidylcholine	Phosphatidylcholines		Level 2
PC aa C36:2	C(18:1;2E-enoyl/18:1;2E-enoyl) aa Phosphatidylcholine	Phosphatidylcholines		Level 2
PC aa C36:2	C(18:1;2Z-enoyl/18:1;2Z-enoyl) aa L-Phosphatidylcholine	Phosphatidylcholines		Level 2
PC aa C36:2	C(18:1;3E-enoyl/18:1;3E-enoyl) aa Phosphatidylcholine	Phosphatidylcholines		Level 2
PC aa C36:2	C(18:1;3Z-enoyl/18:1;3Z-enoyl) aa L-Phosphatidylcholine	Phosphatidylcholines		Level 2
PC aa C36:2	C(18:1;4E-enoyl/18:1;4E-enoyl) aa Phosphatidylcholine	Phosphatidylcholines		Level 2
PC aa C36:2	C(18:1;4Z-enoyl/18:1;4Z-enoyl) aa Phosphatidylcholine	Phosphatidylcholines		Level 2
PC aa C36:2	C(18:1;5E-enoyl/18:1;5E-enoyl) aa Phosphatidylcholine	Phosphatidylcholines		Level 2
PC aa C36:2	C(18:1;5Z-enoyl/18:1;5Z-enoyl) aa L-Phosphatidylcholine	Phosphatidylcholines		Level 2
PC aa C36:2	C(18:1;6E-enoyl/18:1;6E-enoyl) aa L-Phosphatidylcholine	Phosphatidylcholines		Level 2
PC aa C36:2	C(18:1;6E-enoyl/18:1;6E-enoyl) aa Phosphatidylcholine	Phosphatidylcholines		Level 2
PC aa C36:2	C(18:1;6Z-enoyl/18:1;6Z-enoyl) aa L-Phosphatidylcholine	Phosphatidylcholines		Level 2
PC aa C36:2	C(18:1;6Z-enoyl/18:1;6Z-enoyl) aa Phosphatidylcholine	Phosphatidylcholines		Level 2
PC aa C36:2	C(18:1;7E-enoyl/18:1;7E-enoyl) aa Phosphatidylcholine	Phosphatidylcholines		Level 2
PC aa C36:2	C(18:1;7Z-enoyl/18:1;7Z-enoyl) aa L-Phosphatidylcholine	Phosphatidylcholines		Level 2
PC aa C36:2	C(18:1;8E-enoyl/18:1;8E-enoyl) aa Phosphatidylcholine	Phosphatidylcholines		Level 2
PC aa C36:2	C(18:1;8Z-enoyl/18:1;8Z-enoyl) aa L-Phosphatidylcholine	Phosphatidylcholines		Level 2
PC aa C36:2	C(18:1;9E-enoyl/18:1;9E-enoyl) aa L-Phosphatidylcholine	Phosphatidylcholines		Level 2
PC aa C36:2	C(18:1;9E-enoyl/18:1;9E-enoyl) aa Phosphatidylcholine	Phosphatidylcholines		Level 2
PC aa C36:2	C(18:1;9Z-enoyl/18:1;9Z-enoyl) aa D-Phosphatidylcholine	Phosphatidylcholines		Level 2
PC aa C36:2	PC(18:1(9Z)/18:1(9Z))	Phosphatidylcholines	HMDB00593	Level 2
PC aa C36:2	C(18:2;10Z,12Z-dienoyl/18:0) aa L-Phosphatidylcholine	Phosphatidylcholines		Level 2
PC aa C36:2	C(18:2;2E,4E-dienoyl/18:0) aa L-	Phosphatidylcholines		Level 2

Metabolomics

Abbreviation	Name	Metabolite class	HMDB ID	MSI level
	Phosphatidylcholine			
PC aa C36:2	C(18:2;6Z,9Z-dienoyl/18:0) aa L-Phosphatidylcholine	Phosphatidylcholines		Level 2
PC aa C36:2	C(18:2;6Z,9Z-dienoyl/18:0) aa Phosphatidylcholine	Phosphatidylcholines		Level 2
PC aa C36:2	PC(18:0/18:2(9Z,12Z))	Phosphatidylcholines	HMDB08039	Level 2
PC aa C36:2	C(18:2;9Z,12Z-dienoyl/18:0) aa Phosphatidylcholine	Phosphatidylcholines		Level 2
PC aa C36:2	C(20:2;11E,14E-enoyl/16:0) aa Phosphatidylcholine	Phosphatidylcholines		Level 2
PC aa C36:3	PC(18:0/18:3(6Z,9Z,12Z))	Phosphatidylcholines	HMDB08040	Level 2
PC aa C36:3	C(18:3;9Z,12Z,15Z-trienoyl/18:0) aa Phosphatidylcholine	Phosphatidylcholines		Level 2
PC aa C36:3	C(20:3;11E,14E,17E-trienoyl/16:0) aa Phosphatidylcholine	Phosphatidylcholines		Level 2
PC aa C36:3	C(20:3;5E,8E,11E-trienoyl/16:0) aa Phosphatidylcholine	Phosphatidylcholines		Level 2
PC aa C36:3	PC(16:0/20:3(5Z,8Z,11Z))	Phosphatidylcholines	HMDB07980	Level 2
PC aa C36:3	C(20:3;5Z,8Z,11Z-trienoyl/16:0) aa Phosphatidylcholine	Phosphatidylcholines		Level 2
PC aa C36:3	C(20:3;8E,11E,14E-trienoyl/16:0) aa D-Phosphatidylcholine	Phosphatidylcholines		Level 2
PC aa C36:3	C(20:3;8E,11E,14E-trienoyl/16:0) aa L-Phosphatidylcholine	Phosphatidylcholines		Level 2
PC aa C36:3	C(20:3;8E,11E,14E-trienoyl/16:0) aa Phosphatidylcholine	Phosphatidylcholines		Level 2
PC aa C36:3	PC(16:0/20:3(8Z,11Z,14Z))	Phosphatidylcholines	HMDB07981	Level 2
PC aa C36:3	C(20:3;8Z,11Z,14Z-trienoyl/16:0) aa Phosphatidylcholine	Phosphatidylcholines		Level 2
PC aa C36:3	C18:2;6Z,9Z-dienoyl/18:1;9Z-enoyl) aa L-Phosphatidylcholine	Phosphatidylcholines		Level 2
PC aa C36:3	C18:2;6Z,9Z-dienoyl/18:1;9Z-enoyl) aa Phosphatidylcholine	Phosphatidylcholines		Level 2
PC aa C36:3	PC(18:1(9Z)/18:2(9Z,12Z))	Phosphatidylcholines	HMDB08105	Level 2
PC aa C36:3	C18:2;9Z,12Z-dienoyl/18:1;9Z-enoyl) aa Phosphatidylcholine	Phosphatidylcholines		Level 2
PC aa C36:4	C(16:0/20:4;Z,8E,11E,14E,17E-tetraenoyl) aa L-Phosphatidylcholine	Phosphatidylcholines		Level 2
PC aa C36:4	PC(20:4(5Z,8Z,11Z,14Z)/16:0)	Phosphatidylcholines	HMDB08429	Level 2
PC aa C36:4	C(16:0/20:4;Z,8Z,11Z,14Z-tetraenoyl) aa Phosphatidylcholine	Phosphatidylcholines		Level 2
PC aa C36:4	PC(18:3(6Z,9Z,12Z)/18:1(9Z))	Phosphatidylcholines	HMDB08170	Level 2
PC aa C36:4	PC(18:3(9Z,12Z,15Z)/18:1(9Z))	Phosphatidylcholines	HMDB08203	Level 2
PC aa C36:4	C(18:2;15E,17-dienoyl/18:2;15E,17-dienoyl) aa Phosphatidylcholine	Phosphatidylcholines		Level 2
PC aa C36:4	C(18:2;2E,4E-dienoyl/18:2;2E,17-dienoyl) aa D-Phosphatidylcholine	Phosphatidylcholines		Level 2
PC aa C36:4	C(18:2;2E,4E-dienoyl/18:2;2E,4E-dienoyl) aa D-Phosphatidylcholine	Phosphatidylcholines		Level 2
PC aa C36:4	C(18:2;2E,4E-dienoyl/18:2;2E,4E-dienoyl) aa L-Phosphatidylcholine	Phosphatidylcholines		Level 2
PC aa C36:4	C(18:2;2E,4E-dienoyl/18:2;2E,4E-dienoyl) aa Phosphatidylcholine	Phosphatidylcholines		Level 2
PC aa C36:4	C(18:2;2Z,4Z-dienoyl/18:2;2Z,4Z-dienoyl) aa L-Phosphatidylcholine	Phosphatidylcholines		Level 2
PC aa C36:4	C(18:2;5Z,8Z-dienoyl/18:2;5Z,9Z-dienoyl) aa Phosphatidylcholine	Phosphatidylcholines		Level 2

Metabolomics

Abbreviation	Name	Metabolite class	HMDB ID	MSI level
PC aa C36:4	C(18:2;6Z,9Z-dienoyl/18:2;6Z,9Z-dienoyl) aa L-Phosphatidylcholine	Phosphatidylcholines		Level 2
PC aa C36:4	C(18:2;6Z,9Z-dienoyl/18:2;6Z,9Z-dienoyl) aa Phosphatidylcholine	Phosphatidylcholines		Level 2
PC aa C36:4	C(18:2;9E,12Z-dienoyl/18:2;9E,12Z-dienoyl) aa Phosphatidylcholine	Phosphatidylcholines		Level 2
PC aa C36:4	C(18:2;9Z,11Z-dienoyl/18:2;9Z,11Z-dienoyl) aa L-Phosphatidylcholine	Phosphatidylcholines		Level 2
PC aa C36:4	C(18:2;9Z,12Z-dienoyl/18:2;9Z,12Z-dienoyl) aa D-Phosphatidylcholine	Phosphatidylcholines		Level 2
PC aa C36:4	PC(18:2(9Z,12Z)/18:2(9Z,12Z))	Phosphatidylcholines	HMDB08138	Level 2
PC aa C36:4	C(18:2;9Z,12Z-dienoyl/18:2;9Z,12Z-dienoyl) aa Phosphatidylcholine	Phosphatidylcholines		Level 2
PC aa C36:4	PC(18:1(9Z)/18:3(6Z,9Z,12Z))	Phosphatidylcholines	HMDB08106	Level 2
PC aa C36:4	PC(18:1(9Z)/18:3(9Z,12Z,15Z))	Phosphatidylcholines	HMDB08107	Level 2
PC aa C36:4	C(18:3;9Z,12Z,15Z-trienoyl/18:1;9Z-enoyl) aa Phosphatidylcholine	Phosphatidylcholines		Level 2
PC aa C36:4	C(18:4;5Z,8Z,11Z,14Z-tetraenoyl/18:0) aa Phosphatidylcholine	Phosphatidylcholines		Level 2
PC aa C36:4	PC(18:0/18:4(6Z,9Z,12Z,15Z))	Phosphatidylcholines	HMDB08042	Level 2
PC aa C36:4	C(18:4;9E,11E,13E,15E-tetraenoyl/18:0) aa L-Phosphatidylcholine	Phosphatidylcholines		Level 2
PC aa C36:4	C(18:4;9E,11E,13E,15E-tetraenoyl/18:0) aa Phosphatidylcholine	Phosphatidylcholines		Level 2
PC aa C36:4	C(18:4;9E,11E,13Z,15Z-tetraenoyl/18:0) aa Phosphatidylcholine	Phosphatidylcholines		Level 2
PC aa C36:4	PC(16:0/20:4(5Z,8Z,11Z,14Z))	Phosphatidylcholines	HMDB07982	Level 2
PC aa C36:4	C(20:4;5E,8E,11E,14E-tetraenoyl/16:0) aa Phosphatidylcholine	Phosphatidylcholines		Level 2
PC aa C36:4	C(20:4;5Z,8Z,11Z,14Z-tetraenoyl/16:0) aa L-Phosphatidylcholine	Phosphatidylcholines		Level 2
PC aa C36:4	C(20:4;5Z,8Z,11Z,14Z-tetraenoyl/16:0) aa Phosphatidylcholine	Phosphatidylcholines		Level 2
PC aa C36:5	C(18:1;11Z-enoyl/18:4;2E,4E,6E,11Z-tetraenoyl) aa Phosphatidylcholine	Phosphatidylcholines		Level 2
PC aa C36:5	C(20:4;5Z,8Z,11Z,14Z-tetraenoyl/16:1;7Z-enoyl) aa L-Phosphatidylcholine	Phosphatidylcholines		Level 2
PC aa C36:5	PC(16:1(9Z)/20:4(5Z,8Z,11Z,14Z))	Phosphatidylcholines	HMDB08015	Level 2
PC aa C36:5	C(20:4;5Z,8Z,11Z,14Z-tetraenoyl/16:1;9Z-enoyl) aa Phosphatidylcholine	Phosphatidylcholines		Level 2
PC aa C36:5	C(20:5;5E,8E,11E,14E,17E-pentaenoyl/16:0) aa Phosphatidylcholine	Phosphatidylcholines		Level 2
PC aa C36:5	PC(16:0/20:5(5Z,8Z,11Z,14Z,17Z))	Phosphatidylcholines	HMDB07984	Level 2
PC aa C36:5	C(20:5;5Z,8Z,11Z,14Z,17Z-pentaenoyl/16:0) aa Phosphatidylcholine	Phosphatidylcholines		Level 2
PC aa C36:6	C(18:2;9Z,12Z-dienoyl/18:4;9E,11E,13E,15E-tetraenoyl) aa L-Phosphatidylcholine	Phosphatidylcholines		Level 2
PC aa C36:6	C(18:3;6Z,9Z,12Z-trienoyl/18:3;6Z,9Z,12Z-trienoyl) aa Phosphatidylcholine	Phosphatidylcholines		Level 2
PC aa C36:6	C(18:3;8E,10E,12E-trienoyl/18:3;8E,10E,12E-trienoyl) aa Phosphatidylcholine	Phosphatidylcholines		Level 2
PC aa C36:6	C(18:3;9E,11E,13E-trienoyl/18:3;9E,11E,13E-trienoyl) aa L-Phosphatidylcholine	Phosphatidylcholines		Level 2
PC aa C36:6	C(18:3;9E,11E,13E-trienoyl/18:3;9E,11E,13E-trienoyl) aa Phosphatidylcholine	Phosphatidylcholines		Level 2
PC aa C36:6	C(18:3;9Z,11E,13E-trienoyl/18:3;9Z,11E,13E-trienoyl) aa L-Phosphatidylcholine	Phosphatidylcholines		Level 2

Metabolomics

Abbreviation	Name	Metabolite class	HMDB ID	MSI level
PC aa C36:6	PC(18:3(9Z,12Z,15Z)/18:3(9Z,12Z,15Z))	Phosphatidylcholines	HMDB08206	Level 2
PC aa C36:6	C(18:3;9Z,12Z,15Z-trienoyl/18:3;9Z,12Z,15Z-trienoyl) aa Phosphatidylcholine	Phosphatidylcholines		Level 2
PC aa C36:6	PC(14:0/22:6(4Z,7Z,10Z,13Z,16Z,19Z))	Phosphatidylcholines	HMDB07892	Level 2
PC aa C36:6	C(22:6;4Z,7Z,10Z,13Z,16Z,19Z-hexaenoyl/14:0) aa Phosphatidylcholine	Phosphatidylcholines		Level 2
PC aa C38:0	C(12:0/26:0) aa Phosphatidylcholine	Phosphatidylcholines		Level 2
PC aa C38:0	C(13:0/25:0) aa Phosphatidylcholine	Phosphatidylcholines		Level 2
PC aa C38:0	PC(24:0/14:0)	Phosphatidylcholines	HMDB08755	Level 2
PC aa C38:0	C(15:0/23:0) aa Phosphatidylcholine	Phosphatidylcholines		Level 2
PC aa C38:0	C(16:0/22:0) aa L-Phosphatidylcholine	Phosphatidylcholines		Level 2
PC aa C38:0	PC(22:0/16:0)	Phosphatidylcholines	HMDB08528	Level 2
PC aa C38:0	PC(20:0/18:0)	Phosphatidylcholines	HMDB08267	Level 2
PC aa C38:0	C(19:0/19:0) aa L-Phosphatidylcholine	Phosphatidylcholines		Level 2
PC aa C38:0	C(19:0/19:0) aa Phosphatidylcholine	Phosphatidylcholines		Level 2
PC aa C38:0	PC(18:0/20:0)	Phosphatidylcholines	HMDB08043	Level 2
PC aa C38:0	C(20:0/18:0) aa Phosphatidylcholine	Phosphatidylcholines		Level 2
PC aa C38:0	C(21:0/17:0) aa Phosphatidylcholine	Phosphatidylcholines		Level 2
PC aa C38:0	C(17:0/21:0) aa Phosphatidylcholine	Phosphatidylcholines		Level 2
PC aa C38:0	PC(16:0/22:0)	Phosphatidylcholines	HMDB07985	Level 2
PC aa C38:0	C(22:0/16:0) aa Phosphatidylcholine	Phosphatidylcholines		Level 2
PC aa C38:0	C(23:0/15:0) aa Phosphatidylcholine	Phosphatidylcholines		Level 2
PC aa C38:0	PC(14:0/24:0)	Phosphatidylcholines	HMDB07893	Level 2
PC aa C38:0	C(25:0/13:0) aa L-Phosphatidylcholine	Phosphatidylcholines		Level 2
PC aa C38:1	C(18:1;11E-enoyl/20:0) aa Phosphatidylcholine	Phosphatidylcholines		Level 2
PC aa C38:1	PC(20:0/18:1(11Z))	Phosphatidylcholines	HMDB08268	Level 2
PC aa C38:1	C(18:1;13E-enoyl/20:0) aa Phosphatidylcholine	Phosphatidylcholines		Level 2
PC aa C38:1	C(18:1;6Z-enoyl/20:0) aa L-Phosphatidylcholine	Phosphatidylcholines		Level 2
PC aa C38:1	C(18:1;7Z-enoyl/20:0) aa L-Phosphatidylcholine	Phosphatidylcholines		Level 2
PC aa C38:1	PC(20:0/18:1(9Z))	Phosphatidylcholines	HMDB08269	Level 2
PC aa C38:1	C(18:1;9Z-enoyl/20:0) aa Phosphatidylcholine	Phosphatidylcholines		Level 2
PC aa C38:1	C(20:0/18:1;9E-enoyl) aa L-Phosphatidylcholine	Phosphatidylcholines		Level 2
PC aa C38:1	C(20:0/18:1;9E-enoyl) aa Phosphatidylcholine	Phosphatidylcholines		Level 2
PC aa C38:1	PC(18:1(9Z)/20:0)	Phosphatidylcholines	HMDB08109	Level 2
PC aa C38:1	C(20:0/18:1;9Z-enoyl) aa Phosphatidylcholine	Phosphatidylcholines		Level 2
PC aa C38:1	PC(18:0/20:1(11Z))	Phosphatidylcholines	HMDB08044	Level 2
PC aa C38:1	C(20:1;13Z-enoyl/18:0) aa L-Phosphatidylcholine	Phosphatidylcholines		Level 2
PC aa C38:1	C(20:1;14Z-enoyl/18:0) aa L-Phosphatidylcholine	Phosphatidylcholines		Level 2
PC aa C38:1	C(20:1;5Z-enoyl/18:0) aa L-Phosphatidylcholine	Phosphatidylcholines		Level 2
PC aa C38:1	C(20:1;8Z-enoyl/18:0) aa L-Phosphatidylcholine	Phosphatidylcholines		Level 2

Metabolomics

Abbreviation	Name	Metabolite class	HMDB ID	MSI level
PC aa C38:1	PC(16:0/22:1(13Z))	Phosphatidylcholines	HMDB07986	Level 2
PC aa C38:1	C(22:1;13Z-enoyl/16:0) aa Phosphatidylcholine	Phosphatidylcholines		Level 2
PC aa C38:1	PC(14:0/24:1(15Z))	Phosphatidylcholines	HMDB07894	Level 2
PC aa C38:3	C(20:1;13Z-enoyl/18:2;9Z,12Z-dienoyl) aa L-Phosphatidylcholine	Phosphatidylcholines		Level 2
PC aa C38:3	C(20:3;5E,8E,11E-trienoyl/18:0) aa Phosphatidylcholine	Phosphatidylcholines		Level 2
PC aa C38:3	C(20:3;5Z,11Z,14Z-trienoyl/18:0) aa L-Phosphatidylcholine	Phosphatidylcholines		Level 2
PC aa C38:3	PC(18:0/20:3(5Z,8Z,11Z))	Phosphatidylcholines	HMDB08046	Level 2
PC aa C38:3	C(20:3;5Z,8Z,11Z-trienoyl/18:0) aa Phosphatidylcholine	Phosphatidylcholines		Level 2
PC aa C38:3	C(20:3;5Z,8Z,14Z-trienoyl/18:0) aa L-Phosphatidylcholine	Phosphatidylcholines		Level 2
PC aa C38:3	PC(18:0/20:3(8Z,11Z,14Z))	Phosphatidylcholines	HMDB08047	Level 2
PC aa C38:3	C(20:3;8Z,11Z,14Z-trienoyl/18:0) aa Phosphatidylcholine	Phosphatidylcholines		Level 2
PC aa C38:3	C(22:3;13Z,16Z,19Z-trienoyl/16:0) aa Phosphatidylcholine	Phosphatidylcholines		Level 2
PC aa C38:4	PC(18:1(9Z)/20:3(5Z,8Z,11Z))	Phosphatidylcholines	HMDB08112	Level 2
PC aa C38:4	C(20:4;5E,8E,11E,14E-tetraenoyl/18:0) aa Phosphatidylcholine	Phosphatidylcholines		Level 2
PC aa C38:4	PC(18:0/20:4(5Z,8Z,11Z,14Z))	Phosphatidylcholines	HMDB08048	Level 2
PC aa C38:4	C(20:4;5Z,8Z,11Z,14Z-tetraenoyl/18:0) aa Phosphatidylcholine	Phosphatidylcholines		Level 2
PC aa C38:4	C(20:4;8Z,10Z,12Z,14Z-tetraenoyl/18:0) aa L-Phosphatidylcholine	Phosphatidylcholines		Level 2
PC aa C38:4	PC(16:0/22:4(7Z,10Z,13Z,16Z))	Phosphatidylcholines	HMDB07988	Level 2
PC aa C38:4	C(22:4;7Z,10Z,13Z,16Z-tetraenoyl/16:0) aa Phosphatidylcholine	Phosphatidylcholines		Level 2
PC aa C38:5	PC(18:1(9Z)/20:4(5Z,8Z,11Z,14Z))	Phosphatidylcholines	HMDB08114	Level 2
PC aa C38:5	C(20:4;5Z,8Z,11Z,14Z-tetraenoyl/18:1;9Z-enoyl) aa Phosphatidylcholine	Phosphatidylcholines		Level 2
PC aa C38:5	PC(18:0/20:5(5Z,8Z,11Z,14Z,17Z))	Phosphatidylcholines	HMDB08050	Level 2
PC aa C38:5	C(20:5;5Z,8Z,11Z,14Z,17Z-pentaenoyl/18:0) aa Phosphatidylcholine	Phosphatidylcholines		Level 2
PC aa C38:5	C(20:5;9Z,11Z,13Z,15Z,17Z-pentaenoyl/18:0) aa L-Phosphatidylcholine	Phosphatidylcholines		Level 2
PC aa C38:5	C(22:5;4E,7E,10E,13E,16E-pentaenoyl/16:0) aa Phosphatidylcholine	Phosphatidylcholines		Level 2
PC aa C38:5	PC(16:0/22:5(4Z,7Z,10Z,13Z,16Z))	Phosphatidylcholines	HMDB07989	Level 2
PC aa C38:5	C(22:5;4Z,7Z,10Z,13Z,16Z-pentaenoyl/16:0) aa Phosphatidylcholine	Phosphatidylcholines		Level 2
PC aa C38:5	PC(16:0/22:5(7Z,10Z,13Z,16Z,19Z))	Phosphatidylcholines	HMDB07990	Level 2
PC aa C38:5	C(22:5;7Z,10Z,13Z,16Z,19Z-pentaenoyl/16:0) aa Phosphatidylcholine	Phosphatidylcholines		Level 2
PC aa C38:6	C(16:0/22:6;4E,7E,10E,13E,16E,19E-hexaenoyl) aa L-Phosphatidylcholine	Phosphatidylcholines		Level 2
PC aa C38:6	PC(22:6(4Z,7Z,10Z,13Z,16Z,19Z)/16:0)	Phosphatidylcholines	HMDB08725	Level 2
PC aa C38:6	C(16:0/22:6;4Z,7Z,10Z,13Z,16Z,19Z-hexaenoyl) aa Phosphatidylcholine	Phosphatidylcholines		Level 2
PC aa C38:6	PC(20:5(5Z,8Z,11Z,14Z,17Z)/18:1(9Z))	Phosphatidylcholines	HMDB08499	Level 2
PC aa C38:6	PC(20:4(5Z,8Z,11Z,14Z)/18:2(9Z,12Z))	Phosphatidylcholines	HMDB08434	Level 2

Abbreviation	Name	Metabolite class	HMDB ID	MSI level
PC aa C38:6	PC(18:2(9Z,12Z)/20:4(5Z,8Z,11Z,14Z))	Phosphatidylcholines	HMDB08147	Level 2
PC aa C38:6	C(20:4;5Z,8Z,11Z,14Z-tetraenoyl/18:2;9Z,12Z-dienoyl) aa Phosphatidylcholine	Phosphatidylcholines		Level 2
PC aa C38:6	PC(18:1(11Z)/20:5(5Z,8Z,11Z,14Z,17Z))	Phosphatidylcholines	HMDB08083	Level 2
PC aa C38:6	PC(18:1(9Z)/20:5(5Z,8Z,11Z,14Z,17Z))	Phosphatidylcholines	HMDB08116	Level 2
PC aa C38:6	C(20:5;5Z,8Z,11Z,14Z,17Z-pentaenoyl/18:1;9Z-enoyl) aa Phosphatidylcholine	Phosphatidylcholines		Level 2
PC aa C38:6	C(22:6;3Z,6Z,9Z,12Z,15Z,18E-hexaenoyl/16:0) aa Phosphatidylcholine	Phosphatidylcholines		Level 2
PC aa C38:6	C(22:6;4E,7E,10E,13E,16E,19E-hexaenoyl/16:0) aa L-Phosphatidylcholine	Phosphatidylcholines		Level 2
PC aa C38:6	C(22:6;4E,7E,10E,13E,16E,19E-hexaenoyl/16:0) aa Phosphatidylcholine	Phosphatidylcholines		Level 2
PC aa C38:6	C(22:6;4Z,7Z,10Z,13Z,16Z,19Z-hexaenoyl/16:0) aa D-Phosphatidylcholine	Phosphatidylcholines		Level 2
PC aa C38:6	PC(16:0/22:6(4Z,7Z,10Z,13Z,16Z,19Z))	Phosphatidylcholines	HMDB07991	Level 2
PC aa C38:6	C(22:6;4Z,7Z,10Z,13Z,16Z,19Z-hexaenoyl/16:0) aa Phosphatidylcholine	Phosphatidylcholines		Level 2
PC aa C40:1	PC(20:0/20:1(11Z))	Phosphatidylcholines	HMDB08275	Level 2
PC aa C40:1	C(20:1;13Z-enoyl/20:0) aa L-Phosphatidylcholine	Phosphatidylcholines		Level 2
PC aa C40:1	C(20:1;5Z-enoyl/20:0) aa L-Phosphatidylcholine	Phosphatidylcholines		Level 2
PC aa C40:1	C(20:1;8Z-enoyl/20:0) aa L-Phosphatidylcholine	Phosphatidylcholines		Level 2
PC aa C40:1	C(22:0/18:1;11E-enoyl) aa L-Phosphatidylcholine	Phosphatidylcholines		Level 2
PC aa C40:1	PC(18:1(11Z)/22:0)	Phosphatidylcholines	HMDB08084	Level 2
PC aa C40:1	C(22:0/18:1;9E-enoyl) aa Phosphatidylcholine	Phosphatidylcholines		Level 2
PC aa C40:1	PC(18:1(9Z)/22:0)	Phosphatidylcholines	HMDB08117	Level 2
PC aa C40:1	C(22:0/18:1;9Z-enoyl) aa Phosphatidylcholine	Phosphatidylcholines		Level 2
PC aa C40:1	PC(18:0/22:1(13Z))	Phosphatidylcholines	HMDB08052	Level 2
PC aa C40:1	C(24:1;E15-enoyl/16:0) aa Phosphatidylcholine	Phosphatidylcholines		Level 2
PC aa C40:1	C(24:1;Z15-enoyl/16:0) aa D-Phosphatidylcholine	Phosphatidylcholines		Level 2
PC aa C40:1	PC(16:0/24:1(15Z))	Phosphatidylcholines	HMDB07993	Level 2
PC aa C40:1	C(24:1;Z15-enoyl/16:0) aa Phosphatidylcholine	Phosphatidylcholines		Level 2
PC aa C40:2	C(20:1;11E-enoyl/20:1;11E-enoyl) aa L-Phosphatidylcholine	Phosphatidylcholines		Level 2
PC aa C40:2	PC(20:1(11Z)/20:1(11Z))	Phosphatidylcholines	HMDB08308	Level 2
PC aa C40:2	C(20:1;11Z-enoyl/20:1;11Z-enoyl) aa Phosphatidylcholine	Phosphatidylcholines		Level 2
PC aa C40:2	C(20:1;9E-enoyl/20:1;9E-enoyl) aa L-Phosphatidylcholine	Phosphatidylcholines		Level 2
PC aa C40:2	C(20:1;9Z-enoyl/20:1;9Z-enoyl) aa L-Phosphatidylcholine	Phosphatidylcholines		Level 2
PC aa C40:2	C(20:1;9Z-enoyl/20:1;9Z-enoyl) aa Phosphatidylcholine	Phosphatidylcholines		Level 2
PC aa C40:2	PC(20:0/20:2(11Z,14Z))	Phosphatidylcholines	HMDB08276	Level 2
PC aa C40:3	C(20:3;11Z,14Z.17Z-trienoyl/20:0) aa L-Phosphatidylcholine	Phosphatidylcholines		Level 2
PC aa C40:3	C(22:3;10Z,13Z,16Z-trienoyl/18:0) aa L-Phosphatidylcholine	Phosphatidylcholines		Level 2

Metabolomics

Abbreviation	Name	Metabolite class	HMDB ID	MSI level
PC aa C40:4	PC(22:4(7Z,10Z,13Z,16Z)/18:0)	Phosphatidylcholines	HMDB08628	Level 2
PC aa C40:4	C(20:2;11E,14E-dienoyl/20:2;11E,14E-dienoyl) aa L-Phosphatidylcholine	Phosphatidylcholines		Level 2
PC aa C40:4	C(20:2;2E,4E-dienoyl/20:2;2E,4E-dienoyl) aa D-Phosphatidylcholine	Phosphatidylcholines		Level 2
PC aa C40:4	PC(20:0/20:4(5Z,8Z,11Z,14Z))	Phosphatidylcholines	HMDB08279	Level 2
PC aa C40:4	PC(18:0/22:4(7Z,10Z,13Z,16Z))	Phosphatidylcholines	HMDB08054	Level 2
PC aa C40:4	C(22:3;7Z10Z,13Z,16Z-tetraenoyl/18:0) aa Phosphatidylcholine	Phosphatidylcholines		Level 2
PC aa C40:5	PC(18:1(9Z)/22:4(7Z,10Z,13Z,16Z))	Phosphatidylcholines	HMDB08120	Level 2
PC aa C40:5	C(22:5;4E,7E,10E,13E,16E-pentaenoyl/18:0) aa Phosphatidylcholine	Phosphatidylcholines		Level 2
PC aa C40:5	PC(18:0/22:5(4Z,7Z,10Z,13Z,16Z))	Phosphatidylcholines	HMDB08055	Level 2
PC aa C40:5	C(22:5;4Z,7Z,10Z,13Z,16Z-pentaenoyl/18:0) aa Phosphatidylcholine	Phosphatidylcholines		Level 2
PC aa C40:5	PC(18:0/22:5(7Z,10Z,13Z,16Z,19Z))	Phosphatidylcholines	HMDB08056	Level 2
PC aa C40:5	C(22:5;7Z,10Z,13Z,16Z,19Z-pentaenoyl/18:0) aa Phosphatidylcholine	Phosphatidylcholines		Level 2
PC aa C40:6	PC(18:1(11Z)/22:5(7Z,10Z,13Z,16Z,19Z))	Phosphatidylcholines	HMDB08089	Level 2
PC aa C40:6	PC(18:1(9Z)/22:5(7Z,10Z,13Z,16Z,19Z))	Phosphatidylcholines	HMDB08122	Level 2
PC aa C40:6	C(22:6;4E,7E,10E,13E,16E,19E-hexaenoyl/18:0) aa Phosphatidylcholine	Phosphatidylcholines		Level 2
PC aa C40:6	PC(18:0/22:6(4Z,7Z,10Z,13Z,16Z,19Z))	Phosphatidylcholines	HMDB08057	Level 2
PC aa C40:6	C(22:6;4Z,7Z,10Z,13Z,16Z,19Z-hexaenoyl/18:0) aa Phosphatidylcholine	Phosphatidylcholines		Level 2
PC aa C40:6	C(22:6;9Z,11Z,13Z,15Z,17Z,19E-hexaenoyl/18:0) aa L-Phosphatidylcholine	Phosphatidylcholines		Level 2
PC aa C42:0	C(16:0/26:0) aa L-Phosphatidylcholine	Phosphatidylcholines		Level 2
PC aa C42:0	C(17:0/25:0) aa L-Phosphatidylcholine	Phosphatidylcholines		Level 2
PC aa C42:0	PC(24:0/18:0)	Phosphatidylcholines	HMDB08760	Level 2
PC aa C42:0	C(19:0/23:0) aa Phosphatidylcholine	Phosphatidylcholines		Level 2
PC aa C42:0	PC(22:0/20:0)	Phosphatidylcholines	HMDB08537	Level 2
PC aa C42:0	C(21:0/21:0) aa L-Phosphatidylcholine	Phosphatidylcholines		Level 2
PC aa C42:0	C(21:0/21:0) aa Phosphatidylcholine	Phosphatidylcholines		Level 2
PC aa C42:0	PC(20:0/22:0)	Phosphatidylcholines	HMDB08282	Level 2
PC aa C42:0	C(23:0/19:0) aa Phosphatidylcholine	Phosphatidylcholines		Level 2
PC aa C42:0	PC(18:0/24:0)	Phosphatidylcholines	HMDB08058	Level 2
PC aa C42:0	C(25:0/17:0) aa Phosphatidylcholine	Phosphatidylcholines		Level 2
PC aa C42:0	C(26:0/16:0) aa L-Phosphatidylcholine	Phosphatidylcholines		Level 2
PC aa C42:0	C(26:0/16:0) aa Phosphatidylcholine	Phosphatidylcholines		Level 2
PC aa C42:1	C(18:1;6Z-enoyl/24:0) aa L-Phosphatidylcholine	Phosphatidylcholines		Level 2
PC aa C42:1	PC(24:0/18:1(9Z))	Phosphatidylcholines	HMDB08762	Level 2
PC aa C42:1	PC(22:0/20:1(11Z))	Phosphatidylcholines	HMDB08538	Level 2
PC aa C42:1	C(22:0/20:1;11E-enoyl) aa L-Phosphatidylcholine	Phosphatidylcholines		Level 2
PC aa C42:1	C(22:0/20:1;11Z-enoyl) aa L-Phosphatidylcholine	Phosphatidylcholines		Level 2
PC aa C42:1	PC(20:0/22:1(13Z))	Phosphatidylcholines	HMDB08283	Level 2

Metabolomics

Abbreviation	Name	Metabolite class	HMDB ID	MSI level
PC aa C42:1	PC(18:1(9Z)/24:0)	Phosphatidylcholines	HMDB08124	Level 2
PC aa C42:1	PC(18:0/24:1(15Z))	Phosphatidylcholines	HMDB08059	Level 2
PC aa C42:2	C(21:1;12Z-enoyl/21:1;12Z-enoyl) aa Phosphatidylcholine	Phosphatidylcholines		Level 2
PC aa C42:2	C(26:2;5Z,9Z-dienoyl/16:0) aa L-Phosphatidylcholine	Phosphatidylcholines		Level 2
PC aa C42:2	C16:0/26:1;5Z,9Z-dienoyl) aa L-Phosphatidylcholine	Phosphatidylcholines		Level 2
PC aa C42:4	Phosphatidylcholine with diacyl residue sum C42:4	Phosphatidylcholines		Level 2
PC aa C42:5	PC(20:0/22:5(7Z,10Z,13Z,16Z,19Z))	Phosphatidylcholines	HMDB08287	Level 2
PC aa C42:6	C(22:6;4Z,7Z,10Z,13Z,16Z,19Z-hexaenoyl/20:0) aa Phosphatidylcholine	Phosphatidylcholines		Level 2
PC aa C42:6	PC(20:0/22:6(4Z,7Z,10Z,13Z,16Z,19Z))	Phosphatidylcholines	HMDB08288	Level 2
PC ae C30:0	C(16:0/e14:0) ae Phosphatidylcholine	Phosphatidylcholines		Level 2
PC ae C30:0	PC(o-14:0/16:0)	Phosphatidylcholines	HMDB13341	Level 2
PC ae C30:1	PC(o-14:0/16:1(9Z))	Phosphatidylcholines	HMDB13402	Level 2
PC ae C30:2	Phosphatidylcholine with acyl-alkyl residue sum C30:2	Phosphatidylcholines		Level 2
PC ae C32:1	C(18:1;9Z-enoyl/e14:0) ae L-Phosphatidylcholine	Phosphatidylcholines		Level 2
PC ae C32:2	C(18:1;9Z-enoyl/e14:1;1Z-en) ae L-Phosphatidylcholine	Phosphatidylcholines		Level 2
PC ae C32:2	C(18:2;9Z,12Z-dienoyl/e14:0) ae L-Phosphatidylcholine	Phosphatidylcholines		Level 2
PC ae C34:0	C(16:0/e18:0) ae D-Phosphatidylcholine	Phosphatidylcholines		Level 2
PC ae C34:0	C(16:0/e18:0) ae L-Phosphatidylcholine	Phosphatidylcholines		Level 2
PC ae C34:0	C(16:0/e18:0) ae Phosphatidylcholine	Phosphatidylcholines		Level 2
PC ae C34:0	C(17:0/e17:0) ae L-Phosphatidylcholine	Phosphatidylcholines		Level 2
PC ae C34:0	C(18:0/e16:0) ae D-Phosphatidylcholine	Phosphatidylcholines		Level 2
PC ae C34:0	PC(o-16:0/18:0)	Phosphatidylcholines	HMDB13405	Level 2
PC ae C34:0	C(18:0/e16:0) ae Phosphatidylcholine	Phosphatidylcholines		Level 2
PC ae C34:0	C(e16:0/18:0) ae Phosphatidylcholine	Phosphatidylcholines		Level 2
PC ae C34:1	C(16:0;e18:1;9Z-en) ae Phosphatidylcholine	Phosphatidylcholines		Level 2
PC ae C34:1	C(16:1;9Z-enoyl/e18:0) ae L-Phosphatidylcholine	Phosphatidylcholines		Level 2
PC ae C34:1	C(18:1;9E-enoyl/e16:0) ae Phosphatidylcholine	Phosphatidylcholines		Level 2
PC ae C34:1	C(18:1;9Z-enoyl/e16:0) ae D-Phosphatidylcholine	Phosphatidylcholines		Level 2
PC ae C34:1	C(18:1;9Z-enoyl/e16:0) ae L-Phosphatidylcholine	Phosphatidylcholines		Level 2
PC ae C34:1	C(18:1;9Z-enoyl/e16:0) ae Phosphatidylcholine	Phosphatidylcholines		Level 2
PC ae C34:2	PC(O-16:0/18:2(9Z,12Z))	Phosphatidylcholines	HMDB11151	Level 2
PC ae C34:2	C(18:1;9Z,12Z-dienoyl/e16:0) ae Phosphatidylcholine	Phosphatidylcholines		Level 2
PC ae C34:2	C(18:1;9Z-enoyl/e16:1;1E-en) ae Phosphatidylcholine	Phosphatidylcholines		Level 2
PC ae C34:2	C(18:1;9Z-enoyl/e16:1;1Z-en) ae L-Phosphatidylcholine	Phosphatidylcholines		Level 2
PC ae C34:2	C(18:1;9Z-enoyl/e16:1;1Z-en) ae Phosphatidylcholine	Phosphatidylcholines		Level 2
PC ae C34:3	C(18:1;6Z,9Z,12Z-trienoyl/e16:0) ae L-	Phosphatidylcholines		Level 2

Abbreviation	Name	Metabolite class	HMDB ID	MSI level
	Phosphatidylcholine			
PC ae C34:3	C(18:1;9Z,12Z,15Z-trienoyl/e16:0) ae D-Phosphatidylcholine	Phosphatidylcholines		Level 2
PC ae C34:3	C(18:1;9Z,12Z,15Z-trienoyl/e16:0) ae L-Phosphatidylcholine	Phosphatidylcholines		Level 2
PC ae C34:3	PC(P-16:0/18:2(9Z,12Z))	Phosphatidylcholines	HMDB11211	Level 2
PC ae C36:0	C(16:0/e20:0) ae L-Phosphatidylcholine	Phosphatidylcholines		Level 2
PC ae C36:0	C(18:0/e18:0) ae D-Phosphatidylcholine	Phosphatidylcholines		Level 2
PC ae C36:0	PC(o-18:0/18:0)	Phosphatidylcholines	HMDB13417	Level 2
PC ae C36:0	C(18:0/e18:0) ae Phosphatidylcholine	Phosphatidylcholines		Level 2
PC ae C36:0	PC(o-16:0/20:0)	Phosphatidylcholines	HMDB13406	Level 2
PC ae C36:0	C(22:0/e14:0) ae L-Phosphatidylcholine	Phosphatidylcholines		Level 2
PC ae C36:1	C(20:1;9Z-enoyl/e16:0) ae L-Phosphatidylcholine	Phosphatidylcholines		Level 2
PC ae C36:2	PC(P-18:0/18:1(9Z))	Phosphatidylcholines	HMDB11243	Level 2
PC ae C36:3	C(20:3;8Z,11Z,14Z-trienoyl/e16:0) ae L-Phosphatidylcholine	Phosphatidylcholines		Level 2
PC ae C36:4	C(20:4;5E,8E,11E,14E-tetraenoyl/e16:0) ae L-Phosphatidylcholine	Phosphatidylcholines		Level 2
PC ae C36:4	C(20:4;5E,8E,11E,14E-tetraenoyl/e16:0) ae Phosphatidylcholine	Phosphatidylcholines		Level 2
PC ae C36:4	C(20:4;5Z,8Z,11Z,14Z-tetraenoyl/e16:0) ae L-Phosphatidylcholine	Phosphatidylcholines		Level 2
PC ae C36:4	C(20:4;5Z,8Z,11Z,14Z-tetraenoyl/e16:0) ae Phosphatidylcholine	Phosphatidylcholines		Level 2
PC ae C36:5	PC(P-16:0/20:4(5Z,8Z,11Z,14Z))	Phosphatidylcholines	HMDB11220	Level 2
PC ae C36:5	C(20:5;(5Z,8Z,11Z,14Z,17Z-pentaenoyl/e16:0) ae L-Phosphatidylcholine	Phosphatidylcholines		Level 2
PC ae C38:0	PC(o-18:0/20:0)	Phosphatidylcholines	HMDB13419	Level 2
PC ae C38:0	C(20:0/e18:0) ae Phosphatidylcholine	Phosphatidylcholines		Level 2
PC ae C38:0	PC(o-16:0/22:0)	Phosphatidylcholines	HMDB13408	Level 2
PC ae C38:0	C(22:0/e16:0) ae Phosphatidylcholine	Phosphatidylcholines		Level 2
PC ae C38:1	C(20:1;9Z-enoyl/e18:0) ae L-Phosphatidylcholine	Phosphatidylcholines		Level 2
PC ae C38:2	Phosphatidylcholine with acyl-alkyl residue sum C38:2	Phosphatidylcholines		Level 2
PC ae C38:3	Phosphatidylcholine with acyl-alkyl residue sum C38:3	Phosphatidylcholines		Level 2
PC ae C38:4	C(20:4;5E,8E,11E,14E-tetraenoyl/e18:0) ae L-Phosphatidylcholine	Phosphatidylcholines		Level 2
PC ae C38:4	C(20:4;5E,8E,11E,14E-tetraenoyl/e18:0) ae Phosphatidylcholine	Phosphatidylcholines		Level 2
PC ae C38:4	PC(o-18:0/20:4(8Z,11Z,14Z,17Z))	Phosphatidylcholines	HMDB13420	Level 2
PC ae C38:4	C(20:4;5Z,8Z,11Z,14Z-tetraenoyl/e18:0) ae Phosphatidylcholine	Phosphatidylcholines		Level 2
PC ae C38:4	C(22:4;7Z,10Z,13Z,16Z-tetraenoyl/e16:0) ae Phosphatidylcholine	Phosphatidylcholines		Level 2
PC ae C38:5	PC(o-18:1(9Z)/20:4(8Z,11Z,14Z,17Z))	Phosphatidylcholines	HMDB13432	Level 2
PC ae C38:5	C(20:4;5Z,8Z,11Z,14Z-tetraenoyl/e18:1;1Z-en) ae L-Phosphatidylcholine	Phosphatidylcholines		Level 2
PC ae C38:5	C(20:5;5Z,8Z,11Z,14Z,17Z-pentaenoyl/e18:0) ae L-Phosphatidylcholine	Phosphatidylcholines		Level 2
PC ae C38:5	C(22:5;7Z,10Z,13Z,16Z,19Z-pentaenoyl/e16:0)	Phosphatidylcholines		Level 2

Metabolomics

Abbreviation	Name	Metabolite class	HMDB ID	MSI level
	ae L-Phosphatidylcholine			
PC ae C38:5	C(22:5;7Z,10Z,13Z,16Z,19Z-pentaenoyl/e16:0) ae Phosphatidylcholine	Phosphatidylcholines		Level 2
PC ae C38:6	PC(o-16:0/22:6(4Z,7Z,10Z,13Z,16Z,19Z))	Phosphatidylcholines	HMDB13409	Level 2
PC ae C38:6	C(22:6;4Z,7Z,10Z,13Z,16Z,19Z-pentaenoyl/e16:0) ae Phosphatidylcholine	Phosphatidylcholines		Level 2
PC ae C40:0	PC(o-18:0/22:0)	Phosphatidylcholines	HMDB13421	Level 2
PC ae C40:0	C(22:0/e18:0) ae Phosphatidylcholine	Phosphatidylcholines		Level 2
PC ae C40:1	Phosphatidylcholine with acyl-alkyl residue sum C40:1	Phosphatidylcholines		Level 2
PC ae C40:2	Phosphatidylcholine with acyl-alkyl residue sum C40:2	Phosphatidylcholines		Level 2
PC ae C40:3	Phosphatidylcholine with acyl-alkyl residue sum C40:3	Phosphatidylcholines		Level 2
PC ae C40:4	Phosphatidylcholine with acyl-alkyl residue sum C40:4	Phosphatidylcholines		Level 2
PC ae C40:5	C(22:5;4Z,7Z,10Z,13Z,16Z-pentaenoyl/e18:0) ae L-Phosphatidylcholine	Phosphatidylcholines		Level 2
PC ae C40:5	C(22:5;7E,10E,13E,16E,19E-pentaenoyl/e18:0) ae Phosphatidylcholine	Phosphatidylcholines		Level 2
PC ae C40:5	C(22:5;7Z,10Z,13Z,16Z,19Z-pentaenoyl/e18:0) ae L-Phosphatidylcholine	Phosphatidylcholines		Level 2
PC ae C40:6	PC(o-18:0/22:6(4Z,7Z,10Z,13Z,16Z,19Z))	Phosphatidylcholines	HMDB13422	Level 2
PC ae C40:6	C(22:5;4Z,7Z,10Z,13Z,16Z,19Z-hexaenoyl/e18:0) ae Phosphatidylcholine	Phosphatidylcholines		Level 2
PC ae C42:0	PC(o-20:0/22:0)	Phosphatidylcholines	HMDB13443	Level 2
PC ae C42:1	Phosphatidylcholine with acyl-alkyl residue sum C42:1	Phosphatidylcholines		Level 2
PC ae C42:2	Phosphatidylcholine with acyl-alkyl residue sum C42:2	Phosphatidylcholines		Level 2
PC ae C42:3	Phosphatidylcholine with acyl-alkyl residue sum C42:3	Phosphatidylcholines		Level 2
PC ae C42:4	Phosphatidylcholine with acyl-alkyl residue sum C42:4	Phosphatidylcholines		Level 2
PC ae C42:5	Phosphatidylcholine with acyl-alkyl residue sum C42:5	Phosphatidylcholines		Level 2
PC ae C44:3	Phosphatidylcholine with acyl-alkyl residue sum C44:3	Phosphatidylcholines		Level 2
PC ae C44:4	Phosphatidylcholine with acyl-alkyl residue sum C44:4	Phosphatidylcholines		Level 2
PC ae C44:5	Phosphatidylcholine with acyl-alkyl residue sum C44:5	Phosphatidylcholines		Level 2
PC ae C44:6	Phosphatidylcholine with acyl-alkyl residue sum C44:6	Phosphatidylcholines		Level 2
SM (OH) C14:1	Hydroxysphingomyelin with acyl residue sum C14:1	Sphingomyelins		Level 2
SM (OH) C16:1	Hydroxysphingomyelin with acyl residue sum C16:1	Sphingomyelins		Level 2
SM (OH) C22:1	Hydroxysphingomyelin with acyl residue sum C22:1	Sphingomyelins		Level 2
SM (OH) C22:2	Hydroxysphingomyelin with acyl residue sum C22:2	Sphingomyelins		Level 2
SM (OH) C24:1	Hydroxysphingomyelin with acyl residue sum C24:1	Sphingomyelins		Level 2
SM C16:0	C16:0 Sphingomyelin	Sphingomyelins		Level 2
SM C16:1	Sphingomyelin with acyl residue sum C16:1	Sphingomyelins		Level 2
SM C18:0	C18:0 Sphingomyelin	Sphingomyelins		Level 2

Metabolomics

Abbreviation	Name	Metabolite class	HMDB ID	MSI level
SM C18:1	C18:1;11Z-enoyl Sphingomyelin	Sphingomyelins	HMDB12100	Level 2
SM C18:1	C18:1;9Z-enoyl Sphingomyelin	Sphingomyelins	HMDB12101	Level 2
SM C20:2	Sphingomyelin with acyl residue sum C20:2	Sphingomyelins		Level 2
SM C22:3	Sphingomyelin with acyl residue sum C22:3	Sphingomyelins		Level 2
SM C24:0	C24:0 Sphingomyelin	Sphingomyelins		Level 2
SM C24:1	SM(d18:1/24:1(15Z))	Sphingomyelins	HMDB12107	Level 2
SM C26:0	C26:0 Sphingomyelin	Sphingomyelins		Level 2
SM C26:1	SM(d18:0/26:1(17Z))	Sphingomyelins	HMDB13461	Level 2
H1	Aldohexose	Hexoses		Level 2
H1	D-Galactose	Hexoses	HMDB00143	Level 2
H1	Alpha-D-Glucose	Hexoses	HMDB03345	Level 2
H1	alpha-D-Mannopyranose	Hexoses		Level 2
H1	Alpha-L-Galactopyranose	Hexoses		Level 2
H1	Beta-D-Galactose	Hexoses	HMDB03449	Level 2
H1	Beta-D-Glucose	Hexoses	HMDB00516	Level 2
H1	D-Allopyranose	Hexoses		Level 2
H1	D-Allose	Hexoses		Level 2
H1	D-Altropyranose	Hexoses		Level 2
H1	D-Altrose	Hexoses		Level 2
H1	D-Erythrohexulose	Hexoses		Level 2
H1	D-Fructose	Hexoses	HMDB00660	Level 2
H1	D-Fructopyranose	Hexoses		Level 2
H1	D-Fructose	Hexoses		Level 2
H1	D-Galactose	Hexoses		Level 2
H1	D-Glucose	Hexoses	HMDB00122	Level 2
H1	D-Gulopyranose	Hexoses		Level 2
H1	D-Gulose	Hexoses		Level 2
H1	D-Idopyranose	Hexoses		Level 2
H1	D-Idose	Hexoses		Level 2
H1	D-Mannose	Hexoses	HMDB00169	Level 2
H1	D-Psicopyranose	Hexoses		Level 2
H1	D-Psicose	Hexoses		Level 2
H1	D-Sorbopyranose	Hexoses		Level 2
H1	D-Sorbose	Hexoses		Level 2
H1	D-Tagatofuranose	Hexoses		Level 2
H1	D-Tagatopyranose	Hexoses		Level 2
H1	D-Tagatose	Hexoses	HMDB03418	Level 2
H1	D-Talopyranose	Hexoses		Level 2
H1	D-Talose	Hexoses		Level 2
H1	Gulose	Hexoses		Level 2
H1	Ketohexose	Hexoses		Level 2
H1	L-Allopyranose	Hexoses		Level 2

Metabolomics

Abbreviation	Name	Metabolite class	HMDB ID	MSI level
H1	L-Allose	Hexoses		Level 2
H1	L-Altrose	Hexoses		Level 2
H1	L-Fructofuranose	Hexoses		Level 2
H1	L-Fructopyranose	Hexoses		Level 2
H1	L-Galactose	Hexoses		Level 2
H1	L-Glucose	Hexoses		Level 2
H1	L-Gulose	Hexoses	HMDB12326	Level 2
H1	L-Gulose	Hexoses		Level 2
H1	L-Idose	Hexoses		Level 2
H1	L-Mannopyranose	Hexoses		Level 2
H1	L-Mannose	Hexoses		Level 2
H1	L-Sorbopyranose	Hexoses		Level 2
H1	L-Sorbose	Hexoses		Level 2
H1	L-Talose	Hexoses		Level 2

Table S-2 Tissue metabolite concentrations at optimal extraction conditions.
The table lists the mean (± standard deviation) tissue metabolite concentrations in pmol/mg tissue for each murine tissue type measured at the optimal extraction condition (summarized in Table 2 of the manuscript). The values were rounded to the first decimal. Metabolite abbreviations are explained in Table S-1. Metabolite concentrations below the LOD or higher than the ULOQ are indicated by '<LOD' or '>ULOQ', respectively. Abbreviations for extraction solvents are as follows: MeOH, 100% methanol; EtOH/PB, 85/15 (v/v) mixture of ethanol and 10 mM phosphate buffer pH 7.5. Tissue to solvent ratios are denoted as 1:X, indicating 1 mg of tissue was homogenized with X µL solvent.

Metabolite abbreviation	Liver EtOH/PB 1:3	Kidney MeOH 1:3	Skeletal muscle EtOH/PB 1:3	Fat EtOH/PB 1:6	Brain EtOH/PB 1:6	Pituitary gland MeOH 1:12	Lung EtOH/PB 1:3	Bone EtOH/PB 1:6	Adrenal gland EtOH/PB 1:12	Testis MeOH 1:6	Ovary EtOH/PB 1:3
C0	203.5±7.9	329.5±5.9	129.0±4.3	144.3±3.3	86.1±1.8	123.3±6.2	582.0±25.2	132.0±3.0	233.0±8.4	149.8±5.3	143.0±4.1
C2	115.5±4.7	91.1±2.5	72.2±0.5	32.1±1.3	32.4±0.8	39.5±1.0	289.0±7.7	125.5±2.5	41.0±0.5	102.8±5.9	115.7±6.6
C3	8.6±0.3	12.1±0.5	1.7±0.0	2.8±0.0	1.8±0.1	2.3±0.1	6.1±0.4	4.8±0.2	5.9±0.3	3.7±0.2	5.5±0.3
C3-DC (C4-OH)	5.7±0.1	2.6±0.1	2.2±0.0	0.9±0.1	<LOD	<LOD	4.1±0.1	1.5±0.0	<LOD	0.9±0.1	2.2±0.1
C3-OH	<LOD	0.7±0.0	<LOD	<LOD	<LOD	2.0±0.0	<LOD	0.7±0.4	<LOD	1.0±0.1	<LOD
C3:1	<LOD	<LOD	<LOD	<LOD	<LOD	<LOD	<LOD	<LOD	<LOD	<LOD	<LOD
C4	13.9±0.3	8.0±0.3	6.8±0.2	3.6±0.0	1.2±0.0	2.9±0.0	7.5±0.4	7.1±0.4	3.3±0.1	5.9±0.3	15.2±0.6
C4:1	0.3±0.01	<LOD	<LOD	<LOD	<LOD	<LOD	0.1±0.1	<LOD	<LOD	<LOD	0.1±0.1
C5	1.4±0.1	1.7±0.1	0.6±0.0	0.5±0.0	0.4±0.0	0.7±0.0	1.0±0.1	2.5±0.1	1.1±0.1	0.6±0.0	3.3±0.2
C5-DC (C6-OH)	1.2±0.1	0.5±0.0	0.3±0.0	<LOD	<LOD	<LOD	0.4±0.0	<LOD	<LOD	<LOD	0.3±0.0
C5-M-DC	1.0±0.1	0.3±0.0	<LOD	<LOD	<LOD	<LOD	<LOD	<LOD	<LOD	<LOD	<LOD
C5-OH (C3-DC-M)	0.7±0.0	1.4±0.1	0.8±0.0	<LOD	<LOD	<LOD	0.9±0.1	<LOD	<LOD	<LOD	0.6±0.1
C5:1	<LOD	<LOD	0.2±0.1	<LOD	<LOD	<LOD	<LOD	<LOD	<LOD	<LOD	<LOD
C5:1-DC	0.1±0.0	0.1±0.0	<LOD	<LOD	<LOD	<LOD	0.1±0.0	<LOD	<LOD	<LOD	0.01±0.0
C6 (C4:1-DC)	1.7±0.1	1.4±0.1	2.0±0.1	<LOD	<LOD	<LOD	2.6±0.2	2.3±0.0	<LOD	1.0±0.1	1.3±0.1
C6:1	<LOD	0.2±0.0	<LOD	<LOD	<LOD	<LOD	0.2±0.0	<LOD	<LOD	<LOD	0.2±0.1
C7-DC	0.6±0.0	0.2±0.0	0.2±0.0	<LOD	<LOD	<LOD	0.3±0.0	<LOD	<LOD	<LOD	0.2±0.0
C8	<LOD	1.0±0.0	1.1±0.0	<LOD	<LOD	<LOD	0.8±0.0	<LOD	<LOD	<LOD	0.9±0.0
C9	<LOD	<LOD	<LOD	<LOD	<LOD	<LOD	<LOD	<LOD	<LOD	<LOD	<LOD
C10	<LOD	0.8±0.0	0.9±0.0	<LOD	<LOD	<LOD	<LOD	<LOD	<LOD	<LOD	<LOD
C10:1	<LOD	<LOD	<LOD	<LOD	<LOD	<LOD	<LOD	<LOD	<LOD	<LOD	<LOD
C10:2	<LOD	0.2±0.1	<LOD	<LOD	<LOD	<LOD	0.2±0.1	<LOD	0.5±0.2	<LOD	0.2±0.0
C12	0.2±0.1	0.9±0.0	1.5±0.0	<LOD	<LOD	<LOD	1.0±0.1	0.8±0.4	<LOD	0.4±0.2	0.6±0.0
C12-DC	<LOD	<LOD	<LOD	<LOD	<LOD	<LOD	<LOD	<LOD	<LOD	<LOD	<LOD
C12:1	<LOD	<LOD	<LOD	<LOD	<LOD	<LOD	<LOD	<LOD	<LOD	<LOD	<LOD
C14	0.4±0.0	2.1±0.1	4.5±0.1	0.8±0.0	0.6±0.0	3.4±0.1	3.4±0.1	4.2±0.1	0.8±0.1	0.9±0.1	1.6±0.1
C14:1	0.1±0.0	0.6±0.0	1.8±0.0	0.1±0.1	0.1±0.1	<LOD	0.9±0.0	1.4±0.1	<LOD	0.2±0.0	0.3±0.0
C14:1-OH	0.1±0.0	0.2±0.0	0.3±0.0	<LOD	<LOD	<LOD	0.5±0.0	0.3±0.0	<LOD	<LOD	0.1±0.0
C14:2	0.1±0.0	0.2±0.0	0.4±0.0	<LOD	<LOD	<LOD	0.2±0.0	0.3±0.0	<LOD	<LOD	0.1±0.0
C14:2-OH	<LOD	0.1±0.0	0.1±0.0	<LOD	<LOD	<LOD	0.2±0.0	<LOD	<LOD	<LOD	0.1±0.0
C16	2.1±0.1	7.9±0.2	24.3±0.8	2.6±0.1	1.5±0.0	4.0±0.2	9.5±0.3	20.8±0.2	3.7±0.1	4.4±0.2	7.5±0.4

Metabolomics

Metabolite abbreviation	Liver EtOH/PB 1:3	Kidney MeOH 1:3	Skeletal muscle EtOH/PB 1:3	Fat EtOH/PB 1:6	Brain EtOH/PB 1:6	Pituitary gland MeOH 1:12	Lung EtOH/PB 1:3	Bone EtOH/PB 1:6	Adrenal gland EtOH/PB 1:12	Testis MeOH 1:6	Ovary EtOH/PB 1:3
C16-OH	0.1±0.0	0.4±0.0	0.4±0.0	<LOD	<LOD	0.3±0.0	0.9±0.0	0.3±0.0	0.2±0.0	0.2±0.0	0.3±0.0
C16:1	0.6±0.0	1.7±0.1	6.4±0.2	0.4±0.2	<LOD	<LOD	1.6±0.1	3.9±0.2	<LOD	0.6±0.0	0.8±0.0
C16:1-OH	0.2±0.0	0.3±0.0	0.5±0.0	0.2±0.1	<LOD	0.3±0.1	0.6±0.0	0.4±0.0	<LOD	<LOD	0.3±0.0
C16:2	0.1±0.0	0.4±0.0	1.2±0.0	0.1±0.0	<LOD	<LOD	0.3±0.0	0.8±0.1	0.1±0.1	0.1±0.0	0.2±0.0
C16:2-OH	0.1±0.1	0.1±0.0	0.2±0.0	<LOD	<LOD	<LOD	0.2±0.0	<LOD	<LOD	<LOD	<LOD
C18	1.5±0.1	2.5±0.1	4.7±0.1	0.6±0.0	0.7±0.0	2.3±0.1	1.4±0.0	3.4±0.2	1.4±0.1	1.1±0.1	3.8±0.2
C18:1	2.5±0.1	4.7±0.1	19.2±0.1	1.5±0.1	1.3±0.0	1.4±0.1	4.6±0.1	25.0±0.1	2.3±0.1	2.0±0.1	4.1±0.2
C18:1-OH	0.3±0.0	0.4±0.0	0.7±0.0	0.2±0.1	<LOD	0.4±0.0	0.9±0.0	0.7±0.1	0.5±0.0	0.2±0.0	0.4±0.0
C18:2	0.7±0.0	3.3±0.2	12.1±0.2	0.5±0.0	0.2±0.0	0.4±0.0	1.6±0.1	16.4±0.3	0.8±0.0	0.7±0.0	1.5±0.1
Ala	3680.0±475.1	781.0±49.6	1613.3±178.3	330.8±21.8	737.3±18.4	969.3±41.9	888.0±95.7	853.5±24.5	922.5±38.1	1131.2±26.4	1393.0±95.2
Arg	<LOD	60.4±5.0	74.1±9.1	50.5±4.0	75.6±18.0	92.0±5.2	67.5±10.3	24.0±5.2	72.8±7.9	35.6±4.9	94.1±13.3
Asn	113.9±14.3	95.9±8.2	85.3±5.8	34.6±3.4	91.0±1.1	133.5±6.3	88.8±13.0	<LOD	60.0±2.8	152.0±7.1	108.5±23.1
Asp	247.0±11.0	607.0±23.7	358.0±44.8	222.8±14.0	<LOD	1240.0±92.9	2074.0±1020.5	<LOD	870.5±113.5	539.2±27.3	1021.0±178.8
Cit	38.6±3.5	17.3±0.7	78.8±5.6	46.3±2.6	19.2±1.8	14.7±7.4	63.8±7.5	32.2±10.4	67.6±3.7	7.8±3.1	65.6±6.2
Gln	2371.0±427.8	914.0±89.3	1430.7±103.1	826.0±46.7	5266.3±499.4	2460.3±105.0	1809.6±255.0	325.5±17.5	928.8±89.3	2206.4±190.0	2080.7±198.5
Glu	1433.0±195.9	3067.0±178.8	860.0±45.3	798.0±27.6	<LOD	3129.8±112.0	<LOD	68.9±4.0	2255.5±120.0	3339.0±149.1	3109.0±1465.6
Gly	2042.0±259.4	2406.0±180.6	2562.7±165.0	637.7±37.4	1082.7±63.0	1092.5±36.2	4452.0±700.6	176.0±46.0	694.0±42.0	2903.6±181.5	2034.3±194.2
His	412.5±36.3	97.7±11.6	185.3±64.9	47.8±2.2	104.7±8.3	86.6±5.5	92.4±26.0	28.6±6.5	94.3±28.5	73.0±6.5	139.7±10.2
Ile	307.5±24.7	140.3±13.0	97.7±4.4	61.0±3.0	55.2±2.8	189.3±8.6	112.2±15.7	152.0±1.0	121.3±7.5	44.0±4.9	170.3±15.2
Leu	535.0±31.5	247.8±20.8	164.0±15.6	112.5±8.7	123.0±13.4	474.3±24.5	212.4±36.6	280.5±10.5	241.3±25.0	79.3±9.8	332.0±19.1
Lys	276.5±39.7	119.2±14.8	183.0±9.4	104.9±8.3	127.7±11.8	163.3±9.9	111.36±22.4	<LOD	141.3±15.2	121.0±13.4	167.7±18.1
Met	48.1±4.9	57.3±7.5	46.4±1.4	27.7±0.9	41.4±5.7	93.9±6.4	47.3±7.6	65.3±8.9	57.6±7.3	36.4±3.0	100.2±8.2
Orn	168.3±19.7	20.4±3.0	23.3±1.9	10.8±0.7	7.5±0.0	<LOD	21.5±8.8	<LOD	18.7±1.1	<LOD	20.1±1.9
Phe	174.0±19.0	99.6±8.4	88.5±6.1	41.0±2.0	66.1±4.1	184.8±7.7	66.3±8.0	83.1±6.7	94.3±8.4	84.3±6.5	151.0±10.7
Pro	358.3±9.0	128.5±5.5	234.3±5.8	73.8±2.9	123.0±10.4	357.8±18.7	105.4±8.4	175.5±5.5	127.3±10.7	351.2±19.2	497.7±22.5
Ser	159.0±25.0	247.0±22.9	250.0±31.0	121.8±7.5	791.0±50.8	271.5±9.2	253.0±43.3	<LOD	181.3±19.5	352.0±27.4	344.7±18.8
Thr	221.0±21.1	179.8±8.8	168.0±23.2	125.8±2.7	293.0±6.4	235.0±7.4	228.0±35.9	96.2±8.8	208.5±10.4	230.8±7.7	454.3±24.2
Trp	62.0±5.9	51.2±4.7	46.4±4.3	30.6±1.9	27.6±2.7	48.5±2.2	44.1±5.5	30.5±2.0	59.9±3.3	35.4±3.6	85.5±7.2
Tyr	190.3±18.7	130.5±14.2	110.0±8.6	42.2±2.2	86.6±5.9	157.0±5.6	64.2±9.4	128.5±2.5	132.8±7.3	109.0±7.4	163.7±12.2
Val	746.0±101.3	243.0±27.2	234.7±22.2	123.5±6.7	140.7±13.2	342.3±15.3	214.2±27.1	303.5±16.5	260.8±17.1	114.4±11.0	381.0±41.1
Ac-Orn	14.8±2.7	5.8±0.8	13.4±0.3	4.2±0.3	14.2±1.4	<LOD	12.1±2.0	9.0±1.1	8.2±0.3	1.8±0.9	14.1±2.7
ADMA	0.9±0.2	0.5±0.2	0.6±0.3	0.9±0.5	<LOD	<LOD	0.9±0.5	2.4±0.6	<LOD	<LOD	1.3±0.4
alpha-AAA	91.5±11.9	15.3±1.6	4.3±0.7	17.5±1.5	47.5±9.0	9.1±1.7	38.9±5.9	<LOD	9.5±2.1	<LOD	37.2±7.1
Carnosine	2.2±0.8	7.5±0.8	444.3±118.7	3.4±0.2	64.7±22.9	<LOD	3.6±1.3	44.1±0.9	8.1±0.6	1.0±0.4	2.6±0.4
Creatinine	10.1±0.8	15.7±3.9	190.0±12.8	11.2±0.4	81.4±3.5	<LOD	13.4±1.1	83.2±5.2	23.6±2.0	246.6±12.5	17.9±0.9
DOPA	<LOD	1.2±0.5	<LOD	<LOD	<LOD	<LOD	<LOD	<LOD	<LOD	2.1±0.8	<LOD
Dopamine	2.0±0.0	2.0±0.1	<LOD	<LOD	16.7±0.6	6.7±0.4	2.0±0.8	<LOD	56.3±2.2	<LOD	2.0±0.9
Histamine	0.2±0.1	1.2±0.1	11.3±3.4	7.3±2.0	0.2±0.1	2.5±0.1	7.8±2.5	23.8±0.3	22.8±10.3	1.4±0.1	14.7±0.9
Kynurenine	4.6±0.7	<LOD	1.5±0.1	3.8±0.9	2.2±1.1	<LOD	2.3±0.5	6.2±3.1	5.0±2.2	<LOD	2.9±0.3
Met-SO	<LOD	<LOD	1.9±0.4	<LOD	<LOD	<LOD	6.7±1.1	4.8±2.4	<LOD	<LOD	2.4±0.3
Nitro-Tyr	<LOD	<LOD	<LOD	<LOD	<LOD	<LOD	<LOD	<LOD	<LOD	<LOD	<LOD
OH-Pro	<LOD	<LOD	<LOD	<LOD	<LOD	<LOD	<LOD	<LOD	<LOD	<LOD	<LOD
PEA	0.1±0.0	0.1±0.0	<LOD	<LOD	<LOD	0.3±0.0	<LOD	0.4±0.2	0.4±0.2	<LOD	<LOD

Metabolomics

Metabolite abbreviation	Liver<br><br>EtOH/PB 1:3	Kidney<br><br>MeOH 1:3	Skeletal muscle<br>EtOH/PB 1:3	Fat<br><br>EtOH/PB 1:6	Brain<br><br>EtOH/PB 1:6	Pituitary gland<br>MeOH 1:12	Lung<br><br>EtOH/PB 1:3	Bone<br><br>EtOH/PB 1:6	Adrenal gland<br>EtOH/PB 1:12	Testis<br><br>MeOH 1:6	Ovary<br><br>EtOH/PB 1:3
Putrescine	4.6±0.4	6.5±0.3	1.3±0.2	1.4±0.1	2.0±0.1	15.9±0.6	4.5±0.4	4.8±0.4	2.5±0.3	5.7±0.1	8.3±0.3
Sarcosine	<LOD	488.0±59.9	18.4±2.3	9.2±1.1	84.7±3.6	<LOD	38.6±3.8	9.9±1.2	15.1±2.2	1568.0±627.2	18.5±1.6
SDMA	<LOD	<LOD	<LOD	<LOD	<LOD	<LOD	<LOD	<LOD	<LOD	<LOD	<LOD
Serotonin	2.3±0.2	2.2±0.1	0.6±0.0	1.9±0.1	4.2±0.2	2.6±0.2	55.2±28.4	4.4±0.4	7.1±0.3	0.4±0.0	4.4±0.3
Spermidine	50.2±3.8	16.9±1.3	5.3±0.1	7.8±0.3	20.7±1.0	20.1±8.1	40.7±4.6	4.5±0.3	15.2±0.8	12.4±1.5	40.8±1.7
Spermine	7.2±0.6	11.0±1.5	3.3±0.3	<LOD	6.6±0.3	21.9±10.2	8.5±0.6	2.5±1.3	5.4±2.3	6.7±2.1	5.5±0.1
Taurine	>ULOQ	>ULOQ	>ULOQ	733.5±371.7	>ULOQ	>ULOQ	>ULOQ	4755.0±1667.0	>ULOQ	>ULOQ	>ULOQ
total DMA	1.4±0.6	<LOD	<LOD	<LOD	<LOD	<LOD	<LOD	6.1±3.1	<LOD	<LOD	1.4±0.1
lysoPC a C14:0	<LOD	<LOD	<LOD	<LOD	<LOD	<LOD	<LOD	<LOD	<LOD	<LOD	<LOD
lysoPC a C16:0	208.5±9.0	216.0±4.3	34.8±0.7	56.4±3.5	58.6±2.0	301.3±11.2	210.0±11.4	126.0±2.0	236.3±4.8	61.5±2.6	73.9±5.4
lysoPC a C16:1	10.8±0.5	4.1±0.2	1.1±0.0	1.5±0.1	1.9±0.1	3.0±0.3	8.4±0.5	2.2±0.1	3.7±0.2	1.0±0.1	1.1±0.1
lysoPC a C17:0	3.9±0.2	3.4±0.3	0.7±0.1	1.1±0.1	0.5±0.0	3.6±0.6	4.7±0.3	2.4±0.0	3.5±0.3	0.5±0.0	1.7±0.1
lysoPC a C18:0	59.7±2.5	63.7±1.5	12.2±0.2	39.0±0.6	13.9±0.6	102.6±5.1	96.6±3.7	77.0±0.9	103.1±4.0	16.7±0.9	48.1±2.4
lysoPC a C18:1	58.2±1.6	33.4±0.8	5.4±0.2	12.2±0.5	33.8±2.0	33.7±1.6	38.6±1.8	18.6±0.4	42.8±2.1	10.1±0.4	17.4±1.0
lysoPC a C18:2	72.2±2.4	53.5±1.0	6.5±0.2	33.0±1.5	1.6±0.1	19.2±1.0	48.3±2.5	38.4±0.9	51.6±2.0	5.5±0.4	28.1±1.9
lysoPC a C20:3	6.5±0.3	3.2±0.2	<LOD	1.4±0.6	<LOD	2.7±1.2	4.1±0.2	<LOD	3.8±0.2	<LOD	1.7±0.1
lysoPC a C20:4	41.8±1.5	18.4±0.7	4.7±0.2	8.3±0.2	13.2±0.6	16.1±0.9	24.8±1.4	13.2±0.3	51.3±2.0	6.6±0.3	15.1±0.8
lysoPC a C24:0	<LOD	<LOD	<LOD	<LOD	<LOD	<LOD	<LOD	<LOD	<LOD	<LOD	<LOD
lysoPC a C26:0	2.0±0.8	<LOD	2.1±1.0	<LOD	<LOD	<LOD	<LOD	<LOD	<LOD	<LOD	<LOD
lysoPC a C26:1	<LOD	<LOD	<LOD	<LOD	<LOD	<LOD	<LOD	<LOD	<LOD	<LOD	<LOD
lysoPC a C28:0	1.2±0.5	<LOD	<LOD	<LOD	<LOD	<LOD	<LOD	<LOD	<LOD	2.1±0.9	<LOD
lysoPC a C28:1	1.3±0.2	0.8±0.1	1.0±0.4	<LOD	1.4±0.7	2.7±1.2	0.7±0.1	<LOD	2.7±0.3	1.4±0.4	0.9±0.2
PCaa C24:0	0.6±0.2	<LOD	0.7±0.3	<LOD	0.8±0.4	1.5±0.7	0.5±0.2	<LOD	1.7±0.1	0.9±0.4	0.5±0.1
PCaa C26:0	6.1±2.6	<LOD	<LOD	<LOD	<LOD	<LOD	<LOD	<LOD	<LOD	<LOD	<LOD
PCaa C28:1	1.4±0.4	0.9±0.1	0.8±0.3	0.6±0.0	0.8±0.1	2.0±0.2	1.0±0.0	1.1±0.1	1.3±0.1	2.0±0.2	0.9±0.0
PCaa C30:0	7.5±0.3	6.3±0.3	51.1±0.3	10.0±0.3	51.5±1.6	81.9±2.6	197.6±7.7	82.1±1.4	25.0±0.9	16.4±1.0	30.2±0.2
PCaa C30:2	0.8±0.2	<LOD	0.8±0.1	0.1±0.0	0.2±0.1	0.7±0.2	0.8±0.2	0.8±0.2	1.1±0.2	0.2±0.2	0.2±0.1
PCaa C32:0	94.5±5.2	97.5±5.2	150.3±3.9	152.8±2.3	1209.3±37.3	1392.8±42.7	604.0±10.1	756.0±19.0	501.8±14.9	64.2±3.8	291.7±13.2
PCaa C32:1	136.8±7.4	23.4±1.2	177.7±3.1	31.1±1.9	219.7±9.2	198.0±7.0	633.2±15.3	191.0±5.0	93.0±3.6	81.5±3.3	63.7±0.7
PCaa C32:2	33.0±1.7	4.1±0.2	29.6±0.2	3.7±0.1	1.1±0.4	10.0±0.4	54.4±2.7	27.6±0.5	11.1±0.3	2.5±0.1	5.3±0.2
PCaa C32:3	2.2±0.1	0.6±0.1	5.6±0.1	0.6±0.0	0.5±0.1	1.4±0.2	9.5±0.5	5.5±0.1	1.2±0.1	0.4±0.1	0.9±0.0
PCaa C34:1	603.8±36.5	132.3±5.4	476.3±9.0	216.8±3.9	1582.0±53.7	1787.8±57.4	433.2±15.1	914.0±21.0	1265.5±46.9	976.2±53.1	666.7±32.5
PCaa C34:2	894.8±62.2	269.3±12.1	571.7±6.8	452.5±9.8	176.3±8.1	588.0±23.9	450.2±15.9	762.5±14.5	1051.8±26.2	242.4±10.7	420.0±15.6
PCaa C34:3	259.5±12.7	20.2±0.9	59.2±1.0	22.2±0.5	5.9±0.4	31.9±1.2	42.8±2.2	68.2±1.8	56.4±1.9	12.8±0.4	20.5±0.9
PCaa C34:4	21.4±0.9	2.6±0.2	31.3±0.2	1.3±0.0	1.8±0.1	6.7±0.1	6.8±0.2	18.5±0.2	4.6±0.2	5.9±0.4	3.5±0.2
PCaa C36:0	4.0±0.4	22.2±1.3	24.7±0.5	4.6±0.3	25.3±2.0	10.8±1.4	13.7±0.6	20.5±1.1	16.9±1.6	24.7±1.5	11.8±0.1
PCaa C36:1	48.3±2.5	18.4±0.7	28.2±0.4	138.0±4.7	886.7±35.3	487.0±15.4	67.3±1.5	132.5±4.5	458.5±9.8	129.4±5.0	279.3±9.2
PCaa C36:2	393.3±19.5	88.7±3.9	118.0±2.2	694.5±12.3	427.3±15.8	502.5±21.0	156.6±6.1	432.0±4.0	1105.0±37.4	196.8±9.7	471.3±9.0
PCaa C36:3	559.3±28.9	94.0±4.8	273.7±6.0	196.5±2.3	93.7±4.8	292.0±17.8	114.6±5.6	315.5±6.5	561.8±17.7	288.6±11.5	202.0±8.5
PCaa C36:4	867.0±56.6	368.5±11.6	784.3±28.0	235.3±2.8	871.3±25.5	1989.3±58.8	424.4±14.1	1188.0±45.0	2090.3±88.1	1107.2±62.1	651.0±9.8
PCaa C36:5	145.0±8.4	15.8±0.6	99.0±1.5	9.1±0.2	14.8±0.9	34.7±0.9	23.9±0.8	51.6±1.3	40.5±1.7	20.9±0.8	18.5±0.7
PCaa C36:6	8.0±0.5	7.1±0.3	14.0±0.3	0.4±0.0	2.2±0.1	2.2±0.2	2.0±0.1	7.6±0.1	2.5±0.1	2.9±0.2	1.2±0.1
PCaa C38:0	4.7±0.2	32.6±1.4	4.3±0.0	2.2±0.1	5.1±0.3	5.8±0.3	3.4±0.1	17.8±0.5	7.6±0.3	9.2±0.2	10.9±0.1

Metabolomics

**Metabolite abbreviation**	**Liver EtOH/PB 1:3**	**Kidney MeOH 1:3**	**Skeletal muscle EtOH/PB 1:3**	**Fat EtOH/PB 1:6**	**Brain EtOH/PB 1:6**	**Pituitary gland MeOH 1:12**	**Lung EtOH/PB 1:3**	**Bone EtOH/PB 1:6**	**Adrenal gland EtOH/PB 1:12**	**Testis MeOH 1:6**	**Ovary EtOH/PB 1:3**
PCaa C38:1	1.6±0.3	22.7±0.8	1.7±0.1	1.8±0.8	26.6±0.8	9.1±0.6	2.5±0.2	5.8±1.2	5.9±0.7	1.4±0.2	3.8±0.3
PCaa C38:3	60.7±1.6	16.0±0.5	16.5±0.3	44.0±2.0	38.3±2.4	149.0±6.0	41.0±1.2	107.0±3.0	330.3±12.5	71.4±4.1	91.5±1.4
PCaa C38:4	346.5±17.7	120.0±5.2	219.3±2.9	210.3±5.1	718.7±26.2	1775.8±59.8	212.2±9.8	533.0±19.0	2186.8±83.8	513.0±24.3	611.7±4.9
PCaa C38:5	442.3±19.4	132.0±6.7	383.0±7.5	66.1±0.6	319.3±7.3	500.5±14.8	97.6±3.5	319.5±14.5	650.5±18.0	1252.6±76.9	196.3±5.7
PCaa C38:6	572.3±37.5	360.0±12.8	600.0±8.6	41.3±0.4	668.7±22.5	364.0±12.7	129.4±5.4	473.0±10.0	210.3±7.9	346.2±17.3	165.7±10.1
PCaa C40:1	<LOD	<LOD	<LOD	<LOD	8.5±0.3	<LOD	<LOD	<LOD	<LOD	<LOD	1.8±0.8
PCaa C40:2	0.8±0.1	0.8±0.1	0.6±0.1	0.6±0.1	8.8±0.6	3.5±0.6	1.4±0.1	2.8±0.0	4.0±0.2	1.6±0.1	2.8±0.1
PCaa C40:3	1.5±0.1	1.1±0.1	0.9±0.1	0.9±0.1	1.7±0.1	7.8±0.4	1.8±0.2	5.5±0.4	5.7±0.4	5.7±0.5	3.8±0.0
PCaa C40:4	6.3±0.4	2.9±0.2	7.0±0.2	6.4±0.4	48.3±2.4	116.0±4.3	14.3±0.4	33.2±0.8	85.0±2.3	41.7±1.7	56.2±2.0
PCaa C40:5	23.7±0.9	14.3±0.3	34.1±1.1	12.5±0.5	44.7±2.1	137.0±4.6	18.6±1.4	72.0±1.4	204.3±7.7	182.8±7.8	78.8±2.7
PCaa C40:6	91.8±5.3	47.2±1.9	89.2±2.4	26.1±0.6	327.3±13.0	157.8±6.7	39.6±1.9	141.5±2.5	209.0±6.3	159.0±6.4	98.6±3.1
PCaa C42:0	0.4±0.1	0.2±0.0	0.4±0.2	<LOD	0.6±0.3	0.8±0.3	0.2±0.1	0.9±0.1	1.1±0.1	0.6±0.1	0.3±0.0
PCaa C42:1	0.3±0.1	0.2±0.0	0.4±0.1	0.2±0.0	4.1±0.3	0.6±0.1	0.3±0.0	1.0±0.1	0.7±0.1	0.5±0.2	0.3±0.0
PCaa C42:2	0.5±0.1	0.2±0.1	0.5±0.2	0.2±0.0	4.2±0.2	0.7±0.1	0.6±0.0	1.0±0.1	1.1±0.1	0.7±0.1	1.1±0.0
PCaa C42:4	0.4±0.0	0.5±0.1	0.5±0.0	0.5±0.0	3.6±0.2	3.9±0.2	1.1±0.1	2.1±0.0	2.3±0.1	4.0±0.2	2.0±0.1
PCaa C42:5	0.9±0.1	1.9±0.1	0.9±0.1	0.7±0.1	3.5±0.2	8.5±0.3	1.8±0.1	6.8±0.1	7.4±0.5	15.7±0.7	4.6±0.1
PCaa C42:6	2.6±0.1	1.4±0.0	2.4±0.3	2.2±1.0	4.1±0.2	9.3±0.2	2.5±0.1	9.1±1.1	13.0±0.5	14.3±0.4	5.4±0.2
PCae C30:0	0.8±0.1	1.0±0.1	1.5±0.1	<LOD	1.8±0.1	3.1±0.4	3.9±0.2	11.2±0.4	<LOD	6.8±0.4	1.1±0.1
PCae C30:1	0.9±0.4	0.2±0.1	1.5±0.4	0.3±0.1	0.6±0.1	1.0±0.3	1.6±0.1	1.1±0.1	0.7±0.1	1.5±0.2	0.2±0.1
PCae C30:2	<LOD	<LOD	<LOD	<LOD	<LOD	<LOD	<LOD	<LOD	<LOD	<LOD	<LOD
PCae C32:1	2.8±0.2	3.5±0.2	9.5±0.1	2.2±0.1	15.1±0.8	16.5±0.9	43.9±1.7	42.1±0.4	9.3±0.5	42.3±2.3	5.2±0.3
PCae C32:2	0.9±0.2	1.0±0.1	1.8±0.2	0.4±0.0	1.0±0.1	1.6±0.1	7.3±0.4	3.7±0.2	1.3±0.0	2.9±0.2	0.8±0.1
PCae C34:0	2.6±0.2	1.5±0.1	5.1±0.3	3.4±0.1	17.4±1.0	41.4±2.7	9.0±0.2	46.7±0.6	10.4±0.3	0.8±0.2	8.4±0.6
PCae C34:1	22.6±1.3	10.8±0.6	23.9±0.6	10.4±0.5	87.9±4.1	77.1±2.0	35.4±1.8	216.5±0.5	31.7±1.1	142.0±8.0	43.5±0.9
PCae C34:2	15.9±0.8	10.9±0.4	10.4±0.2	4.9±0.1	12.1±0.4	12.8±0.5	16.9±0.6	134.5±1.5	16.2±0.8	31.1±1.4	13.3±0.5
PCae C34:3	2.1±0.1	2.8±0.2	3.8±0.1	1.5±0.0	3.5±0.1	2.9±0.1	6.4±0.3	8.2±0.2	6.8±0.1	2.3±0.2	3.1±0.1
PCae C36:0	2.2±0.3	1.0±0.1	1.7±0.2	1.2±0.1	3.8±0.6	7.9±1.4	1.9±0.1	5.7±0.3	3.7±0.1	1.1±0.1	2.3±0.3
PCae C36:1	14.8±0.9	3.1±0.1	10.3±0.4	7.5±0.4	39.2±2.1	37.2±2.1	7.7±0.5	22.6±0.2	28.7±1.1	7.9±0.3	19.4±0.5
PCae C36:2	29.0±1.0	4.4±0.2	10.1±0.2	14.4±0.4	11.0±0.5	14.3±0.4	8.8±0.4	63.7±1.1	23.1±1.1	14.9±0.7	17.5±0.6
PCae C36:3	15.2±0.7	2.9±0.2	4.2±0.1	3.1±0.2	3.9±0.2	3.7±0.1	5.5±0.2	73.1±0.3	7.4±0.3	5.2±0.2	7.3±0.1
PCae C36:4	15.3±1.0	34.9±1.5	15.8±0.3	8.6±0.1	8.7±0.2	19.5±0.8	34.1±1.9	184.5±0.5	30.8±0.3	114.6±5.0	39.2±2.2
PCae C36:5	5.1±0.3	22.7±1.5	15.4±0.4	7.4±0.2	13.8±0.7	16.2±0.7	46.7±1.5	26.0±0.5	259.5±11.5	26.6±1.0	28.8±0.7
PCae C38:0	38.8±2.5	9.8±0.3	39.3±0.1	2.3±0.1	9.8±0.5	7.9±0.7	5.1±0.2	17.6±0.2	7.7±0.4	3.8±0.2	4.1±0.2
PCae C38:1	2.9±0.2	0.9±0.0	1.6±0.0	3.0±0.9	5.2±0.3	3.9±0.5	1.4±0.1	3.8±0.1	5.1±0.7	1.2±0.1	2.9±0.2
PCae C38:2	9.4±0.3	1.4±0.1	2.1±0.2	10.4±0.6	3.7±0.1	5.0±0.4	2.4±0.2	11.0±0.2	14.0±0.2	1.8±0.1	5.5±0.3
PCae C38:3	11.2±0.5	1.7±0.2	4.4±0.6	3.5±0.2	2.1±0.3	5.2±0.8	3.0±0.2	12.7±0.9	10.0±0.8	4.0±0.4	4.5±0.1
PCae C38:4	28.0±1.8	9.9±0.5	20.0±0.2	8.4±0.1	13.9±0.8	67.6±3.2	16.6±0.9	79.4±3.1	95.3±3.1	49.9±2.2	48.6±1.8
PCae C38:5	18.2±0.9	22.9±0.9	14.7±0.4	7.5±0.3	8.2±0.3	21.0±0.7	23.6±1.4	159.5±4.5	44.8±1.3	264.0±11.7	53.7±2.3
PCae C38:6	6.8±0.3	221.0±10.9	22.5±0.7	3.0±0.1	9.9±0.4	10.9±0.7	16.5±0.7	43.9±1.1	5.9±1.4	254.2±10.8	19.4±0.8
PCae C40:1	40.8±1.7	26.9±1.1	45.4±0.5	4.7±0.1	12.3±0.7	15.3±1.1	10.0±0.4	40.5±0.8	26.2±1.0	21.6±0.6	16.3±1.0
PCae C40:2	2.6±0.1	1.2±0.0	2.5±0.3	1.1±0.0	1.5±0.1	2.2±0.4	0.9±0.1	4.4±0.1	3.7±0.2	1.0±0.1	1.2±0.1
PCae C40:3	2.4±0.2	0.6±0.1	0.8±0.2	1.1±0.1	1.1±0.1	3.3±0.5	1.1±0.0	3.8±0.4	4.5±0.3	2.2±0.1	1.8±0.1
PCae C40:4	8.1±0.2	2.2±0.0	3.4±0.2	3.4±0.1	3.7±0.2	17.8±0.9	4.0±0.2	19.3±0.2	41.6±1.8	18.6±1.2	9.9±0.3

Metabolomics

Metabolite abbreviation	Liver<br>EtOH/PB 1:3	Kidney<br>MeOH 1:3	Skeletal muscle<br>EtOH/PB 1:3	Fat<br>EtOH/PB 1:6	Brain<br>EtOH/PB 1:6	Pituitary gland<br>MeOH 1:12	Lung<br>EtOH/PB 1:3	Bone<br>EtOH/PB 1:6	Adrenal gland<br>EtOH/PB 1:12	Testis<br>MeOH 1:6	Ovary<br>EtOH/PB 1:3
PCae C40:5	7.1±0.2	2.9±0.3	5.4±0.2	2.0±0.0	3.4±0.2	12.3±0.5	4.1±0.1	19.5±0.4	21.5±0.6	50.5±2.3	12.7±0.2
PCae C40:6	9.1±0.5	8.8±0.2	8.9±0.2	1.6±0.4	8.2±0.4	9.7±0.4	3.8±0.2	22.2±0.0	9.3±0.4	26.6±1.0	12.7±0.6
PCae C42:0	3.5±0.1	1.8±0.1	1.9±0.1	<LOD	9.5±0.3	10.0±0.5	2.0±0.1	6.3±3.2	12.8±0.2	5.8±0.2	4.7±0.1
PCae C42:1	3.3±0.2	2.3±0.1	2.7±0.2	1.1±0.0	8.3±0.5	11.6±0.5	2.3±0.1	8.8±0.3	29.3±0.7	5.5±0.2	10.7±0.6
PCae C42:2	3.4±0.2	1.8±0.1	3.5±0.3	0.5±0.0	4.3±0.2	3.3±0.2	1.1±0.1	4.2±0.2	6.9±0.3	16.1±0.7	2.9±0.0
PCae C42:3	7.7±0.5	3.8±0.2	5.3±0.3	1.0±0.1	15.2±0.8	4.4±0.2	2.9±0.1	7.0±0.1	4.3±0.1	7.3±0.2	5.3±0.2
PCae C42:4	<LOD	<LOD	<LOD	<LOD	<LOD	<LOD	<LOD	<LOD	<LOD	<LOD	<LOD
PCae C42:5	<LOD	<LOD	<LOD	<LOD	<LOD	<LOD	<LOD	<LOD	<LOD	<LOD	<LOD
PCae C44:3	0.5±0.0	0.3±0.0	2.4±0.1	0.2±0.1	2.9±0.2	1.3±0.1	0.4±0.0	1.7±0.2	3.4±0.1	7.8±0.4	1.9±0.1
PCae C44:4	0.4±0.1	0.3±0.0	0.9±0.0	0.2±0.0	1.4±0.1	0.6±0.1	0.3±0.0	1.3±0.2	1.0±0.1	3.6±0.2	0.6±0.1
PCae C44:5	0.7±0.1	0.4±0.1	0.5±0.1	0.2±0.0	7.7±0.6	0.8±0.1	0.5±0.0	2.0±0.1	1.5±0.2	1.6±0.1	0.9±0.0
PCae C44:6	0.8±0.0	<LOD	<LOD	<LOD	<LOD	<LOD	<LOD	2.8±0.1	1.4±0.6	0.8±0.0	0.4±0.2
SM (OH) C14:1	4.4±0.1	6.2±0.2	1.1±0.0	2.5±0.3	0.2±0.1	7.2±0.6	7.2±0.3	6.4±0.2	7.9±0.3	4.3±0.2	7.0±0.4
SM (OH) C16:1	1.1±0.1	1.6±0.1	2.8±0.2	2.3±0.1	2.1±0.2	12.9±0.6	3.3±0.1	5.5±0.5	6.4±0.3	2.1±0.1	7.1±0.2
SM (OH) C22:1	11.9±0.4	2.2±0.1	2.8±0.2	37.5±3.0	1.2±0.1	18.0±1.0	3.8±0.2	12.3±0.8	71.0±1.4	1.4±0.2	10.9±1.3
SM (OH) C22:2	5.0 ±0.3	1.9±0.1	0.9±0.1	10.2±0.3	1.4±0.1	5.5±0.3	4.7±0.3	5.8±0.1	17.2±0.6	1.5±0.3	5.9±0.5
SM (OH) C24:1	0.9±0.0	0.5±0.0	0.4±0.0	2.8±0.1	0.3±0.1	1.7±0.2	0.8±0.1	2.1±0.3	11.2±0.5	0.3±0.1	1.3±0.1
SM C16:0	80.1±1.5	211.0±3.2	52.4±0.2	152.8±4.0	26.1±0.6	515.0±17.8	296.4±15.2	387.0±23.0	353.0±8.9	720.8±38.5	384.0±6.5
SM C16:1	3.3±0.1	96.0±2.8	2.1±0.1	10.1±0.3	2.2±0.0	10.4±0.1	25.0±1.8	10.7±0.5	9.0±0.5	6.9±0.3	20.5±0.9
SM C18:0	5.5±0.6	4.5±0.2	90.5±2.2	34.6±1.0	394.0±10.8	476.3±12.9	29.7±1.0	69.3±4.4	113.0±3.6	23.6±1.5	78.8±2.4
SM C18:1	0.7±0.1	1.8±0.1	4.7±0.1	5.3±0.3	118.7±4.5	31.8±0.9	8.6±0.3	5.3±0.2	9.4±0.4	2.0±0.1	10.7±0.6
SM C20:2	<LOD	0.2±0.1	0.0±0.0	0.2±0.0	0.1±0.1	0.3±0.2	0.1±0.1	0.2±0.1	0.2±0.1	0.2±0.1	0.1±0.0
SM C22:3	<LOD	<LOD	<LOD	<LOD	<LOD	<LOD	<LOD	0.5±0.3	<LOD	<LOD	<LOD
SM C24:0	18.7±0.2	13.6±0.9	6.8±0.2	43.0±1.5	23.9±1.5	73.9±3.4	17.2±1.1	73.0±6.0	120.3±4.8	8.3±0.5	34.4±2.3
SM C24:1	49.3±1.8	27.3±1.3	17.6±0.5	85.6±3.6	38.0±0.7	204.5±5.9	68.0±3.1	198.5±13.5	242.3±5.4	47.1±2.0	93.7±3.3
SM C26:0	0.2±0.0	0.1±0.1	0.1±0.0	0.4±0.1	0.1±0.1	0.3±0.2	0.2±0.0	0.4±0.1	0.7±0.1	<LOD	<LOD
SM C26:1	0.1±0.1	0.1±0.0	0.1±0.0	0.4±0.1	0.4±0.1	0.4±0.2	0.3±0.1	1.0±0.3	1.0±0.1	<LOD	0.2±0.0
H1	44438.3±1510.6	1976.3±79.7	1290.7±14.6	2005.8±23.6	<LOD	415.8±17.3	3381.6±178.7	1568.5±39.5	3796.0±117.3	1569.0±71.1	1913.3±90.4

References

Salek, R. M., Steinbeck, C., Viant, M. R., Goodacre, R., & Dunn, W. B. (2013). The role of reporting standards for metabolite annotation and identification in metabolomic studies. *GigaScience, 2*(1), 13.
